# A rich fauna of subterranean short-range endemic Anillini (Coleoptera, Carabidae, Trechinae) from semi-arid regions of Western Australia

**DOI:** 10.3897/zookeys.1044.58844

**Published:** 2021-06-16

**Authors:** Pier Mauro Giachino, Stefan Eberhard, Giulia Perina

**Affiliations:** 1 World Biodiversity Association onlus. Private: via della Trinità 13, I-10010 San Martino Canavese (TO), Italy World Biodiversity Association Torino Italy; 2 Subterranean Ecology Pty Ltd, 227 Coningham Road, Coningham, TAS 7054, Australia Subterranean Ecology Pty Ltd Coningham Australia; 3 Collections and Research, Western Australian Museum, 49 Kew Street, Welshpool, WA 6106, Australia Western Australian Museum Perth Australia

**Keywords:** Drill holes, environmental impact assessment, hypogean, new genera, new species, speciation, troglobiont, zoogeography

## Abstract

Globally, the great majority of Anillini species are endogean, adapted to live in the interstices of soil and leaf litter, while the extremely low vagility of these minute ground beetles gives rise to numerous shortrange endemic species. Until recently the Australian Anillini fauna was known only from leaf litter in rain forests and eucalypt forests in the wetter, forested regions of eastern and south eastern Australia, as well as Lord Howe and Norfolk islands. The first hypogean Anillini in Australia (17 species in six genera) were described in 2016 from mineral exploration drill holes in iron-ore bearing rocks of the Pilbara region in Western Australia, representing the first finding of the tribe deep underground in a semi-arid climate region. A further eight new genera and 20 new species are described herein, mostly from the Pilbara region as well as the semi-arid Kimberley and Goldfields regions; all were collected in mineral exploration drill holes. The following new genera are described: *Erwinanillus***gen. nov.**, *Gregorydytes***gen. nov.**, *Pilbaraphanus***gen. nov.**, *Neoillaphanus***gen. nov.**, *Kimberleytyphlus***gen. nov.**, *Gilesdytes***gen. nov.**, *Pilbaradytes***gen. nov.**, and *Bylibaraphanus***gen. nov.** The following new species are described: *Erwinanillus baehri***sp. nov.**; *Gracilanillus hirsutus***sp. nov.**, *G. pannawonicanus***sp. nov.**; *Gregorydytes ophthalmianus***sp. nov.**; *Pilbaraphanus chichesterianus***sp. nov.**, *P. bilybarianus***sp. nov.**; *Magnanillus firetalianus***sp. nov.**, *M. sabae***sp. nov.**, *M. salomonis***sp. nov.**, *M. regalis***sp. nov.**, *M. serenitatis***sp. nov.**; *Neoillaphanus callawanus***sp. nov.**; *Kimberleytyphlus carrboydianus***sp. nov.**; *Austranillus jinayrianus***sp. nov.**; *Gilesdytes pardooanus***sp. nov.**, *G. ethelianus***sp. nov.**; *Pilbaradytes abydosianus***sp. nov.**, *P. webberianus***sp. nov.**; *Bylibaraphanus cundalinianus***sp. nov.**; and *Angustanillus armatus***sp. nov.** Identification keys are provided for all Australian anilline genera, and Western Australian species. All the described species are known from a single locality and qualify as short-range endemics. The Anillini are recognised as a significant and diverse element making up part of Western Australia’s remarkable subterranean fauna, and whose conservation may potentially be impacted by mining developments.

## Introduction

The subtribe Anillina was erected by Jeannel (1937) for certain minute, blind, depigmented bembidiine carabid beetles that lack a recurved striole at the apex of the elytron (Moore 1980). Zaballos et al. (2017) upgraded the subtribe Anillina to the rank of tribe Anillini, as already proposed by Jeannel (1963). The great majority of Anillini are adapted to live in the interstices of soil, and they display typical morphological adaptations of endogean fauna, such as small body size, loss of wings and eyes, and depigmentation. A few cave-dwelling and less specialised humicolous species of Anillini are also known. The extremely low vagility of these beetles gives rise to numerous short-range endemic species (Moore 1980; Giachino 2005, 2008, 2015; Giachino and Vailati 2011; Baehr and Main 2016; Andújar et al. 2017; Pérez-González et al. 2018). Anillini have a scattered worldwide distribution (Jeannel 1937, 1963) and form a monophyletic group (Andújar et al. 2016) with Gondwanan affinities (Jeannel 1937, 1963; Giachino 2005). Globally 74 genera and 543 species are currently described (Lorenz 2020).

For many years the Australian Anillini fauna was known from only two described species in eastern Australia: *Illaphanus stephensi* Macleay, 1865 and *Austranillus macleayi* (Lea, 1906). The genus *Illaphanus* was redescribed by Jeannel (1937, 1963; see Moore et al. 1987), and more recently one of the Australian species initially assigned to *Illaphanus* was recognised as a separate new genus (Giachino 2005). Giachino’s extensive studies of existing museum collections, combined with new field collections transformed understanding about Australia’s apparently depauperate anilline fauna, which was revealed to be much more diverse than previously recognised. Giachino (2005) recognised three new genera and 34 species, all from eastern Australia. All of these species were collected from leaf litter in rain forest and eucalypt forest in the wetter, forested regions of eastern and south eastern Australia, extending from Queensland to Victoria and Tasmania, as well as Lord Howe Island and Norfolk Island. Another litter dwelling species belonging to a new genus, sampled using Berlese extraction in Darwin City (Northern Territory), was described by Baehr (2018). All of these species displayed typical morphological characters of endogean fauna and had restricted distribution ranges. No truly hypogean (troglobitic) species have yet been recognised from eastern Australia, which possibly reflects the lack of targeted sampling for these minute beetles in caves and other cryptic hypogean environments including shallow subterranean habitats (sensu Giachino and Vailati 2010; Culver and Pipan 2014), which are difficult to access and sample. Regarding shallow subterranean habitats, it is relevant to note that Moore (1980) described a species that he regarded as subterranean, *Hygranillus kuscheli*, which was collected from a dry well slotted at 4.2 m depth in Brightwater, New Zealand. The first troglobitic Anillini recorded in Australia were collected from mineral exploration drill holes in iron-ore bearing rocks of the semi-arid Pilbara region in Western Australia (WA), representing the first collections of Anillini in WA, and the first finding of the tribe deep underground in a semi-arid region, thus also significantly extending knowledge about the groups’ ecology (Baehr and Main 2016). Six new genera and 17 new species, all blind and depigmented, were described by Baehr and Main, and three of the genera had decidedly larger body size, a characteristic often associated with adaptative shift from an interstitial to a cavicolous existence. In this paper we describe a further eight new genera and 20 new species, all collected in mineral exploration drill holes, and provide identification keys to all Australian anilline genera, and West Australian species. With this contribution we further expand the morphological taxonomic framework and understanding of distribution patterns, and highlight the rich diversity of Anillini species and genera in the semi-arid zones of WA. All of the described species are known from a single locality and qualify as short-range endemics (SRE’s) (sensu Harvey 2002) and are therefore of conservation significance. This contribution firmly establishes the Anillini as a significant and diverse element making up part of WA’s remarkable subterranean fauna, and whose conservation status may potentially be impacted by mining developments.

## Materials and methods

The material studied was collected by Subterranean Ecology Pty Ltd and other environmental consultancies during field surveys for subterranean fauna conducted between 2008 and 2011 as part of environmental impact assessments (EIAs) for proposed mining developments. During these surveys access to hypogean habitats is temporarily available via numerous mineral exploration drill holes which are constructed by mining companies during the course of their geological investigations. The uncased drill holes, mostly around 100 mm diameter, penetrate the geological strata from the surface to considerable depths, sometimes > 100 m and frequently intersecting the groundwater table. The drill holes are colonised by hypogean invertebrates which enter the hole from the surrounding permeable rock matrix, as well as endogean and epigean invertebrates which wander or accidentally fall into the drill hole, especially if the entrance of the drill hole is uncapped. Collections from drill holes often contain a mixture of hypogean, endogean and epigean taxa. Following the mining exploration phase most drill holes are filled in as part of mandatory rehabilitation processes.

All the specimens in this study were collected from drill holes. Nearly all drill holes were located in iron-ore bearing rocks in the Pilbara region in northwest WA where the climate is hot semi-arid / hot desert (according to the Köppen classification). Two sampling localities occurred outside the Pilbara region: iron-ore bearing rocks in the Carr-Boyd Ranges in the hot semi-arid Kimberley region, northern WA; and granite/basalt rocks near Forrestania in the cold semi-arid Goldfields region, southwest WA (Fig. 52).

Invertebrates were collected from drill holes by trapping and ‘scraping’, two standard methods used for sampling troglofauna in drill holes. These methods conformed with the WA Environmental Protection Authority (EPA) Guidance Statements for subterranean fauna No. 54 (EPA 2003) and 54a (EPA 2007).

The trapping method involved lowering one or two PVC tube traps of 70 mm diameter and approximately 150 mm in length into the drill hole and leaving them in place for six to eight weeks to be colonised by fauna. The traps were filled with locally collected vegetation to provide shelter, moisture and a possible food source that may be attractive to invertebrates. The vegetation, mostly *Spinifex* grass was sterilised in a microwave beforehand to kill any surface invertebrates and microorganisms. Occasionally traps were baited with cheese, meat, or dog biscuit. The PVC tubes were hung vertically on venetian blind cord. The bottom of the tube was sealed with a PVC end cap which had a small hole drilled in the middle to drain excess water. The top of the tube was covered with bird mesh to retain the vegetation while allowing access for invertebrates. Occasionally two traps were placed at different depths in each drill hole, to test if trapping success was related to trap depth (results not reported here). The traps in this study were deployed at depths of 8–58 m below ground level. After placement of the trap, the drill hole was re-capped to maintain humidity within the hole. Six to eight weeks later the traps were retrieved and placed in separate labelled zip-lock bags and transported in a portable cooler to the laboratory for fauna extraction. Invertebrates were extracted from the leaf litter using Berlese/Tullgren funnels over a period of at least 24 hours or until the leaf litter was completely dried. Specimens were preserved in 100% ethanol.

The ‘scraping’ method utilises a small plankton net of the same type as commonly used for sampling groundwater fauna in bores and wells. The net was constructed of fine mesh < 250 µm and of slightly smaller diameter than the drill hole, had a reinforced leading edge, and weighted vial attached at the bottom. The net was lowered to the base of the hole or just below the water-table surface and then dragged back up against the wall of the drill hole to dislodge and capture invertebrates from the walls. Scraping was conducted at least four times in each hole, with each scrape made along a different quarter of the drill hole. At some sites scraping efficiency may have been improved through the use of a ‘tickler’ device which resembled a bottle brush and was attached just above the net to scrape all quarters of the hole on each haul. The contents of the net were emptied into a small bucket of water after each haul. At conclusion of hauling the bucket contents were elutriated and sieved, and the sample preserved in ethanol for transport to the laboratory for processing.

Most Anillini specimens in this study were collected by trapping. Some of the material comprised incomplete or damaged specimens, an inherent limitation with the scraping collection method, which also collects dead and partially decomposed animals from the drill hole walls and water-table surface. Despite this limitation, enough material was collected by both scraping and trapping methods to enable adequate morphological descriptions. Further details on trapping and scraping methods are given in Halse and Pearson (2014).

Morphological analysis was conducted on whole specimens and dissected male genitalia mounted in Canada balsam. Use of Canada balsam facilitated the observation of important diacritic characters which are otherwise difficult to observe in such small specimens as the Anillini. All drawings were made using a drawing tube on a Leica DM2500 biological microscope with phase contrast. One specimen for each taxon was prepared and drawn.

The Anillini collection described here was sent to the principal author in two batches, 2009 and 2011, while later, additional material, some of which was collected from the same localities, was sent to the taxonomist Martin Baehr (München, Germany). Baehr and Giachino were unaware of the other’s collections and so they worked independently on their material that represented in part the same genera, thus running the risk to publish synonyms. Fortunately, both taxonomists became aware of the situation and thus avoided any nomenclatural publication issues. Due to different study techniques used by each author some interpretation difficulties became apparent and are noted in this paper.

### Acronyms

The following acronyms for museums or private collections have been used:

**CGi** Collection Giachino, San Martino Canavese (TO), Italy.

**WAM**Western Australian Museum, Perth, Western Australia.

The following acronyms for the type material have been used:

**HT** Holotype.

**PT**, **PTT** Paratype (s).

The following acronyms for measurement have been used:

**TL** total length, from the anterior margin of the labrum to the end of the elytra.

## Systematics

We provide an updated key to the genera of Australian Anillini, modified from Giachino (2005) and Baehr and Main (2016), and including the genus *Parillaphanus* described by Baehr (2018) and the new genera described in this paper.

### Key to the genera of Australian Anillini

**Table:** 

1	Metatrochanters very long and sharp	**2**
–	Metatrochanters normal	**4**
2	Metafemora dentate, elytra bearing three discal setae	**3**
–	Metafemora not dentate, elytra bearing one discal seta	***Neoillaphanus* gen. nov.**
3	Elytral disc without longitudinal grooves	***Pilbaraphanus* gen. nov.**
–	Elytral disc with two distinct longitudinal grooves, running between the scutellar pore and the 9^th^ pore of the umbilicate series	***Magnanillus* Baehr**
4	Elytral disc with two distinct longitudinal grooves, running between the scutellar pore and the 9^th^ pore of the umbilicate series	**5**
–	Elytral disc without longitudinal grooves	**7**
5	Elytra more or less oval-shaped, narrowed to base, humerus oblique or rounded	**6**
–	Elytra parallel-sided or almost so, little or not narrowed to base, humerus almost rectangular. North-western Australia: Pilbara	***Gracilanillus* Baehr & Main**
6	Metafemora dentate (at least in male), elytra bearing only one discal seta. Central southern Western Australia	***Erwinanillus* gen. nov.**
–	Metafemora not dentate, elytra bearing one or two discal setae. Eastern Australia	***Illaphanus* Macleay**
7	Elytra provided with a supplementary umbilicate pore, placed at ca. the level of the 2^nd^ pore of the umbilicate series	***Tasmanillus* Giachino**
–	Elytra without any supplementary umbilicate pore	**8**
8	Species from Northern Territory	***Parillaphanus* Baehr, 2018**
–	Species from Western Australia	**9**
9	Species from Pilbara: Western Australia	**10**
–	Species from central southern Western Australia	***Externanillus* Baehr & Main**
10	Basal border of pronotum strongly wider than anterior border	***Pilbaranilllus* Baehr & Main**
–	Basal border of pronotum less wide or as wide as the anterior border	**11**
11	Body size large, length > 1.35 mm; eye area markedly prominent, head as wide as pronotum; pronotum cordiform	***Hesperanillus* Baehr & Main**
–	Species with a different set of characters	**12**
12	Ninth pore of the umbilicate series in normal position, placed after the 8^th^ one	**13**
–	Ninth pore of the umbilicate series in abnormal position, placed before the 8^th^ one	**14**
13	Elytra reduced at tip with last abdominal tergites uncovered. Basal border of the pronotum remarkably narrower than the anterior border, sides of the pronotum distinctly sinuate posteriorly	***Gregorydytes* gen. nov.**
–	Elytra not reduced at tip with last abdominal tergites covered. Basal border of the pronotum ca. as wide as the anterior border, sides of the pronotum not sinuated posteriorly	***Kimberleytyphlus* gen. nov.**
14	Body very elongate (ratio length/width of elytra > 2.20; ratio width/length of pronotum ≤ 0.90). Elytra with four distinct striae, or with longitudinally aligned pubescence. Metafemora dentate (almost dentate in males)	***Angustanillus* Baehr & Main**
–	Body less elongate (ratio length/width of elytra < 2.15; ratio width/length of pronotum > 0.97). Elytra without distinct striae or longitudinally aligned pubescence. Metafemora not dentate	**15**
15	Pronotal posterior seta absent	***Pilbaradytes* gen. nov.**
–	Pronotal posterior seta present	**16**
16	Basal border of the pronotum remarkably narrower than the anterior border; sides of the pronotum distinctly sinuate posteriorly	***Pseudillaphanus* Giachino**
–	Basal border of the pronotum ca. as wide as, or only slightly narrower, than the anterior border; sides of the pronotum not, or only very poorly, sinuated posteriorly	**17**
17	Elytral disc with two setigerous punctures	***Austranillus* Giachino**
–	Elytral disc with one setigerous puncture	**18**
18	Pronotum lateral sides regularly curved from anterior to posterior angles. Elytra with discal puncture situated at the same level as, or after, 7^th^ umbilicate pore	***Gilesdytes* gen. nov.**
–	Pronotum lateral sides not regularly curved, more or less sinuate before the posterior angles. Elytra with discal puncture situated well before 7^th^ umbilicate pore	***Bylibaraphanus* gen. nov.**

#### Erwinanillus gen. nov.

Taxon classificationAnimaliaColeopteraCarabidae

0F079F93-A8BA-5D16-9D03-2A412253679E

http://zoobank.org/E495BCA5-289A-4D5A-A98D-3C5093FBE905

Figs 1–3

##### Type species.

*Erwinanillus baehri* sp. nov.

##### Diagnosis.

Included species strongly characterised by the presence of longitudinal elytral grooves; male protarsi with two dilated tarsomeres and profemora unarmed; metafemora dentate and metatrochanters short and stout in male and female. Labial tooth absent. Head characterised by temples with a series of long excess setae. One elytral discal seta present. Aedeagus with right paramere bearing two apical setae; left bearing only one seta. This genus can be distinguished from *Externanilus* Baehr & Main, 2016 by the presence of grooves on elytral disc.

##### Description.

Included species of small-medium size (TL mm 1.40–1.42), anophthalmous. Depigmented and poorly sclerified integument covered with a sparse pubescence.

***Head*** robust, almost hypertrophic, slightly narrower than the pronotum, with a series of excess long setae; labium toothless, mentum articulated, not fused with the submentum. Antennae moniliform, without particular features.

***Pronotum*** subquadrate, with sides distinctly sinuate in the basal third. Basal angles subsquare and acuminate; basal border as wide as the anterior border; presence of two marginal setae, the posterior one placed before the basal angles.

***Elytra*** oval and slightly elongated, separately rounded, not truncate and not emarginated apically; poorly convex and with a longitudinal groove. Elytral striae missing (except for the sutural stria). Lateral margin, distinctly crenelated from the humeral area to the apical third. Scutellar pore present, large and umbilicate; umbilicate series of type B (sensu Jeannel 1963; Giachino and Vailati 2011); disc bearing one discal seta.

***Legs*** relatively short and stumpy. Profemora unarmed, metafemora dentate, metatrochanters normal, two dilated protarsomeres in the male.

***Aedeagus*** small, median lobe moderately elongated, apex acuminate with apical blade very evident. Parameres stocky, bearing one apical seta (left) or two apical setae (right). Endophallus without sclerified phanerae.

##### Etymology.

Dedicated to the memory of Terry Lee Erwin, renowned carabid beetle specialist and explorer of world biodiversity. Name composed of Erwin and the genus name *Anillus*. The gender of the name is masculine.

##### Species included.

For the time being, the genus is monotypic and only *E. baehri* sp. nov. belongs to this genus.

**Figures 1–3.**
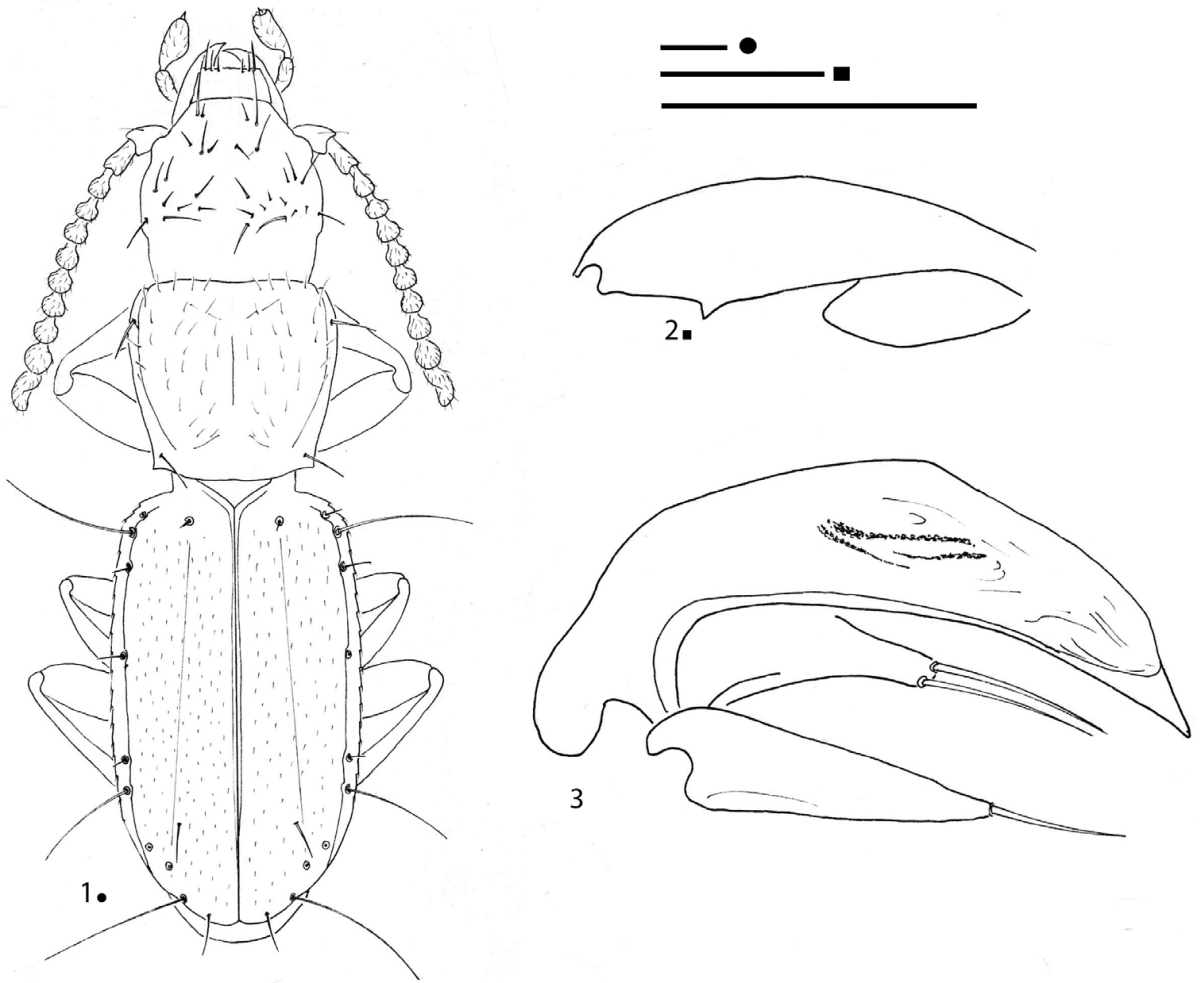
*Erwinanillus baehri* gen. et sp. nov., HT ♂. **1** habitus **2** right metafemur and metatrochanter in ventral view **3** aedeagus in left lateral view. Scale bars: 0.1 mm.

#### Erwinanillus baehri sp. nov.

Taxon classificationAnimaliaColeopteraCarabidae

AEBF67BA-5DD6-50E0-B257-D501BA4B4B1C

http://zoobank.org/EA033DC4-CCC6-4F3B-95EA-B6EED9ED84C8

Figs 1–3

##### Type locality.

WA, Southern Goldfields region, Forrestania, 80 km E of Hyden, Cosmic Boy Mine, 32°29'19"S, 119°40'49"E.

##### Type series.

HT ♂, WA, Southern Goldfields region, Forrestania, 80 km E of Hyden, Cosmic Boy Mine, 32°29'19"S, 119°40'49"E, 06 April 2009, Rockwater, bore WWMB4, hauling. Western Australian Museum Entomology Reg. no. 72025 (WAM). PTT: 1 ♀ (remains), WA, Southern Goldfields region, Forrestania, 80 km E of Hyden, Cosmic Boy Mine, 32°29'18.84"S, 119°40'48.69"E (WGS84), Rockwater, 6 April 2009, Stygo net haul. (RW-WWMB4-LN5105), Western Australian Museum Entomology Reg. no. 82637 (WAM); 1 spec. (remains), WA, Southern Goldfields region, Forrestania, 80 km E of Hyden, Cosmic Boy Mine, 32°29'18.84"S, 119°40'48.69"E (WGS84), Rockwater, 6 April 2009, Stygo net haul. (RW-WWMB4-LN5105), Western Australian Museum Entomology Reg. no. 82638 (CGi).

##### Diagnosis.

Small species, with longitudinal elytral grooves; male protarsi with two dilated tarsomeres and profemora unarmed; metafemora dentate and metatrochanters short and stout in male and female. Head characterised by temples with a series of excess long setae. One elytral discal seta present. Aedeagus with right paramere bearing two apical setae; left bearing only one seta.

##### Description.

TL 1.40–1.42 mm. ***Body*** elongated, depigmented, fulvo-testaceous with elytra and abdomen lighter, yellow-testaceous; integument shiny with evident microsculpture and spread pubescence.

***Head*** robust, almost hypertrophic, slightly narrower than the pronotum. Labium toothless, mentum articulate. Antennae robust, moniliform, short, not exceeding the base of the pronotum when stretched backwards. Fronto-clypeal furrow indistinct; anterior margin of the epistome subrectilinear.

***Pronotum*** subquadrate (max. width / max. length ratio = 1.05), with the maximum width on the anterior fourth, narrowed at the base, with the sides poorly arcuate anteriorly, sinuate before the base. Anterior angles rounded, weakly prominent; posterior angles subsquare and acuminate at tips. Disc convex, with a long and sparse pubescence; median groove very shallow, hardly evident. Marginal groove wide and flat, slightly enlarged near the base; anterior marginal setae placed inside the marginal groove, almost on the anterior fifth; basal setae before the posterior angles.

***Legs*** short and stout, two protarsomeres dilated and without adhesive phanerae in male. Metatrochanters short and stout, metafemora dentate (Fig. 2).

***Elytra*** oval, slightly elongated (max. length / max. width ratio = 1.72), with maximum width in the middle, not emarginated before apex. Disc poorly convex, provided with an evident longitudinal groove running more or less between the scutellar pore and the 9^th^ pore of the umbilicate series; integument shiny, with evident microsculpture and short, dense, upright pubescence. Humeri poorly marked, rounded. Post-humeral margin denticulate, with a very thin but distinct crenulation to the apical third; elytral apices separately rounded. Marginal groove wide and evident almost to the 8^th^ pore of the umbilicate series.

***Chaetotaxy***: scutellar pore large, foveate. Umbilicate series with the first three pores of the humeral group not equidistant, with 1^st^ and 2^nd^ closer together than 2^nd^ and 3^rd^; 4^th^ pore clearly farther and placed at the end of the basal third of the elytron; 5^th^ pore placed before the apical third of the elytra; 5^th^ and 6^th^ ones spaced out ca. half distance between the 6^th^ and 7^th^; 7^th^ slightly and 8^th^ nearly moved onto the disc; 7^th^ and 8^th^ slightly closer to each other than the 8^th^ and 9^th^. One discal seta, placed before the 7^th^ pore of the umbilicate series.

***Aedeagus*** (Fig. 3) small, abruptly arcuate in the basal part; median lobe moderately elongated with ventral margin gently arcuate up to the acuminate apex, with apical blade very evident. Endophallus without an evident lamella copulatrix. Parameres stocky in the basal part and relatively poorly elongated, not reaching the distal third. Right paramere bearing two apical setae; left bearing only one seta.

##### Etymology.

Dedicated to the memory of Martin Baehr, renowned beetle taxonomist, in honour of his contributions to the knowledge of Australian ground beetles.

##### Distribution.

*Erwinanillus baehri* sp. nov. is known only from bore WWMB4, 80 km E of Hyden, Forrestania, Southern Goldfields region, WA.

#### Gracilanillus

Taxon classificationAnimaliaColeopteraCarabidae

Baehr & Main, 2016

C317777F-87C4-5FBD-AC38-F84390423925

Figs 4–7

##### Type species.

*Gracilanillus longulus* Baehr & Main, 2016.

##### Note.

Baehr and Main’s description (2016) is correct and detailed, although we noted a few inaccuracies and their description omits some important characters fundamental to the systematics of Anillini which are described as follows. Baehr and Main (2016) describe three setae on the elytral disc of *Gracilanillus* genus, and later in the paper they specify that *G. longulus* Baehr & Main, 2016, *G. cockingi* Baehr & Main, 2016 and *G. cordatus* Baehr & Main, 2016 bear only two setae, *G. vixsulcatus* Baehr & Main, 2016 bear only one seta, and that in *G. minutus* Baehr & Main, 2016 and *G. currani* Baehr & Main, 2016 have none. The two additional *Gracilanillus* species that we describe herein bear two setae, and we suggest that considering the difficulty of observing all setae if specimens are not examined using the methods as described in this paper, possibly all species in this genus share the character “two setae on the elytral disc”.

Baehr and Main (2016) refer to a “faint” labial tooth, while the two species described here do not possess this character. The labial tooth is not an important character at genus level (Giachino 2005; Giachino and Vailati 2011), and also it is very difficult to see if the specimen is not properly prepared. Baehr and Main (2016) do not mention the position of the elytral umbilicate setae, but only talk about “very long setae”. The position of these setae is fundamental (Jeannel 1963; Giachino 2005; Giachino and Vailati 2011), hence we specify the position, type “B” (sensu Jeannel 1963; Giachino and Vailati 2011). For additional details see descriptions.

##### Species included.

Seven species currently belong to this genus:

*Gracilanillus hirsutus* sp. nov.

*Gracilanillus pannawonicanus* sp. nov.

*Gracilanillus longulus* Baehr & Main, 2016

*Gracilanillus cockingi* Baehr & Main, 2016

*Gracilanillus cordatus* Baehr & Main, 2016

*Gracilanillus vixsulcatus* Baehr & Main, 2016

*Gracilanillus currani* Baehr & Main, 2016

**Figures 4–7.**
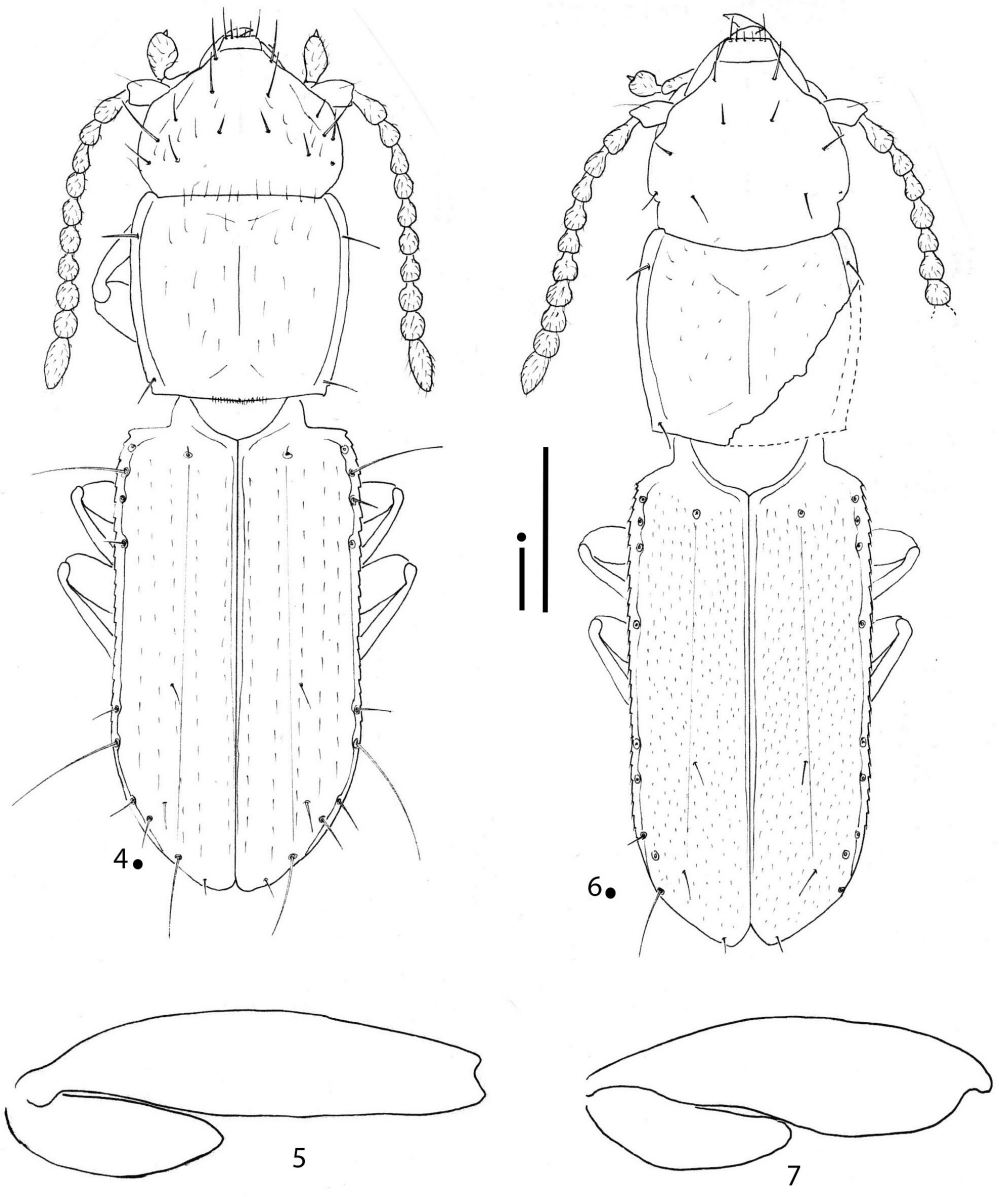
*Gracilanillus* spp.: habitus HT ♀ (**4, 6**) left metafemur and metatrochanter in ventral view HT ♀ (**5, 7**) **4, 5***G. hirsutus* sp. nov. **6, 7***G. pannawonicanus* sp. nov. Scale bars: 0.1 mm.

### Key to the species of *Gracilanillus* (modified from Baehr and Main 2016)

**Table:** 

1	Body size small, length < 1.20 mm, aedeagus unknown	**2**
–	Body size large, length ≥ 1.40 mm	**3**
2	Elytra long and narrow, ratio length/width 2.04; pronotum wide, ratio width/length 1.11, with wide base, ratio base/apex 0.96, sides not sinuate near base	***G. minutus* Baehr & Main**
–	Elytra short and wide, ratio length/width 1.95; pronotum narrow, ratio width/length 1.02, with narrow base, ratio base/apex 0.79, sides sinuate near base	***G. cordatus* Baehr & Main**
3	Elytra very narrow and elongate, ratio length/width > 2.13; body size slightly smaller, length < 1.50 mm; basal half of pronotum not sinuate	**6**
–	Elytra slightly wide and short, ratio length/width ≤ 2.08; body size slightly large	**4**
4	TL > 1.58 mm; basal half of pronotum faintly sinuate	***G. cockingi* Baehr & Main**
–	TL < 1.58 mm; basal half of pronotum not or faintly sinuate	**5**
5	Elytral disc with long and sparse pubescence distinctly aligned lengthwise. Small head with many excess setae. Ninth pore of the umbilicate series in standard position (Fig. 4)	***G. hirsutus* sp. nov.**
–	Elytral disc with short and dense pubescence not aligned lengthwise. Head without excess setae. Ninth pore of the umbilicate series anterior compare to the standard position (Fig. 6)	***G. pannawonicanus* sp. nov.**
6	Pronotum wide, ratio length/width 1.14, very shortly excised immediately in front of base	***G. currani* Baehr & Main**
–	Pronotum narrow, ratio length/width < 1.0; not excised in front of base	**7**
7	Base of pronotum relatively wide, ratio base/apex 0.98, basal angle almost rectangular; elytral sulcus faint and only in basal third noticeable	***G. vixsulcatus* Baehr & Main**
–	Base of pronotum relatively narrow, ratio base/apex 0.88; basal angle obtuse; elytral sulcus distinct and complete	***G. longulus* Baehr & Main**

#### Gracilanillus hirsutus sp. nov.

Taxon classificationAnimaliaColeopteraCarabidae

C7332E33-8BED-5DA7-89E6-B481AEF6CE03

http://zoobank.org/5EFB578E-C7E7-4557-A24F-40B7372DDDBB

Figs 4–5

##### Type locality.

WA, Pilbara, 44 km W of Pannawonica, Robe Valley, Mesa A Mine, 21°39'52"S, 115°53'48"E.

##### Type series.

HT ♀, WA, Pilbara, 44 km W of Pannawonica, Robe Valley, Mesa A Mine (bore MEARC4038), 21°39'52"S, 115°53'48"E (GPS), 10 June–3 Aug. 2006; litter trap L. Mould, M. Greenham; Western Australian Museum Entomology Reg. no. 64216 (WAM).

##### Differential diagnosis.

*Gracilanillus hirsutus* sp. nov. can be distinguished from *G. pannawonicanus* sp. nov. in the presence of long and sparse pubescence longitudinally aligned on the elytral disc, head with many excess setae, and for the standard position of the ninth pore of the umbilicate series.

##### Description of the HT ♀.

TL 1.39 mm. ***Body*** elongated, depigmented, yellow-testaceous; shiny integument with evident microsculpture and pubescence.

***Head*** robust, hypertrophic, slightly narrower than pronotum; bearing a number of excess setae. Labium toothless, mentum articulate. Antennae robust, moniliform, and short, not exceeding the base of the pronotum when stretched backwards. Fronto-clypeal furrow indistinct; anterior margin of the epistome subrectilinear.

***Pronotum*** subquadrate (max. width / max. length ratio = 1.05), with maximum width in the middle, and with basal border as wide as anterior border; sides slightly but regularly arcuate from anterior to posterior, not sinuate, but strictly emarginated before the basal angles. Anterior angles rounded, poorly prominent; posterior angles subsquare. Disc convex, with long and sparse pubescence; median groove very shallow, hardly evident. Marginal groove wide and flat, slightly enlarged near the base; anterior marginal setae placed inside marginal groove, almost on anterior fifth; basal setae almost before the posterior angles.

***Legs*** short and stout, with metatrochanters short and stout and metafemora not dentate (Fig. 5).

***Elytra*** subrectangular, elongated (max. length / max. width ratio = 1.84), with maximum width at the base of the posterior third, not emarginated before apex. Disc slightly convex, with evident longitudinal groove running more or less between the scutellar pore and the 9^th^ pore of the umbilicate series; integument shiny with evident microsculpture and long, sparse, upright pubescence, longitudinally aligned. Humeri well marked; post-humeral margin denticulate, with distinct crenulations all the way to the apical third; elytral apices separately rounded. Marginal groove wide and evident almost up to the 9^th^ pore of the umbilicate series.

***Chaetotaxy***: scutellar pore large, foveate. Umbilicate series with first three pores of humeral group equidistant; 4^th^ pore farther and placed at the end of the basal fourth of elytron; 5^th^ pore placed before the apical third of elytra; 5^th^ and 6^th^ ones spaced out ca. half distance of 6^th^ and 7^th^; 8^th^ slightly displaced on the disc; 7^th^ and 8^th^ closer to each other than 8^th^ and 9^th^. Two discal setae, first placed before the 5^th^ pore of the umbilicate series, second one placed just before 8^th^ pore.

**Male.** Unknown.

##### Etymology.

The name comes from the Latin *hirsutus* (= hairy, hirsute) to recognise the presence of many excess setae on the head.

##### Distribution.

*Gracilanillus hirsutus* sp. nov. occurs only at the type locality in Mesa A Mine, Robe River Valley, 44 km W of Pannawonica, Pilbara, WA.

#### Gracilanillus pannawonicanus sp. nov.

Taxon classificationAnimaliaColeopteraCarabidae

A7FAEFEA-BA3A-53A9-BB71-5189EB98A1DC

http://zoobank.org/1A567BDA-8F9A-45F5-AB5A-D637E6AAFCDB

Figs 6–7

##### Type locality.

WA, Pilbara, 11 km SSE of Pannawonica, Robe Valley, Mesa K Mine, 21°43'11"S, 116°15'43"E.

##### Type series.

HT ♀, WA, Pilbara, 11 km SSE of Pannawonica, Robe Valley, Mesa K Mine, (bore MEK1701), 21°43'11"S, 116°15'43"E (GPS), 10 June-3 Aug. 2006; litter trap L. Mould, M. Greenham; Western Australian Museum Entomology Reg. no. 64215 (WAM).

##### Differential diagnosis.

*Gracilanillus pannawonicanus* sp. nov. can be distinguished from *G. hirsutus* for the presence of a short and dense pubescence on the elytral disc, not directly longitudinally aligned, for the absence of excess setae on the head, and for the position of the ninth pore of the umbilicate series, which is placed quite forward, before the end of the elytral groove.

##### Description of the HT ♀.

TL 1.43 mm. ***Body*** elongated, depigmented, yellow-testaceous; shiny integument with evident microsculpture and pubescence.

***Head*** robust, almost hypertrophic, slightly narrower than pronotum, excess setae absent. Labium toothless, articulated mentum. Antennae robust, moniliform, short, not exceeding the base of the pronotum when stretched backwards. Fronto-clypeal furrow indistinct; anterior margin of the epistome subrectilinear.

***Pronotum*** subquadrate (max. width / max. length ratio = 1.07), maximum width at the basal anterior third, and basal border as wide as anterior border; sides slightly but regularly arcuate from anterior to posterior angles, strictly sinuate just before basal angles. Anterior angles rounded, slightly prominent; posterior angles subsquare and acuminate at vertex. Disc convex, with short and very sparse pubescence; median groove very shallow, hardly evident. Marginal groove wide and flat, slightly enlarged near the base; anterior marginal setae placed inside the marginal groove, almost on the anterior fifth; basal setae nearly before posterior angles.

***Legs*** short and stout, with metatrochanters short and stout and metafemora non dentate (Fig. 7).

***Elytra*** subrectangular, elongated (max. length / max. width ratio = 1.95), with maximum width in the middle, not emarginated in preapical zone. Disc slightly convex, with evident longitudinal groove running more or less between the scutellar pore and the 2^nd^ discal pore; integument shiny, with evident microsculpture, and short, very dense, and upright pubescence, not longitudinally aligned. Humeri well marked but rounded; post-humeral margin denticulate, with distinct crenulation up to the apical fourth; elytral apices separately and acutely rounded. Marginal groove wide and evident almost all the way to the 8^th^ pore of the umbilicate series.

***Chaetotaxy***: scutellar pore large, foveate. Umbilicate series with the first three pores of the humeral group equidistant; 4^th^ pore farther and placed at the end of the basal third of the elytron; 5^th^ pore placed before the apical third of the elytra; 5^th^ and 6^th^ ones spaced out ca. half of the distance between 6^th^ and 7^th^; 8^th^ slightly displaced onto the disc; 7^th^ and 8^th^ slightly closer to each other than the 8^th^ and 9^th^; 9^th^ pore of the umbilicate series placed well forward (compare to the standard position). Two discal setae, first placed just after the 5^th^ pore of the umbilicate series, second one placed just after the 8^th^ pore.

**Male.** Unknown.

##### Etymology.

The name of the species derives from the town Pannawonica near the type locality Mesa K Mine, in the Pilbara region.

##### Distribution.

*Gracilanillus pannawonicanus* sp. nov. is known so far only from the type locality Mesa K Mine, in the Robe River Valley, 11 km SSE of Pannawonica, Pilbara, WA.

#### Gregorydytes gen. nov.

Taxon classificationAnimaliaColeopteraCarabidae

29F4C0B5-ECE3-59A5-92A5-B6EFD57D8101

http://zoobank.org/4C46E75E-6238-45ED-8A3F-83013708366C

Figs 8–9

##### Type species.

*Gregorydytes ophthalmianus* sp. nov.

##### Diagnosis.

Genus characterised by normal metatrochanters, elytra reduced at tip and lacking longitudinal grooves, 9^th^ pore of the umbilicate series in normal position (placed after the 8^th^ one) and two discal setae; basal border of pronotum remarkably narrower than anterior border and sides distinctly sinuate before the basal angles; aedeagus with median lobe subrectilinear, basal bulb extremely reduced and parameres bearing one apical seta. Labial tooth lacking.

##### Description.

Species small (TL mm 1.02–1.05) and anophthalmous. Integument depigmented, poorly sclerified, and covered with sparse pubescence.

***Head*** of normal size, narrower than pronotum; mandibles short and simple, without hyperplasias. Maxillary palpi ovoidal, swollen. Labium transverse, articulated; mentum not fused with submentum. Labial tooth absent. Antennae moniliform, without particular features.

***Pronotum*** slightly transverse, with sides distinctly sinuate at the basal third. Basal angles obtuse and rounded; basal border remarkably narrower than anterior border; presence of two marginal setae, the posterior one placed at the basal angles.

***Elytra*** subrectangular and elongated, separately rounded, depressed and without longitudinal groove, and slightly truncated but not emarginated at apex. Elytral striae absent (except sutural stria). Lateral margin starting from the humeral area, distinctly crenulate to at least half-length. Scutellar pore present, large and umbilicate; umbilicate series of type B (sensu Jeannel 1963; Giachino and Vailati 2011); disc bearing two discal setae.

***Legs*** relatively short and stumpy. Unarmed pro- and metafemora, normal metatrochanters, two dilated protarsomeres in the male.

***Aedeagus*** small, median lobe short and subrectilinear with basal bulb extremely reduced. Parameres long, bearing one apical seta. Endophallus without sclerified phanerae.

##### Etymology.

*Gregorydytes* is a compound noun: in honour of Francis Thomas Gregory, the first European explorer who discovered Ophthalmia Range (type locality) in 1876, and *dytes* meaning diver in Greek. Gender name masculine.

##### Species included.

Only *G. ophthalmianus* sp. nov. belongs to this new genus.

**Figures 8, 9.**
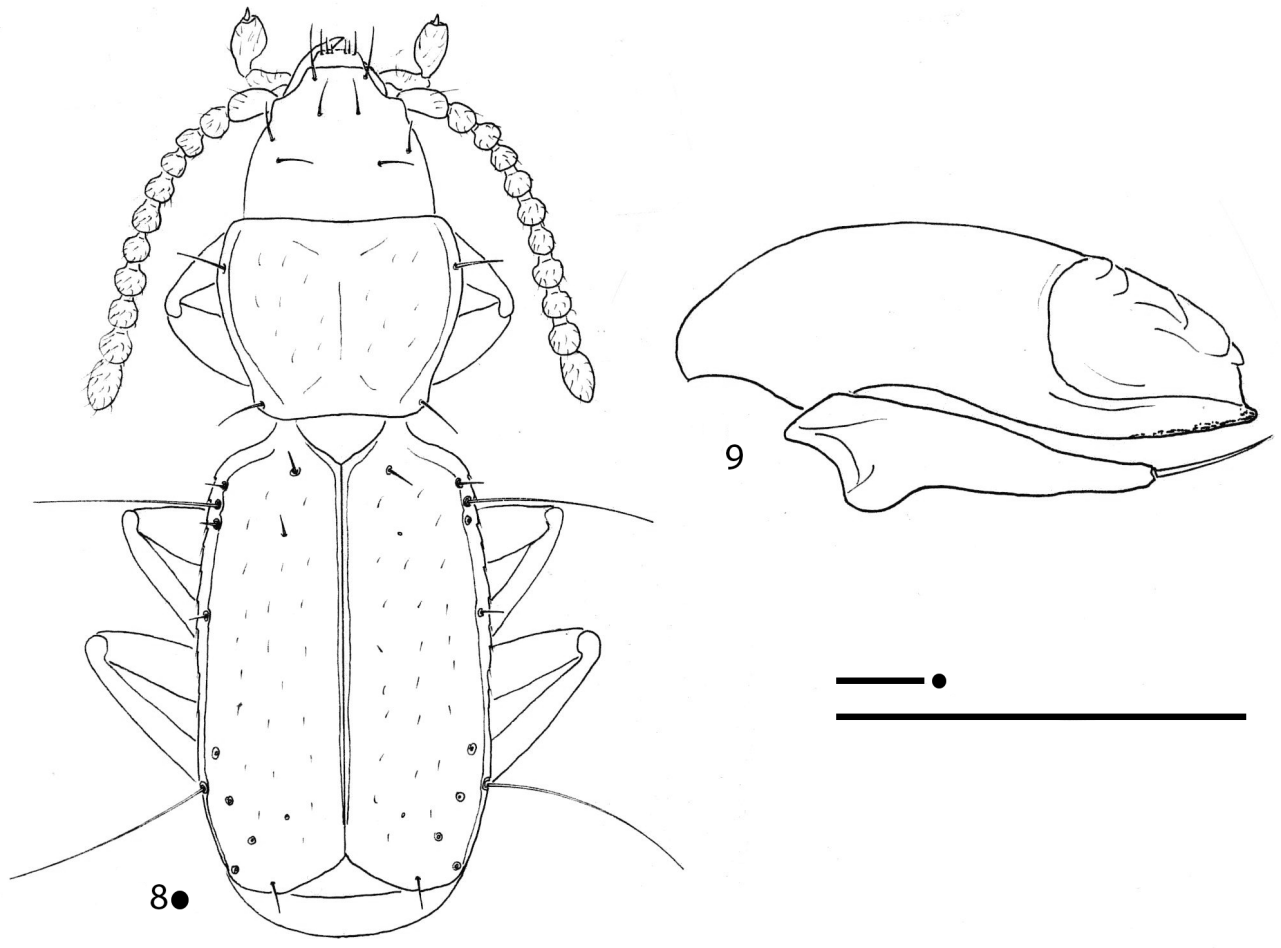
*Gregorydytes ophthalmianus* gen. et sp. nov., HT ♂ **8** habitus **9** aedeagus in lateral view. Scale bars: 0.1 mm.

#### Gregorydytes ophthalmianus sp. nov.

Taxon classificationAnimaliaColeopteraCarabidae

C8337997-043B-5A92-A283-BDEAC87F39D3

http://zoobank.org/B314CCE3-C2BC-4C80-9D37-FCA404E397DA

Figs 8–9

##### Type locality.

WA, Pilbara, 25 km W of Newman, Ophthalmia Range, 23°16'41.39"S, 119°24'13.28"E.

##### Type series.

HT ♂, WA, Pilbara, 25 km W of Newman, Ophthalmia Range, 23°16'41.39"S, 119°24'13.28"E (WGS84), P. Bell, N. Coen, 25 June 2010, Trog. net scrape; (BHP020_EXR1691-LN:9124), Western Australian Museum Entomology Reg. no. 82611 (WAM). PTT: 1 ♀, WA, Pilbara, 25 km W of Newman, Ophthalmia Range, 23°16'41.39"S, 119°24'13.28"E (WGS84), P. Bell, N. Coen, 14 August 2010, Trog. net scrape (BHP020_EXR1691-LN:9362), Western Australian Museum Entomology Reg. no. 82612 (CGi).

##### Differential diagnosis.

*Gregorydytes ophthalmianus* sp. nov. differs from other Australian Anillini by the characters highlighted in the genus diagnosis.

##### Description.

TL mm 1.02 ♂–1.05 ♀. ***Body*** moderately elongated, depigmented, yellow-testaceous; integument shiny, with evident microsculpture and pubescence.

***Head*** robust, not hypertrophic, narrower than pronotum; excess setae absent. Labium toothless, mentum articulated. Antennae robust, moniliform, short, hardly exceeding the base of the pronotum when stretched backwards. Fronto-clypeal furrow indistinct; subrectilinear anterior margin of epistome.

***Pronotum*** transverse (max. width / max. length ratio = 1.25), with maximum width at the base of the anterior fourth, and basal border remarkably narrower than anterior border; sides distinctly and regularly arcuate anteriorly, sinuate before basal angles. Anterior angles rounded, not prominent; posterior angles obtuse and rounded. Disc convex, with very sparse pubescence of medium length; median groove very shallow, hardly evident. Marginal groove wide and flat, slightly enlarged near the base; anterior marginal setae placed inside the marginal groove, almost on the anterior fourth; basal setae at posterior angles.

***Legs*** short and stout, with metatrochanters short and stout and metafemora non dentate. Two protarsomeres dilated and without adhesive phanerae in male.

***Elytra*** subrectangular (max. length / max. width ratio = 1.55), with maximum width at the base of the posterior third, subtruncate but not emarginated before apex. Disc slightly convex, without longitudinal groove; integument shiny, with evident microsculpture and very sparse and upright pubescence of medium length, longitudinally aligned. Humeri well marked but rounded; post-humeral margin denticulate, with distinct crenulation up to the apical third; elytral apices separately rounded. Marginal groove wide and evident almost up to the 9^th^ pore of the umbilicate series.

***Chaetotaxy***: large and foveate basal umbilicate pore. Umbilicate series with the first three pores of the humeral group very closed to each other and equidistant; 4^th^ pore farther and placed at the end of the basal third of the elytron; 5^th^ pore placed at the base the apical third of the elytron; 5^th^ and 6^th^ ones spaced from each other equidistant with 6^th^ and 7^th^; 5^th^, 7^th^ and 8^th^ displaced onto the disc; 7^th^ and 8^th^ spaced from each other as 8^th^ and 9^th^. Two discal setae, the first placed at the level of the 3^rd^ pore of the umbilicate series, the second one placed just before the 8^th^ pore.

***Aedeagus*** (Fig. 9) small, median lobe short, stout, subrectilinear, with basal bulb extremely reduced; ventral margin gently bisinuated; apical blade evident. Endophallus without an evident lamella copulatrix. Left paramere elongated, reaching the aedeagal distal fourth and bearing only one seta; right paramere lost during the preparation of the specimen.

##### Etymology.

The name arises from the type locality Ophthalmia Range in the Pilbara region.

##### Distribution.

*Gregorydytes ophthalmianus* sp. nov. is known so far only from the type locality Ophthalmia Range, 25 km W of Newman, Pilbara, WA.

#### Pilbaraphanus gen. nov.

Taxon classificationAnimaliaColeopteraCarabidae

09074B2F-2DE8-5F68-8FC4-283571657045

http://zoobank.org/F300FB18-16FE-468B-B9BA-CC5C0926CFDF

Figs 10–14

##### Type species.

*Pilbaraphanus chichesterianus* sp. nov.

##### Diagnosis.

Genus characterised by very long and sharp metatrochanters; dentate metafemora; elytra not reduced at tip; elytral disc bearing three setae and longitudinal grooves absent; 9^th^ pore of the umbilicate series in the normal position (placed after the 8^th^); aedeagus with median lobe not very curved, size of basal bulb normal and parameres bearing two apical setae. Labial tooth present and smooth. Differs from *Magnanillus* Baehr, 2017 by the absence of longitudinal elytral grooves.

##### Description.

Species of medium size (TL mm 1.37–2.18) and anophthalmous. Integument depigmented but well sclerified and covered with sparse pubescence.

***Head*** from normal to large size, slightly narrower than pronotum; mandibles short and simple, without hyperplasias. Maxillary palpi ovoidal and swollen. Labium transverse and articulated; mentum not fused with submentum. Labial tooth present and smooth. Antennae moniliform, without particular features.

***Pronotum*** subquadrate, with sides not or only slightly sinuate at the basal third, smooth or denticulated before basal angles. Basal angles right, sharp, not rounded; basal border as wide as, or only slightly narrower, than the anterior border; presence of two marginal setae, the posterior one placed just before basal angles.

***Elytra*** subrectangular and elongated, separately rounded and depressed, not truncate and not emarginated apically, with longitudinal groove absent. Elytral striae absent (except sutural stria). Lateral margin starting from humeral area, distinctly crenulate up to at least half-length. Scutellar pore present, large and umbilicate; umbilicate series of type B (sensu Jeannel 1963; Giachino and Vailati 2011); disc bearing three discal setae.

***Legs*** relatively short and stumpy. Unarmed profemora; posterior edge of metafemora dentate, metatrochanters very long and sharp, two dilated protarsomeres in the male.

***Aedeagus*** relatively large, median lobe long and not very curved, with basal bulb of normal size. Parameres long and bearing two apical setae. Endophallus with poorly sclerified phanerae.

##### Etymology.

The genus name combines the Pilbara name with the suffix -*phanus* to recognise the genus *Illaphanus*. Gender masculine.

##### Species included.

Only two species currently belong to this genus:

*Pilbaraphanus chichesterianus* sp. nov.

*Pilbaraphanus bilybarianus* sp. nov.

**Figures 10–14.**
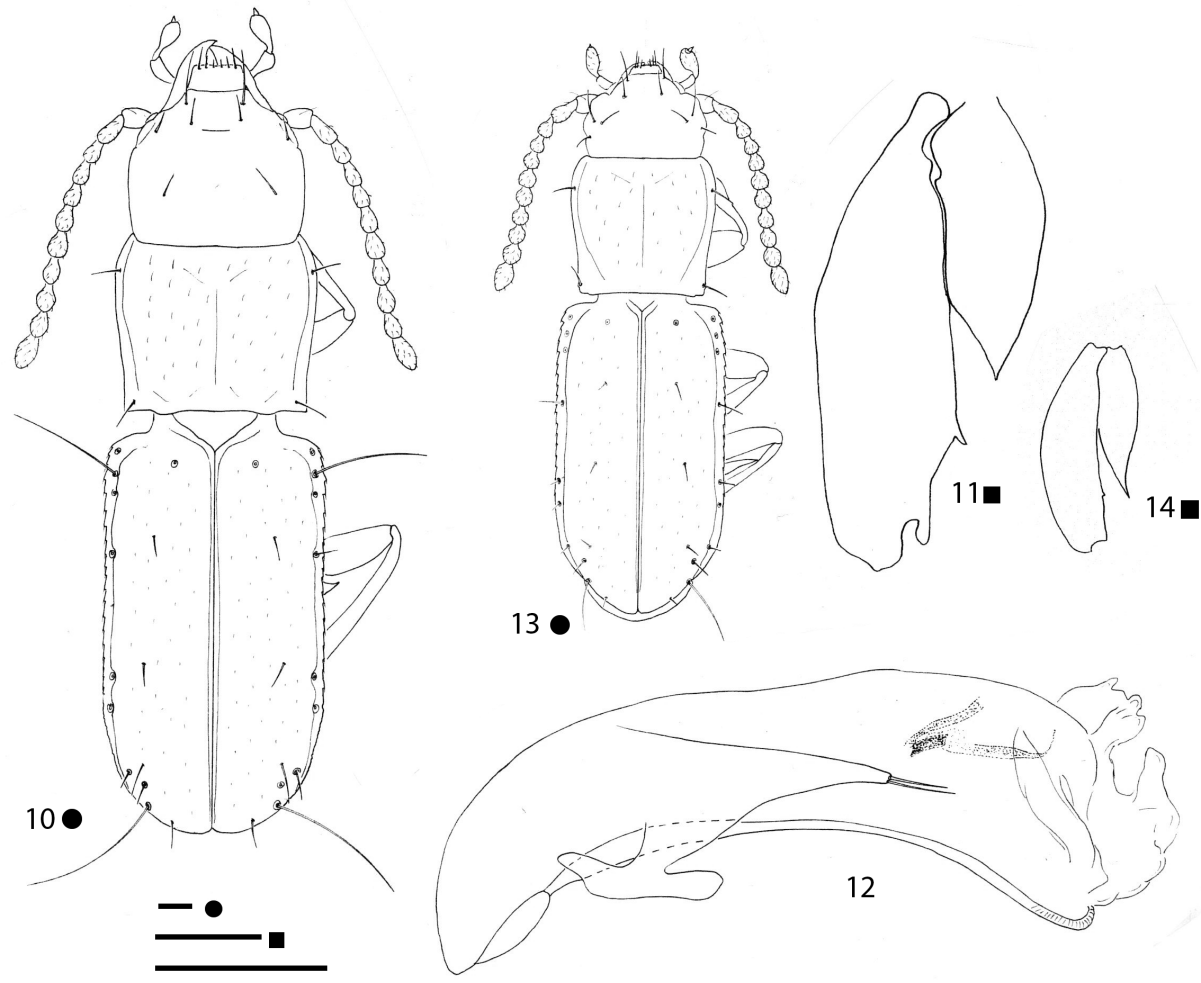
*Pilbaraphanus* gen. et spp. nov., habitus (**10, 13**) right metafemur and metatrochanter in ventral view (**11, 14**) aedeagus in lateral view (**12**) **10–12***P. chichesterianus* sp. nov., HT ♂ **13, 14***P. bilybarianus* sp. nov. HT ♀. Scale bars: 0.1 mm.

### Key to the species of *Pilbaraphanus*

**Table:** 

1	Metatrochanters long and sharp, shorter than femoral tooth. Pronotum more transverse	***P. chichesterianus* sp. nov.**
–	Metatrochanters very long and sharp, as long as femoral tooth. Pronotum less transverse	***P. bilybarianus* sp. nov.**

#### Pilbaraphanus chichesterianus sp. nov.

Taxon classificationAnimaliaColeopteraCarabidae

C5DB148C-EEA2-5429-9DEC-5B5DD39C604E

http://zoobank.org/8D765714-941C-4E02-942E-9C38A55F066F

Figs 10–12

##### Type locality.

WA, Pilbara, 100 km E of Port Hedland, Chichester Ranges, Quarry 8, 21°59'43.58"S, 119°0'38"E.

##### Type series.

HT ♂, WA, Pilbara, 100 km E of Port Hedland, Chichester Ranges, Quarry 8, 21°59'43.58"S, 119°0'38"E, (WGS84), P. Bell, 3 Jun. 2008, Stygo Net Haul. (Q8-EXR1622R-LN1735), Western Australian Museum Entomology Reg. no. 82635 (WAM).

##### Differential diagnosis.

*Pilbaraphanus chichesterianus* sp. nov. and *P. bilybarianus* sp. nov. are closely related and share the characters indicated in the genus diagnosis. *P. chichesterianus* sp. nov. differs from *P. bilybarianus* sp. nov. by its bigger body size, shorter metatrochanters, and more transverse pronotum.

##### Description of the HT ♂.

TL mm 2.18. ***Body*** elongated and depigmented, yellow-testaceous; integument shiny, with evident microsculpture and short pubescence.

***Head*** robust, hypertrophic, slightly narrower than pronotum; without excess setae. Labium with a smooth tooth, mentum articulated. Antennae robust, moniliform, short, not reaching the base of the pronotum when stretched backwards. Fronto-clypeal furrow indistinct; anterior margin of the epistome subrectilinear.

***Pronotum*** subsquare (max. width / max. length ratio = 1.12), with maximum width at the base of the anterior fifth, and with basal border remarkably wider than anterior border; sides slightly and irregularly arcuate in the anterior part, subrectilinear before basal angles. Anterior angles acute, prominent; posterior angles squared and not rounded. Disc convex, with very sparse pubescence of medium length; median groove very shallow, hardly evident. Marginal groove wide and flat, slightly enlarged near the base; anterior marginal setae placed inside the marginal groove, almost on the anterior fifth; basal setae placed inside the disk and before posterior angles.

***Legs*** long and slender, with metatrochanters long and acuminate, but not curved, and metafemora dentate; metatrochanters (Fig. 11) shorter than femoral tooth. Two protarsomeres dilated and without adhesive phanerae in males. Left pro-, left and right meso-, and left metalegs missing in the HT ♂.

***Elytra*** perfectly subrectangular (max. length / max. width ratio = 1.80), not truncate and not emarginated before apex. Disc convex, longitudinal grooves absent; integument shiny, with evident microsculpture, and very short, sparse and upright, pubescence longitudinally aligned. Humeri well marked, gently rounded; post-humeral margin denticulate, with distinct crenulation up to the apical third; elytral apices separately rounded. Marginal groove wide and evident almost up to the 8^th^ pore of the umbilicate series.

***Chaetotaxy***: scutellar pore large and foveate. Umbilicate series with the first three pores of the humeral group very closed to each other and equidistant; 4^th^ pore farther and placed at the end of the basal third of the elytron; 5^th^ pore placed before the base of the apical third of the elytron; 5^th^ and 6^th^ ones spaced from each other half the distance from 6^th^ and 7^th^; 7^th^ and 8^th^ displaced onto the disc; 7^th^ and 8^th^ spaced to each other as the 8^th^ and 9^th^. Three discal setae, the first placed before the 4^th^ pore of the umbilicate series, the second and third ones placed respectively just before the 5^th^ and 8^th^ umbilicate pores.

***Aedeagus*** (Fig. 12) large, median lobe long, stout, subrectilinear, with basal bulb extremely reduced; ventral margin gently curved; apical blade evident but short. Endophallus without an evident lamella copulatrix, but with two crossing apical slightly sclerified stripes. Left paramere elongated, reaching the aedeagal distal third and bearing two setae; right paramere lost during the preparation of the specimen.

##### Etymology.

The species name comes from the Chichester Range, where the type locality (Quarry 8) is located.

##### Distribution.

*Pilbaraphanus chichesterianus* sp. nov. is known so far only from the type locality Quarry 8 in the Chichester Range, 100 km E of Port Hedland, Pilbara, WA.

#### Pilbaraphanus bilybarianus sp. nov.

Taxon classificationAnimaliaColeopteraCarabidae

7FB9AD69-B2B1-568F-B7B0-C8D494841646

http://zoobank.org/EFE99D5F-1296-44D9-AA9F-213640C5178A

Figs 13–14

##### Type locality.

WA, Pilbara, 60 km N of Tom Price, Solomon Mining Area, Kings deposit, 22°09'31.44"S, 117°51'50.9E.

##### Type series.

HT ♀, WA, Pilbara, 60 km N of Tom Price, Solomon Mining Area, Kings deposit, 22°09'31.44"S, 117°51'50.9E (WGS84), P. Bell, E.S. Volschenk, 24.Jan. 2010; Trog. net scrape (FMG005_SM0347_10:7877 Western Australian Museum Entomology Reg. no. 82607 (WAM).

##### Differential diagnosis.

*Pilbaraphanus bilybarianus* sp. nov. and *P. chichesterianus* sp. nov. are closely related and share the characters described in the genus diagnosis. *P. bilybarianus* sp. nov. differs from *P. chichesterianus* sp. nov. by its smaller body size, longer metatrochanters, and less transverse pronotum.

##### Description of the HT ♀.

TL mm 1.37. ***Body*** elongated and depigmented, yellow; integument shiny, with evident microsculpture and short pubescence.

***Head*** robust, hypertrophic, narrower than pronotum; excess setae absent. Labium with smooth tooth, mentum articulated. Antennae robust, moniliform, short, reaching the base of the pronotum when stretched backwards. Fronto-clypeal furrow indistinct; anterior margin of the epistome subrectilinear.

***Pronotum*** subsquare (max. width / max. length ratio = 1.03), with the maximum width at the base of the anterior fourth, and with basal border remarkably wider than anterior border; sides poorly and not regularly arcuate in the anterior part, gently sinuate in the basal half and slightly dentate before basal angles. Anterior angles obtuse, prominent; posterior angles squared, gently rounded. Disc convex, with very sparse pubescence of medium length; median groove very shallow, hardly evident. Marginal groove wide and flat, enlarged near the base; anterior marginal setae placed inside the marginal groove, almost on the anterior fourth; basal setae not placed inside on the disk, but before the posterior angles.

***Legs*** long and slender, with metatrochanters long and acuminate, gently curved and metafemora dentate; metatrochanters (Fig. 14) as long as femoral tooth. All left legs missing in the HT ♀.

***Elytra*** perfectly subrectangular (max. length / max. width ratio = 1.91), not truncated, only slightly emarginated before the apex. Disc convex, longitudinal grooves absent; integument shiny with evident microsculpture, and very short, sparse, upright pubescence not longitudinally aligned. Humeri well marked, gently rounded; post-humeral margin denticulate, with distinct crenulation up to the apical third; elytral apices separately rounded. Marginal groove wide and evident almost up to the 9^th^ pore of the umbilicate series.

***Chaetotaxy***: scutellar pore large and foveate. Umbilicate series with the first three pores of the humeral group very closed to each other and equidistant; 4^th^ pore farther and placed at the end of the basal third of the elytron; 5^th^ pore placed before the base of the apical third of the elytron; 5^th^ and 6^th^ ones spaced from each other as half distance from 6^th^ and 7^th^; 7^th^ and 8^th^ displaced onto the disc; 7^th^ and 8^th^ spaced from each other as the 8^th^ and 9^th^. Three discal setae, the first placed before the 4^th^ pore of the umbilicate series, the second one placed just before the 5^th^, and the third one placed at the level of the 7^th^ pore of the umbilicate series.

**Male.** Unknown.

##### Etymology.

The species name derives from *Bilybara*, aboriginal name that refers to the Pilbara region.

##### Distribution.

*Pilbaraphanus bilybarianus* sp. nov. is known only from the type locality (Kings deposit, which is part of the Solomon Mining Area), 60 km N of Tom Price, Pilbara, WA.

#### Magnanillus

Taxon classificationAnimaliaColeopteraCarabidae

Baehr, 2017

9B5AB937-0230-5E30-9E7B-7239586E527D

Figs 15–17 , 18–20 , 21–23 , 24–26 , 27–29

Macranillus Baehr & Main, 2016: 61.
Magnanillus Baehr, 2017: 237.

##### Type species.

*Macranillus bennetti* Baehr & Main, 2016: 61.

##### Diagnosis.

This genus is characterised by: metatrochanters very long and sharp; metafemora dentate; elytra not reduced at tip; elytral disc with longitudinal grooves and bearing three setae; 9^th^ pore of the umbilicate series in normal position (placed after the 8^th^ one); aedeagus with median lobe gently curved, size of basal bulb normal and parameres bearing two apical setae. Labial tooth lacking. Differs from *Pilbaraphanus* by the presence of a longitudinal elytral grooves and the lack of labial tooth.

##### Note.

The description of this genus by Baehr and Main (2016) (sub *Macranillus*), although very accurate, does not consider some peculiar characters present in species belonging to *Macranillus*, in particular: the elytral chaetotaxy, the absence of labial tooth, the presence of teeth on the posterior margin of metafemora, and the elongated and sharpened shape of the metatrochanters. The presence of the last two characters in *Magnanillus* was confirmed (M. Baehr pers. comm. 2018). The genus is redescribed below.

##### Redescription.

Species of medium-large size (TL mm 1.44–2.51) and anophthalmous. Integument depigmented but well sclerified and covered with sparse pubescence.

***Head*** size normal to large, well narrower, or just narrower, than pronotum; mandibles short and simple, without hyperplasias. Maxillary palps ovoidal, swollen. Labium transverse, articulated; mentum not fused with submentum. Labial tooth absent. Antennae moniliform, without particular features.

***Pronotum*** subquadrate, with sides not, or only slightly, sinuate at the basal third, smooth or denticulated before basal angles. Basal angles right or obtuse, sharp, not rounded; base as wide as, or only slightly narrower, than anterior margin; presence of two marginal setae, the posterior one placed just before the basal angles.

***Elytra*** subrectangular and elongated, separately rounded, not truncated and not emarginated apically; depressed, with a longitudinal groove. Elytral striae absent (except sutural stria). Lateral margin, starting from the humeral area and distinctly crenulate up to at least half-length. Scutellar pore present, large and umbilicate; umbilicate series of type B (sensu Jeannel 1963; Giachino and Vailati 2011); disc bearing three discal setae.

***Legs*** relatively short and stumpy. Profemora unarmed; metafemora dentate on the posterior edge, metatrochanters very long and sharp, two dilated protarsomeres in the male.

***Aedeagus*** relatively large, median lobe long and slightly curved, with basal bulb of normal size. Parameres long, each bearing two apical setae. Endophallus with poorly sclerified phanerae.

##### Species included.

Currently ten species belong to this genus:

*Magnanillus firetailianus* sp. nov.

*Magnanillus quartermaini* (Baehr & Main, 2016)

*Magnanillus sabae* sp. nov.

*Magnanillus salomonis* sp. nov.

*Magnanillus regalis* sp. nov.

*Magnanillus serenitatis* sp. nov.

*Magnanillus bennetti* (Baehr & Main, 2016)

*Magnanillus pearsoni* (Baehr & Main, 2016)

*Magnanillus maini* (Baehr & Main, 2016)

*Magnanillus magnus* (Baehr & Main, 2016)

##### Note.

The key to species of *Magnanillus* Baehr, 2017 lack clear information on important diagnostic characters such as metafemora and metatrochanter morphology (see genus redescription). For the identification of the described species of *Magnanillus* refer to Baehr and Main (2016).

#### Magnanillus firetailianus sp. nov.

Taxon classificationAnimaliaColeopteraCarabidae

CE565E4F-410C-52CB-8199-169747A80E79

http://zoobank.org/9F0483A9-3831-45AC-936D-D52CD9444C51

Figs 15–17

##### Type locality.

WA, Pilbara, 50 km N of Tom Price, Solomon Mining Area, Firetail deposit, 22°07'25.3"S, 117°53'59.2"E.

##### Type series.

HT ♂, WA, Pilbara, 50 km N of Tom Price, Solomon Mining Area, Firetail Mine (drill hole FT0430), 22°07'25.3"S, 117°53'59.2"E (WGS84); G. Pearson, D. Main, 20 May 2010; Trog. Trap., Western Australian Museum Entomology Reg. no. 82657 (WAM). PTT: 6 ♂♂ 3 ♀♀ (+ remains of 1 ♂ 2 ♀♀), WA, Pilbara, 50 km N of Tom Price, Solomon Mining Area, Firetail Mine (drill hole FT0430), 22°07'25.3"S, 117°53'59.2"E (WGS84), G. Pearson, D. Main, 20 May 2010, Trog. Trap., Western Australian Museum Entomology Reg. no. 82658–82662 (WAM, CGi); 1 ♀, WA, Pilbara, 50 km N of Tom Price, Solomon Mining Area, Firetail Mine (drill hole FT1455), 22°08'53.1"S, 117°55'37.8"E (WGS84) G. Pearson, D. Main, 19 May 2010, Trog. Trap., Western Australian Museum Entomology Reg. no. 82663 (WAM).

##### Differential dagnosis.

*Magnanillus firetailianus* sp. nov. is easily distinguishable from the other species of this genus (except *M. quartermaini* (Baehr & Main, 2016)) by its pronotum with basal border narrower than anterior border. It also differs from *M. sabae* sp. nov., *M. salomonis* sp. nov. and *M. regalis* n. sp by much longer metatrochaters, extended over the position of the femoral tooth. *M. firetailianus* sp. nov. differs from *M. serenitatis* sp. nov. by its bigger size, while it differs from *M. quartermaini* by a less transverse pronotum.

##### Description.

TL mm 1.44–1.46 ♂♂, 1.48–1.50 ♀♀. ***Body*** elongate, depigmented, yellow; integument shiny, with evident microsculpture, and short pubescence.

***Head*** robust, hypertrophic, slightly narrower than pronotum; without excess setae. Labium without tooth, mentum articulated. Antennae robust, moniliform, short, reaching the base of the pronotum when stretched backwards. Fronto-clypeal furrow indistinct; subrectilinear anterior margin of epistome.

***Pronotum*** sub-rectangular (max. width / max. length ratio = 1.14), with maximum width at the base of the anterior fifth, and with basal border remarkably narrower than anterior border; sides slightly and irregularly arcuate in the anterior part, subrectilinear at the basal half, gently sinuate and slightly dentate before basal angles. Anterior angles obtuse, slightly prominent; posterior angles squared, acute. Disc convex, with very sparse pubescence of medium length; median groove very shallow, hardly evident. Marginal groove wide and flat, enlarged near the base; anterior marginal setae placed inside the marginal groove, almost on the anterior fifth; basal setae not inside the disk, but placed before posterior angles.

***Legs*** long and slender, with metatrochanters long and acuminate, gently curved and metafemora dentate; metatrochanters (Fig. 16) slightly longer than femoral tooth. Two dilated protarsomeres in males.

***Elytra*** perfectly subrectangular (max. length / max. width ratio = 1.80), not truncated and not emarginated before the apex. Disc convex, with longitudinal grooves; integument shiny, with evident microsculpture, very short, sparse, and upright, pubescence, not longitudinally aligned. Humeri well marked, gently rounded; post-humeral margin denticulate, with distinct crenulation up to the apical third; elytral apices separately rounded. Marginal groove wide and evident almost up to the 8^th^ pore of the umbilicate series.

***Chaetotaxy***: scutellar pore large and foveate. Umbilicate series with the first three pores of the humeral group very closed to each other and equidistant; 4^th^ pore farther and placed at the end of the basal third of the elytron; 5^th^ pore placed before the base of the apical third of the elytra; 5^th^ and 6^th^ ones spaced out ca. half of the distance between 6^th^ and 7^th^; 7^th^ and 8^th^ displaced onto the disc; 7^th^ and 8^th^ spaced from each other as the 8^th^ and 9^th^. Three discal setae, the first placed before the 4^th^ pore of the umbilicate series, second one placed just before the 5^th^, the third one placed at the 7^th^ pore.

***Aedeagus*** (Fig. 17) large, median lobe long, stout, gently curved, with basal bulb small, but tight and evident; ventral margin gently curved from basal bulb to apex; apical blade evident but short. Endophallus without an evident lamella copulatrix, but with two small, apical, Y-shaped, crossing and slightly sclerified stripes. Left paramere elongated, not reaching the distal third and bearing two setae; right paramere shorter and bearing two apical setae.

##### Etymology.

The name comes from the type locality in the Solomon Mining Area, the Firetail deposit, in the Pilbara region.

##### Distribution.

*Magnanillus firetailianus* sp. nov. is known only from the type locality (Firetail deposit) 50 km N of Tom Price, Pilbara, WA.

**Figures 15–17.**
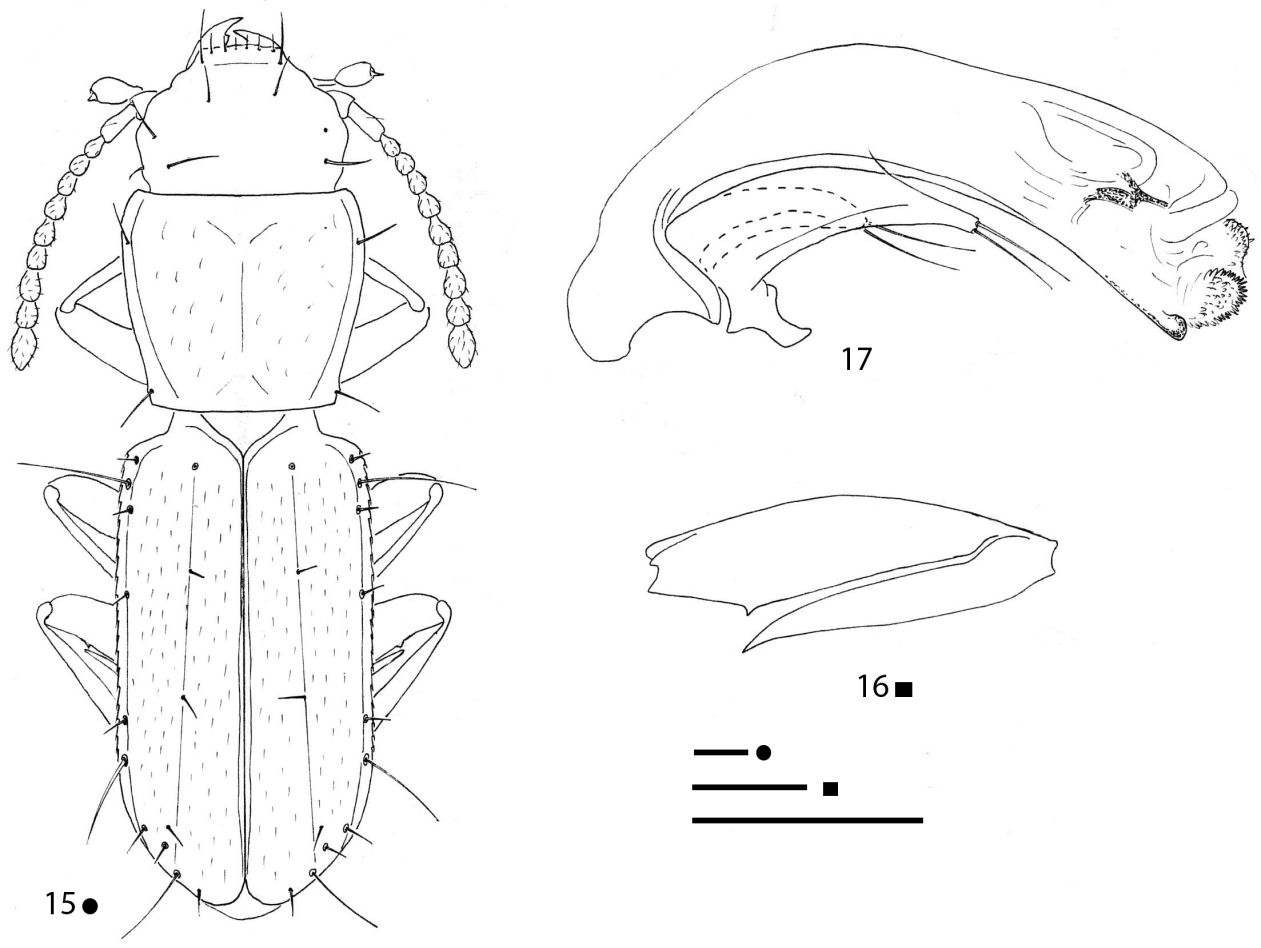
*Magnanillus firetalianus* sp. nov., HT ♂ **15** habitus **16** right metafemur and metatrochanter in ventral view **17** aedeagus in lateral view. Scale bars: 0.1 mm.

#### Magnanillus sabae sp. nov.

Taxon classificationAnimaliaColeopteraCarabidae

1CE389B1-CDDC-5354-9EED-6975F16CBA17

http://zoobank.org/62280A8E-1510-4C97-9D71-BA0DE7F6B321

Figs 18–20

##### Type locality.

WA, Pilbara, 60 km NW of Tom Price, Solomon Mining Area, Serenity deposit, 22°8'4.02"S, 117°32'3.444E.

##### Type series.

HT ♂, WA, Pilbara, 60 km NW of Tom Price, Solomon Mining Area, Serenity mine, (drill hole SMD0057), 22°8'4.02"S, 117°32'3.444E (WGS84), N. Coen, S. Catomore, 19 Apr. 2011, Trog. net scrape, Western Australian Museum Entomology Reg. no. 82651 (WAM). PTT: 1 ♀, WA, Pilbara, 60 km NW of Tom Price, Solomon Mining Area, Serenity mine, (drill hole SR0126), 22°11'22.70"S, 117°32'28.68E (WGS84), N. Coen, S. Catomore, 30 May 2011, Trog. net scrape, Western Australian Museum Entomology Reg. no. 82652 (WAM); 1 ♀, WA, Pilbara, 60 km NW of Tom Price, Solomon Mining Area, Sheila Valley, (drill hole SV0577), 22°13'1.236"S, 117°40'55.2E (WGS84), N. Coen, S. Catomore, 18 Apr. 2011, Trog. Net scrape, Western Australian Museum Entomology Reg. no. 82640 (WAM);1 ♂, WA, Pilbara, 60 km NW of Tom Price, Solomon Mining Area, Sheila Valley, (drill hole SV0443), 22°14'45.59"S, 117°38'46.71E (WGS84), S. Eberhard, S. Catomore, 05 Oct. 2010, Stygo net haul., Western Australian Museum Entomology Reg. no. 82615 (CGi); 1 ♀, WA, Pilbara, 50 km N of Tom Price, Solomon Mining Area, Zion mine, (drill hole SM0282) 22°12'00.8"S, 117°57'16.71E (WGS84), E.S. Volschenk, N. Krawczyk, 01.Mar. 2010, Trog. Trap, Western Australian Museum Entomology Reg. no. 82610 (WAM).

##### Differential diagnosis.

*Magnanillus. sabae* sp. nov. can be easily distinguished from *M. firetailianus* sp. nov. by its base of pronotum ca. as large as the pronotal apex. It can be distinguished from *M. salomonis* sp. nov., *M. regalis* sp. nov., and *M. serenitatis* sp. nov. by its short metatrochanters not reaching the femoral tooth. It differs from *M. pearsoni* (Baehr & Main, 2016) by longer elytra and more transverse pronotum.

##### Description.

TL mm 2.42–2.46 ♂♂, 2.48–2.51 ♀♀. ***Body*** elongated, depigmented, testaceous; integument shiny, with evident microsculpture and very short pubescence.

***Head*** robust, narrower than pronotum, excess setae absent. Labium without tooth, mentum articulated. Antennae robust, moniliform, short, reaching the base of the pronotum when stretched backwards. Fronto-clypeal furrow indistinct; anterior margin of the epistome subrectilinear.

***Pronotum*** sub-squared (max. width / max. length ratio = 1.14), with maximum width at the base of the anterior fourth, and basal border slightly wider than anterior border; sides slightly and irregularly arcuate in the anterior part, subrectilinear in the basal half, not sinuate and slightly denticulate before basal angles. Anterior angles obtuse, slightly prominent; posterior angles obtuse, blunted. Disc convex, with very short and sparse pubescence; median groove very shallow, hardly evident. Marginal groove wide and flat, enlarged near the base; anterior marginal setae placed inside the marginal groove, almost on the anterior fifth; basal setae not inside on the disk, but placed before the posterior angles.

***Legs*** long and slender, with metatrochanters long, acuminate and subrectilinear and metafemora dentate; metatrochanters (Fig. 19) shorter than femoral tooth. Two dilated protarsomeres in males.

***Elytra*** subrectangular, relatively short (max. length / max. width ratio = 1.63), not truncated and very slightly emarginated before apex. Disc convex, with longitudinal grooves; very short, sparse, and upright pubescence not longitudinally aligned. Humeri well marked, obtuse; post-humeral margin denticulate, with a distinct crenulation down to the apical third; elytral apices separately rounded. Marginal groove wide and evident almost up to the 7^th^ pore of the umbilicate series.

***Chaetotaxy***: scutellar pore large and foveate. Umbilicate series with the first three pores of the humeral group very closed to each other and equidistant; 4^th^ pore farther and placed at the end of the basal third of the elytron; 5^th^ pore placed before the base of the apical third of the elytron; 5^th^ and 6^th^ ones spaced out ca. 1/3 of the distance from 6^th^ and 7^th^; 7^th^ and 8^th^ displaced onto the disc; 7^th^ and 8^th^ spaced from each other as the 8^th^ and 9^th^. Three discal setae, first placed before the 4^th^ pore of the umbilicate series, second one placed just before the 5^th^, third one placed before the 7^th^ pore.

***Aedeagus*** (Fig. 20) large, median lobe long, stout, gently curved, with basal bulb small but evident; ventral margin gently curved from basal bulb to apex; apical blade poorly evident, very short. Endophallus without an evident lamella copulatrix, but with two small, apical, subparallel, and slightly sclerified stripes. Left paramere elongate, not reaching the aedeagal distal third and bearing two setae; right paramere shorter and bearing two apical setae.

##### Etymology.

The name is to remind one of the Solomon mining area and the different deposits where this species occurs, and it originates from the mythological Queen of Sheba (in Latin Sheba = *Saba*). According to tradition, the Queen of Sheba visited the Kingdom of Solomon with valuable gifts for its king.

##### Distribution.

*Magnanillus sabae* sp. nov. is known from different deposits of the Solomon Mining Area, 50–60 km N/NW of Tom Price, Pilbara, WA.

**Figures 18–20.**
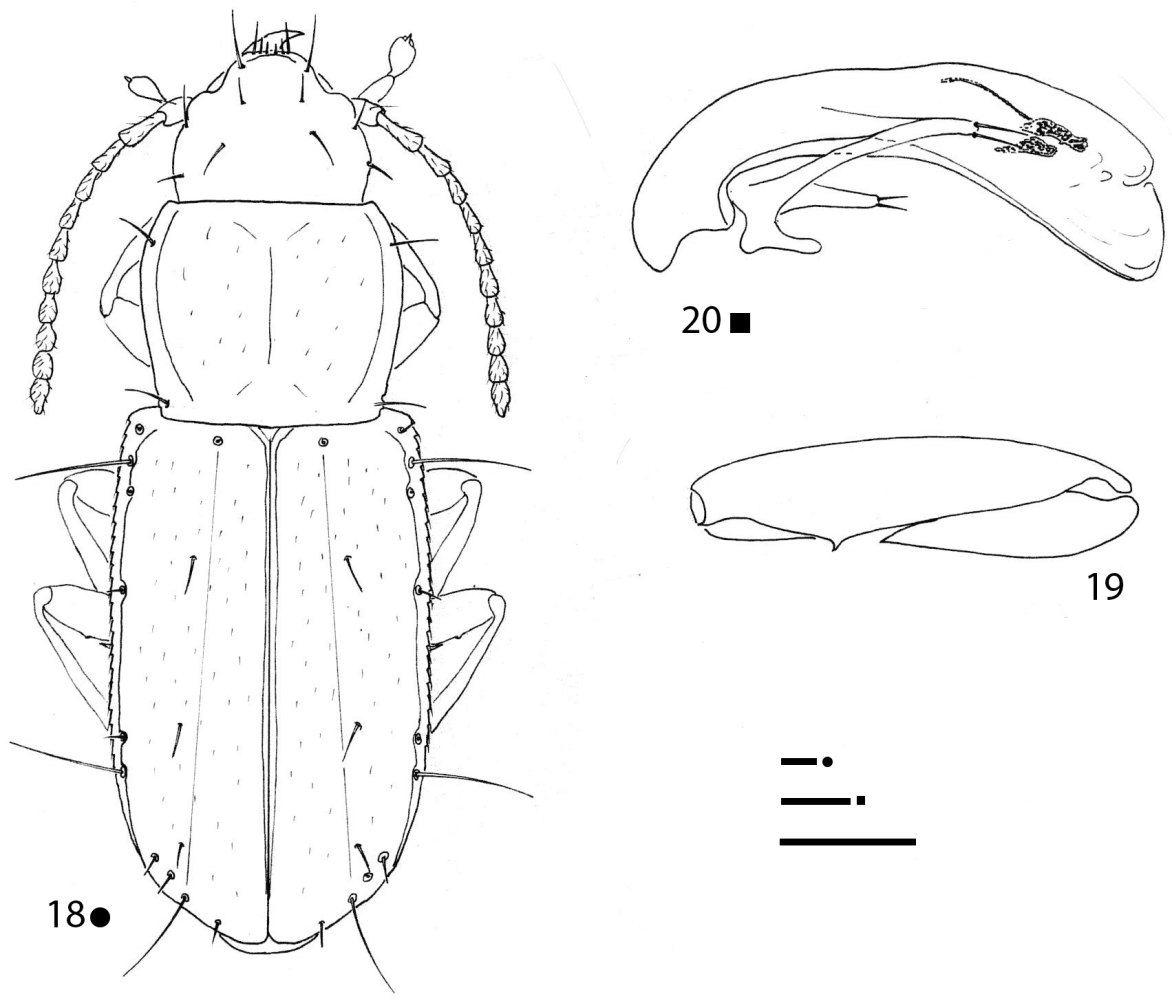
*Magnanillus sabae* sp. nov., HT ♂ **18** habitus **19** right metafemur and metatrochanter in ventral view **20** aedeagus in lateral view. Scale bars: 0.1 mm.

#### Magnanillus salomonis sp. nov.

Taxon classificationAnimaliaColeopteraCarabidae

49F878A1-A488-5017-B069-D32DBB88145A

http://zoobank.org/F3E787C1-604D-4F6C-8E5A-262E79AC2715

Figs 21–23

##### Type locality.

WA, Pilbara, 50 km N of Tom Price, Solomon Mining Area, Kings deposit, 22°12'00.8"S, 117°57'16.71E.

##### Type series.

HT ♂, WA, Pilbara, 50 km N of Tom Price, Solomon Mining Area, Kings mine, 22°12'00.8"S, 117°57'16.71E (WGS84), E.S. Volschenk, N. Krawczyk, 01.March 2010, Trog. trap (FMG005_SM0282_10:8313), WA Museum Entomology Reg. no. 82609 (WAM). PTT: 1 ♂ (remains) 6 ♀♀ and 3 spec. (♂♀?),WA, Pilbara, 60 km NW of Tom Price, Solomon Mining Area, Serenity mine, 22°11'9.24"S, 117°32'49.524E (WGS84), N. Coen, S. Catomore, 20.04.2011, Stygo net haul (FMG008_SOM0039_11:0879 Western Australian Museum Entomology Reg. no. 82641–82650 (WAM, CGi); 1 ♀, WA, Pilbara, 50 km N of Tom Price, Solomon Mining Area, Firetail Mine, 22°07'32.2"S, 117°29'34.6"E (WGS84), G. Pearson and D. Main, 13"Sept 2010, Stygo. net haul (HPRC0211) Western Australian Museum Entomology Reg. no. 82664 (WAM); 1 ♂ 1 ♀ (remains) 1 spec. (remains), WA, Pilbara, 50 km N of Tom Price, Solomon Mining Area, Firetail Mine, 22°06'44.2"S, 117°53'28.8"E (WGS84), G. Pearson and D. Main, 3 Mar. 2010, Trog. trap (FT0541) Western Australian Museum Entomology Reg. no. 82654–82656 (WAM); 1 ♀ (remains), WA, Pilbara, 60 km NW of Tom Price, Solomon Mining Area, Sheila Valley, 22°12'00.39"S, 117°42'14.52E (WGS84), M. Weerheim, S. Catomore, 9 Dec. 2010, Trog. net scrape, (FMG006_SV0267_10:0955), Western Australian Museum Entomology Reg. no. 82613 (WAM); 1 spec. (remains), WA, Pilbara, 60 km NW of Tom Price, Solomon Mining Area, Sheila Valley, 22°14'45.59"S, 117°38'46.71E (WGS84), S. Eberhard, S. Catomore, 05 Oct. 2010, Stygo net haul. (FMG006_SV0443_10:0490), Western Australian Museum Entomology Reg. no. 82614 (WAM).

##### Differential diagnosis.

*Magnanillus salomonis* sp. nov. is easily distinguishable from *M. firetailianus* sp. nov. by its protonum with basal border ca. as wide as the anterior border. It can be distinguished from *M. sabae* sp. nov. by its longer metatrochanters, reaching the femoral tooth. It can be distinguished from *M. serenitatis* sp. nov. by its shorter metatrochanters, not overreaching the femoral tooth. It can be distinguished from *M. regalis* sp. nov. by its more transverse pronotum, and the clearly curved apex of the metatrochanters. It can be distinguished from *M. benneti* (Baehr & Main, 2016), *M. pearsoni* (Baehr & Main, 2016), *M. maini* (Baehr & Main, 2016), *M. magnus* (Baehr & Main, 2016) and *M. quartermaini* (Baehr & Main, 2016) by its more transverse pronotum.

##### Description.

TL mm 2.20–2.22 ♂♂, 2.25–2.28 ♀♀. ***Body*** elongated, depigmented, testaceous; integument shiny with evident microsculpture and very short pubescence.

***Head*** relatively small, narrower than pronotum; excess setae absent. Labium without tooth, mentum articulated. Antennae robust, submoniliform, short, reaching the base of the pronotum when stretched backwards. Fronto-clypeal furrow indistinct; anterior margin of the epistome subrectilinear.

***Pronotum*** transverse (max. width / max. length ratio = 1.16), with maximum width at the base of the anterior fourth, and basal border wider than anterior border; sides slightly and irregularly arcuate in anterior part, subrectilinear in the basal half, not sinuate and denticulate before basal angles. Anterior angles obtuse, slightly prominent; posterior angles right, acute. Disc convex, with short and very sparse pubescence; median groove very shallow, hardly evident. Marginal groove wide and flat, enlarged near the base; anterior marginal setae placed inside the marginal groove, almost on the anterior fourth; basal setae slightly located internally on the disk and placed before the posterior angles.

***Legs*** long and slender, with metatrochanters long, acuminate, and gently curved and metafemora dentate; metatrochanters (Fig. 22) as long as the femoral tooth. Anterior legs missing in all male specimens.

***Elytra*** subrectangular, relatively short (max. length / max. width ratio = 1.69), not truncate and not emarginated before the apex. Disc convex, with longitudinal grooves; integument shiny with evident microsculpture, very short, sparse, and upright, pubescence, not longitudinally aligned. Humeri well marked, obtuse; post-humeral margin denticulate, with distinct crenulations up to the base of the apical third; elytral apices separately rounded. Marginal groove wide and evident almost up to the 8^th^ pore of the umbilicate series.

***Chaetotaxy***: scutellar pore large and foveate. Umbilicate series with the first three pores of the humeral group very closed to each other and equidistant; 4^th^ pore farther and placed at the end of the basal third of the elytron; 5^th^ pore placed at the base of the apical third of the elytron; 5^th^ and 6^th^ ones spaced out ca. half of the distance between 6^th^ and 7^th^; 7^th^ and 8^th^ displaced onto the disc; 7^th^ and 8^th^ spaced from each other as the 8^th^ and the 9^th^. Three discal setae, first placed before the 4^th^ pore of the umbilicate series, second one placed in the middle of the elytron, the third one placed before the 7^th^ pore.

***Aedeagus*** (Fig. 23) large, median lobe long, stout, gently curved, with basal bulb small but tight and evident; ventral margin gently curved from basal bulb to apex; apical blade poorly evident, very short. Endophallus without an evident lamella copulatrix, but with two very small, apical, and slightly sclerified, ovoidal areas. Left paramere elongate, reaching the distal third and bearing two setae; right paramere shorter and bearing two apical setae.

##### Etymology.

The name comes from type locality “Solomon mining area” and the mythological King of Solomon (in Latin *Salomon*).

##### Distribution.

*Magnanillus salomonis* sp. nov. is known from different deposits of the Solomon Mining Area, 50–60 km N/NW of Tom Price, Pilbara, WA.

**Figures 21–23.**
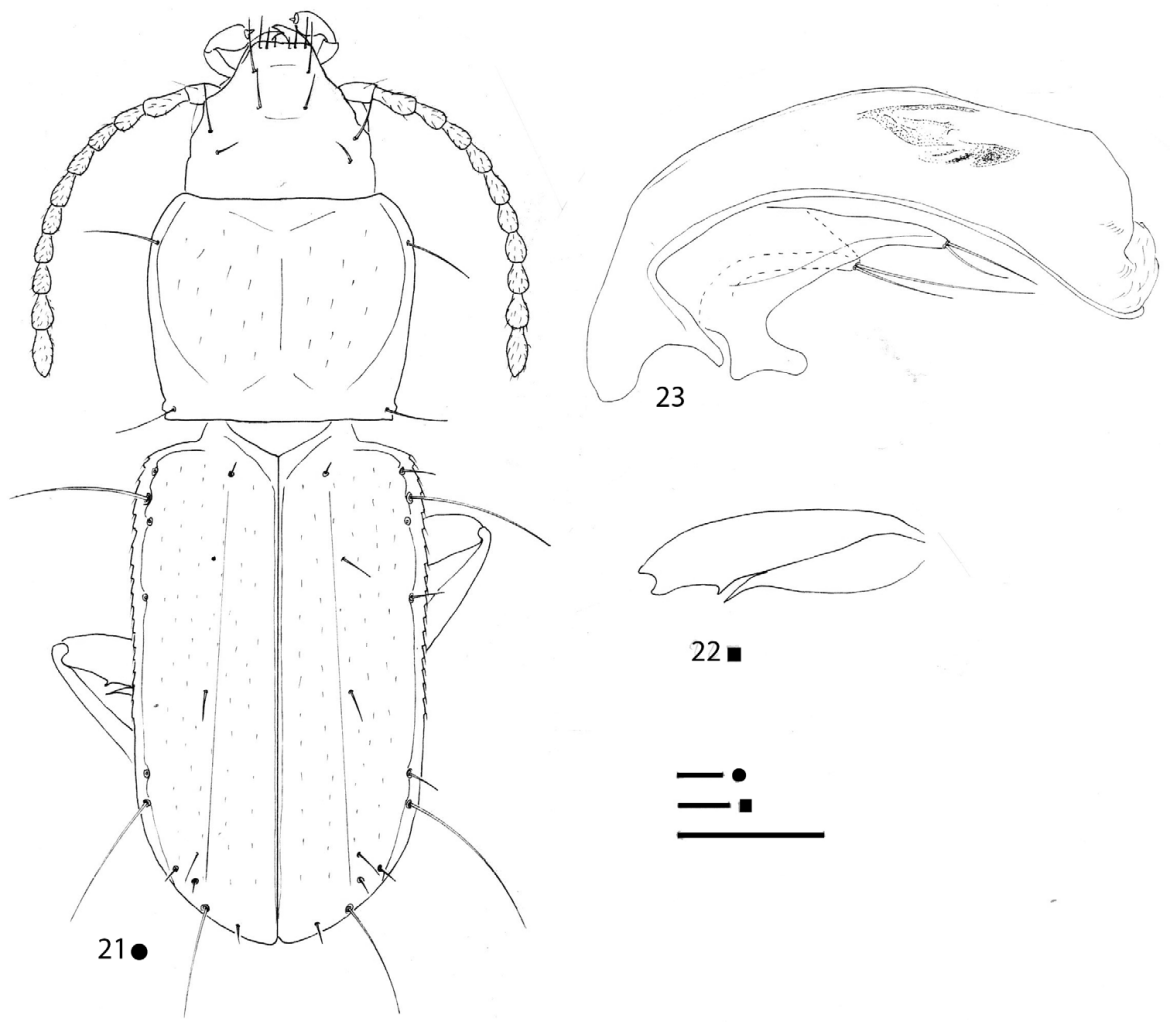
*Magnanillus salomonis* sp. nov., HT ♂ **21** habitus **22** left metafemur and metatrochanter in dorsal view **23** aedeagus in lateral view. Scale bars: 0.1 mm.

#### Magnanillus regalis sp. nov.

Taxon classificationAnimaliaColeopteraCarabidae

2B14A92F-F7F8-5274-B6E7-DB03F931208C

http://zoobank.org/EC1DA36E-EF92-4C88-B595-5E301917D8D8

Figs 24–26

##### Type locality.

WA, Pilbara, 50 km N of Tom Price, Solomon Mining Area, Kings deposit, 22°07'45.5"S, 117°52'24.3"E.

##### Type series.

HT ♂, WA, Pilbara, 50 km N of Tom Price, Solomon Mining Area, Kings Mine, 22°07'45.5"S, 117°52'24.3"E (WGS84), G. Pearson and D. Main, 12 Jan 2010. Trog. net scrape (SM3175), Western Australian Museum Entomology Reg. no. 82653 (WAM). PTT: 1 ♂ 2 ♀♀ (remains), WA, Pilbara, 50 km N of Tom Price, Solomon Mining Area, Kings Mine, 22°07'45.5"S, 117°52'24.3"E (WGS84), G. Pearson and D. Main, 12 Jan 2010. Trog. net scrape (SM3175), Western Australian Museum Entomology Reg. no. 82653 (WAM, CGi).

##### Differential diagnosis.

*Magnanillus regalis* sp. nov. is easily distinguishable from *M. firetailianus* sp. nov. and *M. quartermaini* (Baehr & Main, 2016) by its pronotum with basal border ca. as wide as the anterior border. It can be distinguished from *M. sabae* sp. nov. by its longer metatrochanters, reaching the femoral tooth. It differs from *M. serenitatis* sp. nov. by its shorter metatrochanters, not overreaching the femoral tooth. It can be distinguished from *M. salomonis* sp. nov. by its less transverse pronotum and the straight apex of its metatrochaters.

##### Description of the HT ♂.

TL mm 2.25. ***Body*** elongate, depigmented, yellow-testaceous; integument shiny with evident microsculpture and short pubescence.

**Figures 24–26.**
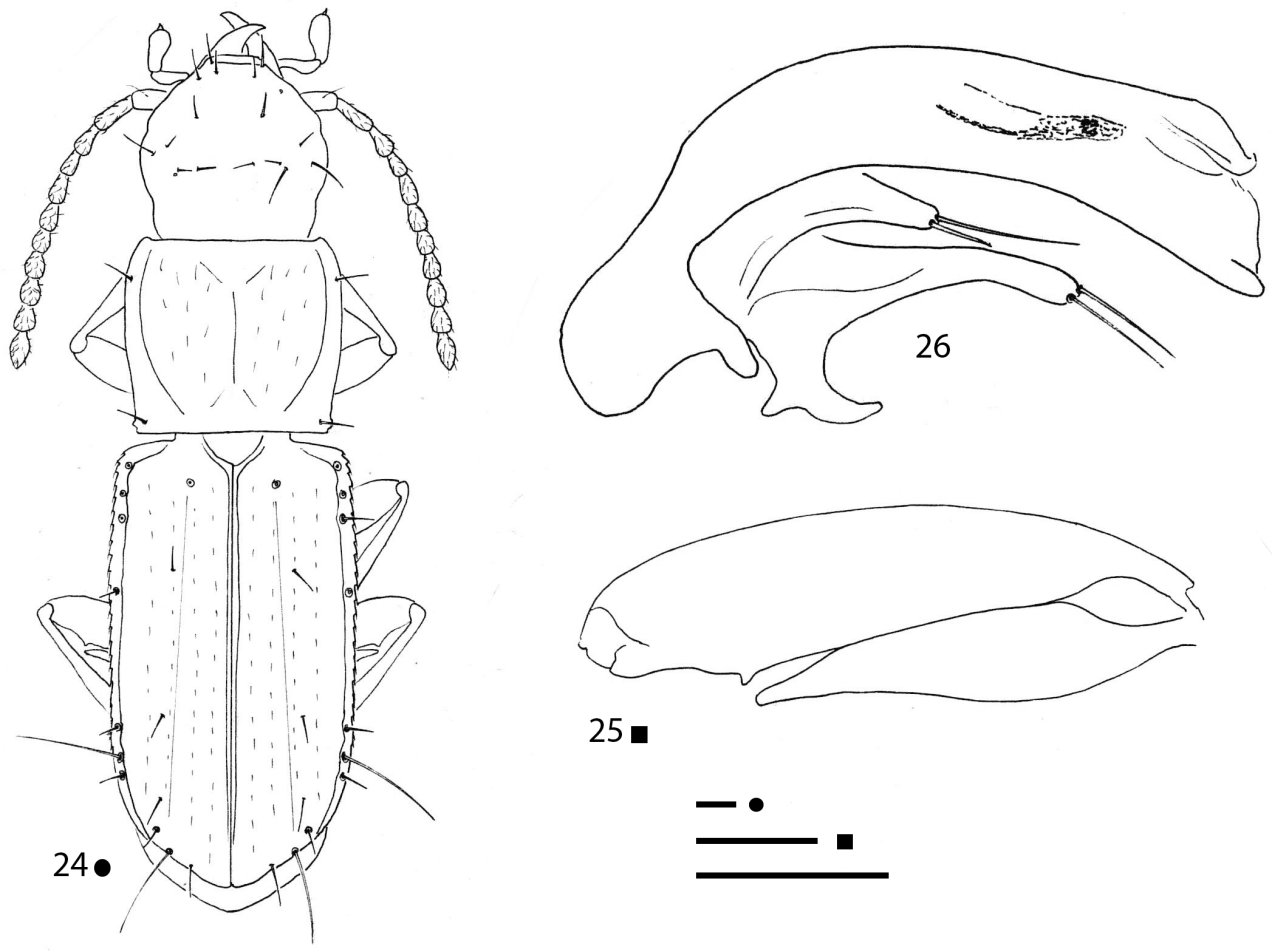
*Magnanillus regalis* sp. nov., HT ♂ **24** habitus **25** right metafemur and metatrochanter in ventral view **26** aedeagus in lateral view. Scale bars: 0.1 mm.

***Head*** relatively large, narrower than pronotum; with two couples of excess setae on the vertex as in Fig. 24. Labium without tooth, mentum articulated. Antennae robust, submoniliform, very short, not reaching the base of the pronotum when stretched backwards. Fronto-clypeal furrow indistinct; anterior margin of the epistome subrectilinear.

***Pronotum*** sub-squared (max. width / max. length ratio = 1.14 maximum width at the base of the anterior fourth, and basal border slightly wider than anterior border; sides slightly and irregularly arcuate in anterior part, subrectilinear at the basal half, not sinuate but with an evident tooth before basal angles. Anterior angles obtuse, slightly prominent; posterior angles right, acute. Disc convex, with very sparse pubescence of medium length; median groove very shallow, hardly evident. Marginal groove wide and flat, enlarged near the base; anterior marginal setae placed inside the marginal groove, almost on the anterior fourth; basal setae slightly placed internally on the disk and before the posterior angles.

***Legs*** long and slender, with metatrochanters long, acuminate, subrectilinear and metafemora dentate; metatrochanters (Fig. 25) reaching the femoral tooth. Two dilated protarsomeres, without adhesive phanerae in males.

***Elytra*** subrectangular, elongate (max. length / max. width ratio = 1.83), not truncated and only slightly emarginated before apex. Disc convex, with longitudinal grooves; integument shiny, with evident microsculpture and very short, longitudinally aligned, upright pubescence. Humeri well marked, obtuse; post-humeral margin denticulate, with distinct crenulations up to the base of the apical third; elytral apices separately rounded. Marginal groove wide and evident almost up to the 8^th^ pore of the umbilicate series.

***Chaetotaxy***: scutellar pore large and foveate. Umbilicate series with the first three pores of the humeral group very closed to each other and equidistant; 4^th^ pore farther and placed at the end of the basal third of the elytron; 5^th^ pore placed at the base the apical third of the elytron; 7^th^ pore very forward, placed near the 6^th^ pore; 6^th^ and 7^th^ pores closer than 5^th^ and 6^th^; 8^th^ displaced onto the disc; 7^th^ and 8^th^ spaced out ca. the 5^th^ and 6^th^. Three discal setae, first placed before the 4^th^ pore of the umbilicate series, second one placed just before the 5^th^ pore, third one placed after the 7^th^ pore.

***Aedeagus*** (Fig. 26) large, median lobe long, slender, gently curved, with basal bulb small but tight and evident; ventral margin gently curved from basal bulb to apex; apical blade poorly evident, short. Endophallus without an evident lamella copulatrix, but with small, Y-shaped, apical, slightly sclerified stripe. Left paramere elongate, reaching the distal third and bearing two setae; right paramere shorter and bearing two apical setae.

##### Etymology.

The name comes from the Latin word *regalis* = royal, and it reminds the type locality “King” deposit in the Solomon Mining Area.

##### Distribution.

*Magnanillus regalis* sp. nov. is known only from the type locality (Kings deposit) in the Solomon Mining Area, 50 km N of Tom Price, Pilbara, WA.

#### Magnanillus serenitatis sp. nov.

Taxon classificationAnimaliaColeopteraCarabidae

78528013-7B63-5A93-9EC0-C1A799D9D09E

http://zoobank.org/D632C476-542A-439B-9EDE-976720B52B66

Figs 27–29

##### Type locality.

WA, Pilbara, 65 km NW of Tom Price, Serenity mining area, Champion deposit, 22°07'5.1"S, 117°26'12.5"E.

##### Type series.

HT ♂, WA, Pilbara, 65 km NW of Tom Price, Serenity mining area, Champion deposit, 22°07'5.1"S, 117°26'12.5"E (WGA84), J. Quartermaine and D. Main, 13 Jun 2011, Trog. trap (HPRC0712), Western Australian Museum Entomology Reg. no. 82667 (WAM). PTT: 2 ♂♂, WA, Pilbara, 50 km N of Tom Price, Solomon Mining Area, Firetail Mine, 22°09'0.0"S, 117°28'55.3"E (WGS84), J. Cocking, D. Main, 23 June 2010, Trog. net scrape (HPRC0243), Western Australian Museum Entomology Reg. no. 82665–82666 (WAM, CGi); 1 ♀, WA, Pilbara, 65 km NW of Tom Price, Serenity mining area, Delta deposit, 22°08'31.0"S, 117°28'1.5"E (WGS84), J. Cocking, D. Main, 19 August 2010, Trog. trap (HPRC2004), Western Australian Museum Entomology Reg. no. 82668 (WAM).

##### Differential diagnosis.

*Magnanillus serenitatis* sp. nov. is easily distinguishable from *M. firetailianus* sp. nov. by its pronotum with basal border as wide as the anterior border. It can be distinguished from *M. sabae* sp. nov., *M. salomonis* sp. nov. and *M. regalis* sp. nov. by its longer metatrochanters, overreaching the femoral tooth.

##### Description.

TL mm 2.27–2.30 ♂♂, 2.32 ♀. ***Body*** elongate, depigmented, testaceous; integument shiny, with evident microsculpture and very short pubescence.

***Head*** relatively small, narrower than pronotum; without excess setae. Labium without tooth, mentum articulated. Antennae robust, submoniliform, short, reaching the base of the pronotum when stretched backwards. Fronto-clypeal furrow indistinct; anterior margin of the epistome subrectilinear.

***Pronotum*** transverse (max. width / max. length ratio = 1.23), maximum width at the base of the anterior third, and basal border slightly wider than anterior border; sides slightly and irregularly arcuate in anterior part, very poorly arcuate in the basal half, not sinuate and denticulate before the basal angles. Anterior angles obtuse, slightly prominent; posterior angles right, acute. Disc convex, with very short and sparse pubescence; median groove very shallow, hardly evident. Marginal groove wide and flat, enlarged near the base; anterior marginal setae placed inside the marginal groove, almost on the anterior fourth; basal setae slightly placed inside the disk and before the posterior angles.

***Legs*** long and slender, with metatrochanters long, acuminate, and curved and metafemora dentate; metatrochanters (Fig. 28) longer than femoral tooth. Two dilated protarsomeres, without adhesive phanerae in males.

***Elytra*** subrectangular, relatively short (max. length / max. width ratio = 1.67), not truncated and only very slightly emarginated before apex. Disc convex, with longitudinal grooves; shiny integument with evident microsculpture and very short, sparse, upright pubescence, not longitudinally aligned. Humeri well marked but rounded; post-humeral margin denticulate, with distinct crenulations up to the base of the apical fourth; elytral apices separately rounded. Marginal groove wide and evident almost down to the 7^th^ pore of the umbilicate series.

***Chaetotaxy***: scutellar pore large and foveate. Umbilicate series with the first three pores of the humeral group very closed to each other and nearly equidistant; 4^th^ pore farther and placed at the end of the basal third of the elytron; 5^th^ pore placed at the base of the apical third of the elytron; 5^th^ and 6^th^ ones spaced out ca. half of the distance between the 6^th^ and the 7^th^; 7^th^ and 8^th^ displaced onto the disc; 7^th^ and 8^th^ spaced from each other as the 8^th^ and 9^th^. Three discal setae, first placed before the 4^th^ pore of the umbilicate series, second one placed after the midpoint of the elytron, third one placed before the 7^th^ pore.

***Aedeagus*** (Fig. 29) large, median lobe long, slender, gently curved, with basal bulb small but tight and evident; ventral margin gently curved from basal bulb to apex; apical blade poorly evident, very short. Endophallus without an evident lamella copulatrix, but with very small, V-shaped, apical, slightly sclerified stripe. Left paramere elongate, reaching the distal third and bearing two setae; right paramere shorter and bearing two apical setae.

##### Etymology.

The name comes from the Serenity Valley, type locality of the species, in the Pilbara region.

##### Distribution.

*Magnanillus serenitatis* sp. nov. is known only from two drill holes in the Serenity Valley, 65 km N/NW of Tom Price, Pilbara, WA.

**Figures 27–29.**
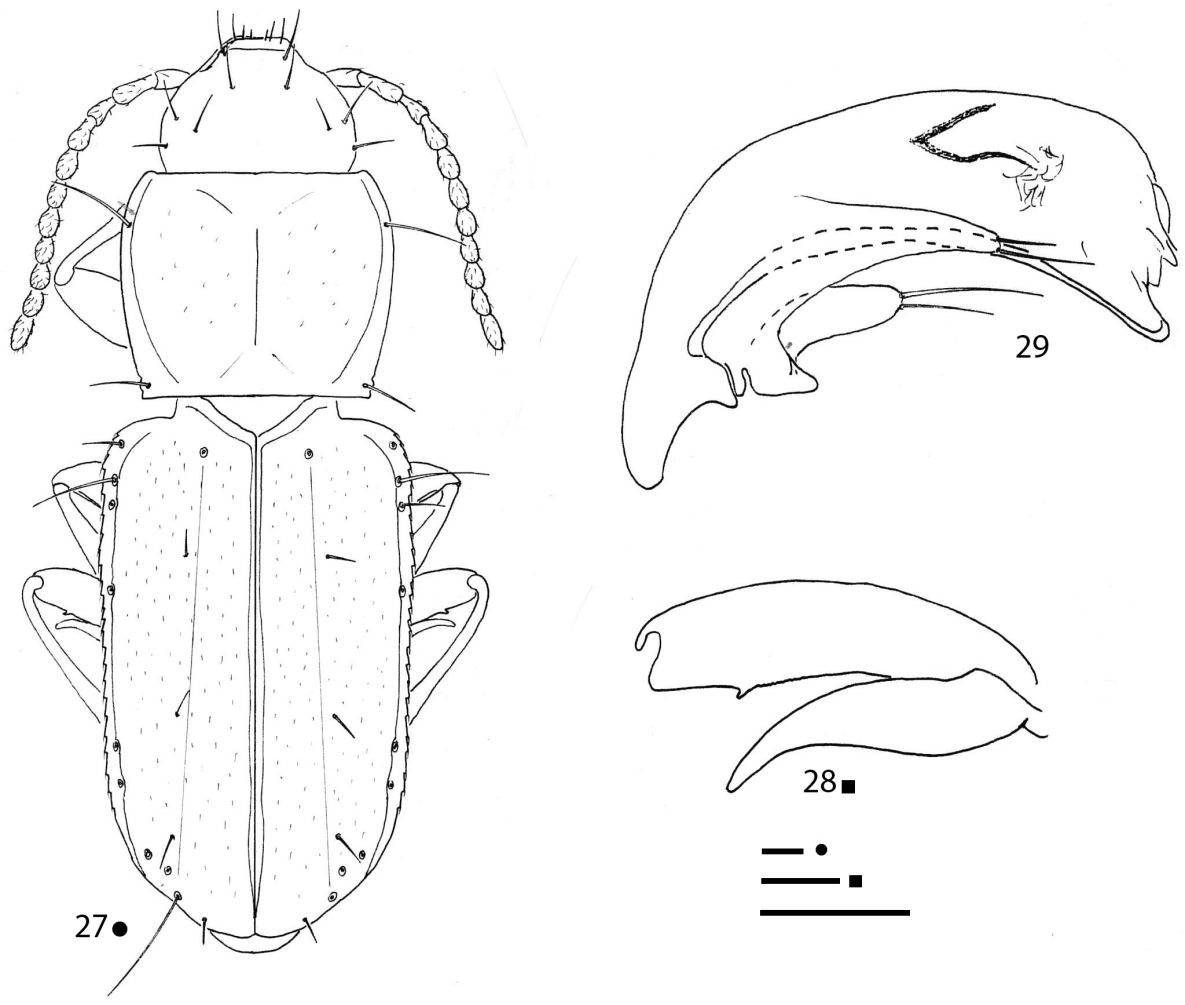
*Magnanillus serenitatis* sp. nov., HT ♂ **27** habitus **28** right metafemur and metatrochanter in ventral view **29** aedeagus in lateral view. Scale bars: 0.1 mm.

#### Neoillaphanus gen. nov.

Taxon classificationAnimaliaColeopteraCarabidae

3B2B4B9D-3C11-5DB0-B14B-11D6189B613F

http://zoobank.org/AEBE850A-1176-47A1-BB3E-A71C27FA4A9C

Figs 30–33

##### Type species.

*Neoillaphanus callawanus* sp. nov.

##### Diagnosis.

Genus characterised by: metatrochanters long and sharp; metafemora non dentate; elytra not reduced at tip; elytral disc with longitudinal grooves and bearing one seta; 9^th^ pore of the umbilicate series in normal position (placed after the 8^th^ one); aedeagus with median lobe curved, size of the basal bulb normal and parameres bearing two apical setae. Labial tooth absent.

##### Description.

Species of medium size (TL mm 1.93–2.04) and anophthalmous. Integument depigmented but well sclerified and covered with sparse pubescence.

***Head*** large but narrower than pronotum; mandibles short and simple, without hyperplasias. Maxillary palpi ovoidal, swollen. Labium transverse, articulated; mentum not fused with submentum. Labial tooth absent. Antennae moniliform, without particular features.

***Pronotum*** cordiform, sides sinuate at the basal third, denticulated before basal angles. Basal angles right, sharp at tips, not rounded; basal border narrower than anterior border; presence of two marginal setae, the posterior one placed before basal angles.

***Elytra*** ovoidal short, separately rounded, not truncated and apically not emarginated; convex, with longitudinal grooves. Elytral striae absent (except sutural stria). Lateral margin starting from the humeral area, distinctly crenulate up to its half-length.

Scutellar pore present, large and umbilicate; umbilicate series of type B (sensu Jeannel 1963; Giachino and Vailati 2011); disc bearing one seta.

***Legs*** relatively long and slender. Pro- and metafemora unarmed; metatrochanters long and sharp, two dilated protarsomeres in the male.

***Aedeagus*** relatively large, median lobe long and curved with basal bulb of normal size. Parameres long, each of them bearing two apical setae. Endophallus with very small and poorly sclerified phanerae.

##### Etymology.

The name is derived by the combination of the Greek prefix *Neo*- (means new) and the name *Illaphanus*. Gender masculine.

##### Species included.

Currently only one species belongs to this genus: *N. callawanus* sp. nov.

**Figures 30–33.**
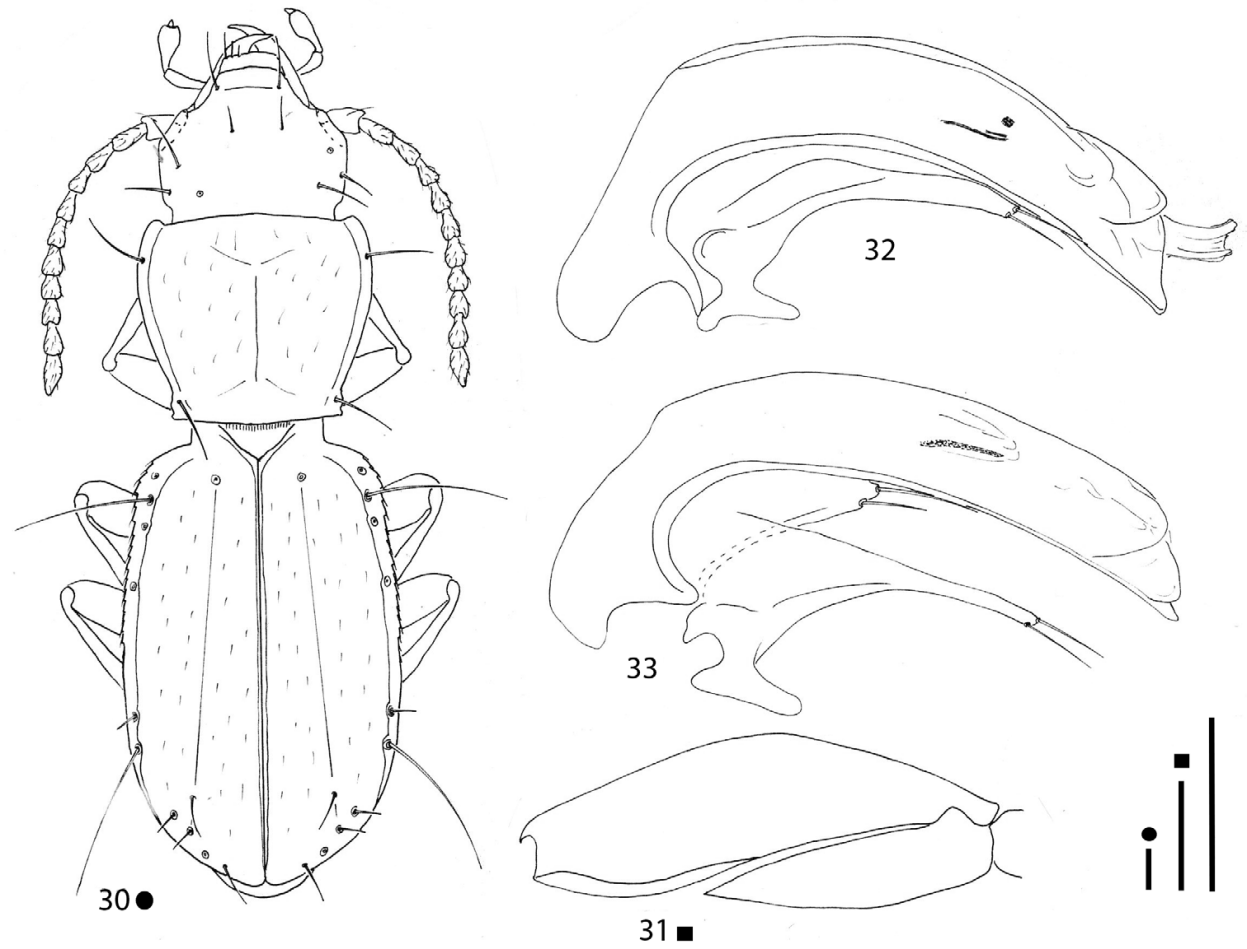
*Neoillaphanus callawanus* gen. et sp. nov. **30** habitus HT ♂ **31** right metafemor and metatrochanter in ventral view, HT ♂ **32** aedeagus in lateral view, HT ♂ **33** aedeagus in lateral view, PT ♂. Scale bars: 0.1 mm.

#### Neoillaphanus callawanus sp. nov.

Taxon classificationAnimaliaColeopteraCarabidae

BEE8065A-BB5B-51C0-A589-1B538032E2F7

http://zoobank.org/77C334DE-F24F-4C50-9AB1-ECBDB445D6D0

Figs 30–33

##### Type locality.

WA, Pilbara, 200 km E of Port Hedland, Goldsworthy Mining Area, Callawa deposit, 20°38'16.74"S, 120°18'14.33"E.

##### Type series.

HT ♂, WA, Pilbara, 200 km E of Port Hedland, Goldsworthy Mining Area, Callawa Mine (drill hole CA0013R), 20°38'16.74"S, 120°18'14.33"E (WGS84), 29 Jul. 2008, P. Bell, Trog trap, Western Australian Museum Entomology Reg. no. 82631 (WAM). PTT: 1 ♂, WA, Pilbara, 200 km E of Port Hedland, Goldsworthy Mining Area, Callawa Mine (drill hole CA0011R) 20°38'16.74"S, 120°18'14.33"E (WGS84), 9 July 2009, P. Bell, Trog net scrape, Western Australian Museum Entomology Reg. no. 82629 (WAM); 1 ♀, WA, Pilbara, 200 km E of Port Hedland, Goldsworthy Mining Area, Callawa Mine (drill hole CA0021R), 20°38'16.74"S, 120°18'14.33"E (WGS84), 29 July 2008, P. Bell, Stygo net haul, Western Australian Museum Entomology Reg. no. 82628 (WAM); 1 ♂, WA, Pilbara, 200 km E of Port Hedland, Goldsworthy Mining Area, Callawa Mine (drill hole CA0011R), 20°38'16.74"S, 120°18'14.33"E (WGS84), 12 June 2009, P. Bell, Stygo net haul, Western Australian Museum Entomology Reg. no. 82630 (CGi); 1 ♀, WA, Pilbara, 200 km E of Port Hedland, Goldsworthy Mining Area, Callawa Mine (drill hole CA0102R), 20°38'16.74"S, 120°18'14.33"E (WGS84), 29 April 2008, P. Bell, Stygo net haul, Western Australian Museum Entomology Reg. no. 82627 (WAM); 1 ♀, WA, Pilbara, 200 km E of Port Hedland, Goldsworthy Mining Area, Callawa Mine (drill hole CA0124R), 20°38'53"S, 114°17'57"E, 28 July 2008, Subterranean Ecology, hauling, Western Australian Museum Entomology Reg. no. 72011 (CGi); 1 ♂, WA, Pilbara, 200 km E of Port Hedland, Goldsworthy Mining Area, Callawa Mine (drill hole CA0008R), 20°38'34"S, 120°18'02"E, 31 May 2009, Subterranean Ecology, hauling, Western Australian Museum Entomology Reg. no. 72012 (WAM); 1 ♀, WA, Pilbara, 200 km E of Port Hedland, Goldsworthy Mining Area, Callawa Mine, 20°38'46"S, 120°17'50"E, 12 June 2009, Subterranean Ecology, bore CA0019R, scraping, Western Australian Museum Entomology Reg. no. 72013 (WAM).

##### Diagnosis.

Species identified by the characters listed in the genus diagnosis.

##### Description.

TL mm 1.93–1.97 ♂♂, 2.00–2.04 ♀♀. ***Body*** elongated, depigmented, testaceous; integument shiny, with evident microsculpture and medium length pubescence.

***Head*** large, narrower than pronotum; without excess setae. Labium without tooth, mentum articulated. Antennae robust, moniliform, short, reaching the base of the pronotum when stretched backwards. Fronto-clypeal furrow distinct; anterior margin of the epistome subrectilinear.

***Pronotum*** cordiform (max. width / max. length ratio = 1.13), maximum width at the base of the anterior third, and basal border narrower than anterior border; sides slightly and regularly arcuate in anterior part, subrectilinear at the basal half, sinuate and denticulate before basal angles. Anterior angles obtuse, prominent; posterior angles right, sharp at tips. Disc convex, with sparse pubescence of medium length; median groove very shallow, hardly evident. Marginal groove wide and flat, poorly enlarged near the base; anterior marginal setae placed inside the marginal groove, almost on the anterior fifth; basal setae not on the disk and placed before posterior angles.

***Legs*** long and slender, with metatrochanters long and acuminate but not curved and metafemora unarmed; metatrochanters (Fig. 31) as long as 2/3 of the femoral length. Two poorly dilated protarsomeres, without adhesive phanerae in males.

***Elytra*** ovoidal, relatively short (max. length / max. width ratio = 1.8), maximum width at the base of the posterior third, not truncated and not emarginated in preapical zone. Disc convex, with longitudinal grooves; integument shiny with evident microsculpture and pubescence of medium length, sparse and upright, not longitudinally aligned. Humeri rounded; post-humeral margin denticulate, with distinct crenulations up to half-length; elytral apices separately rounded. Marginal groove wide and evident almost up to the 7^th^ pore of the umbilicate series.

***Chaetotaxy***: scutellar pore large, foveate. Umbilicate series with the first three pores of the humeral group very closed to each other and nearly equidistant; 4^th^ pore farther and placed at the end of the basal third of the elytron; 5^th^ pore placed just before the base the apical third of the elytron; 5^th^ and 6^th^ ones spaced out ca. half of the distance between 6^th^ and 7^th^; 7^th^ and 8^th^ displaced onto the disc; 7^th^ and 8^th^ spaced out ca. as the 8^th^ and 9^th^. One discal seta placed just before the 7^th^ pore of the umbilicate series.

***Aedeagus*** (Figs 32, 33) large, median lobe long, slender, curved, with basal bulb tight and evident; ventral margin regularly curved from basal bulb to apex; apical blade evident, but short. Endophallus without an evident lamella copulatrix, but with very small, preapical, slightly sclerified stripe. Left paramere elongate, reaching the distal third of median lobe and bearing two setae; right paramere shorter and bearing two apical setae.

##### Etymology.

The name comes from the Callawa Ridge (type locality) in the NE of the Pilbara region.

##### Distribution.

*Neoillaphanus callawanus* sp. nov. is known only from a few drill holes on the Callawa Ridge, 200 km E of Port Hedland, Pilbara, WA.

#### Kimberleytyphlus gen. nov.

Taxon classificationAnimaliaColeopteraCarabidae

B7E825B3-9D9B-599C-B651-811C3E1B7D3C

http://zoobank.org/B98AC026-EEAD-43A7-B4B0-CF5121A12836

Figs 34–35

##### Type species.

*Kimberleytyphlus carrboydianus* sp. nov.

##### Diagnosis.

Genus of the “*Illaphanus* phyletic series” (sensu Giachino 2005), with species strongly characterised by normal metatrochanters; metafemora non dentate; elytra not reduced at tip; elytral disc without longitudinal grooves and bearing three setae, scaly microsculpture and 9^th^ pore of the umbilicate series in normal position (placed after the 8^th^ one); aedeagus with median lobe subrectilinear, size of basal bulb normal and parameres each bearing one apical seta. Labial tooth absent.

##### Description.

Species of a medium size (TL mm 1.57–1.59) and anophthalmous. Integument depigmented but well sclerified, with strong microsculpture and covered with short and sparse pubescence.

***Head*** large but slightly narrower than pronotum; mandibles short and simple, without hyperplasias. Maxillary palpi ovoidal, swollen. Labium transverse, articulated; mentum not fused with the submentum. Labial tooth absent. Antennae moniliform, without particular features.

***Pronotum*** trapezoidal, with sides not sinuate in the basal third, denticulated at the basal third. Basal angles obtuse, sharp, not rounded; basal border slightly narrower than anterior border; presence of two marginal setae, the posterior one placed near basal angles.

***Elytra*** subrectangular elongate, separately rounded, not truncated and apically only slightly emarginated, convex, without longitudinal grooves. Elytral striae absent (except sutural stria). Lateral margin starting from the humeral area and distinctly crenulate up to the level of 8^th^–9^th^ pores of the umbilicate series. Scutellar pore present, large and umbilicate; umbilicate series of type B (sensu Jeannel 1963; Giachino and Vailati 2011); disc bearing three setae.

***Legs*** relatively long and slender. Pro- and metafemora unarmed; metatrochanters normal, two slightly dilated protarsomeres, without adhesive phanerae in males.

***Aedeagus*** relatively small, median lobe long, subrectilinear with basal bulb of normal size. Parameres long, bearing one apical seta. Endophallus without any sclerified phanerae.

##### Etymology.

The name combines the name Kimberley (region where the type locality is located) and the Greek suffix -*typhlos* (meaning blind).

##### Species included.

Currently only *K. carrboydianus* sp. nov. belongs to this genus.

**Figures 34, 35.**
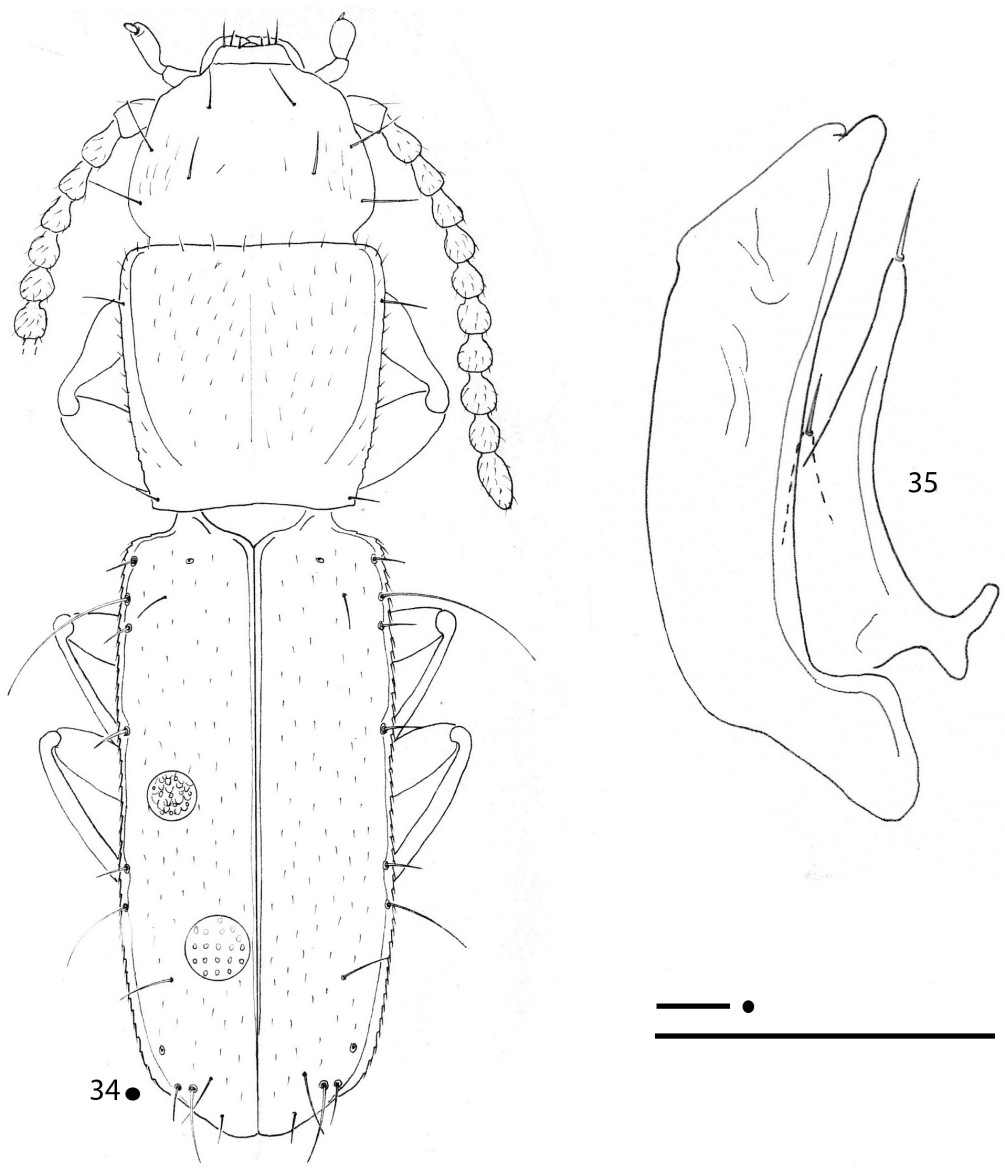
*Kimberleytyphlus carrboydianus* gen. et sp. nov., HT ♂ **34** habitus **35** aedeagus in lateral view. Scale bars: 0.1 mm.

#### Kimberleytyphlus carrboydianus sp. nov.

Taxon classificationAnimaliaColeopteraCarabidae

E6DEB6FA-C1E5-5E82-B1BB-65DFBE41FD77

http://zoobank.org/20CF7CE3-BE40-4957-9B2D-7C44DB803300

Figs 34–35

##### Type locality.

WA, Kimberley, Carr-Boyd Ranges, 530 km SE Darwin, 16°37'26.02"S, 128°15'32.36"E.

##### Type series.

HT ♂, WA, Carr-Boyd Ranges, 530 km SE Darwin, Kimberley, WA, 16°37'26.02"S, 128°15'32.36"E (WGS84), Animal Plant Mineral (APM), 12 August 2009, Trog. net scrape (APM-KMGTROG 7-LN7274), Western Australian Museum Entomology Reg. no. 82632 (WAM). PTT: 1 ♂, WA, Kimberley, Carr-Boyd Ranges, 530 km SE Darwin, 16°37'52.59"S, 128°15'32.83"E (WGS84), Animal Plant Mineral (APM), 12 Aug. 2009, Trog. net scrape (APM-KMGTROG 8-LN7276), Western Australian Museum Entomology Reg. no. 82633 (CGi).

##### Diagnosis.

Species identified by the characters listed in the genus diagnosis.

##### Description.

TL mm 1.57–1.59 ♂♂. ***Body*** elongate, depigmented, testaceous; integument with medium length pubescence. Microsculpture evident and very strong: composed by isodiametric meshes on pronotal disc; scaly on basal part of elytral disc, and longitudinally oriented hollow points on apical elytral disc (Fig. 34).

***Head*** large, slightly narrower than pronotum; without excess setae. Labium without tooth, mentum articulated. Antennae robust, moniliform, short, reaching the base of the pronotum when stretched backwards. Fronto-clypeal furrow indistinct; anterior margin of the epistome subrectilinear.

***Pronotum*** trapezoidal (max. width / max. length ratio = 1.00), maximum width at the base of the anterior fourth, with basal border narrower than anterior border; sides subrectilinear, anteriorly slightly but regularly arcuate, not arcuate and not sinuate at the basal half, denticulated at the basal third. Anterior angles obtuse, rounded and not prominent; posterior angles obtuse, but evident. Disc convex, with sparse pubescence of medium length; median groove very shallow, hardly evident. Marginal groove wide and flat, enlarged near the base; anterior marginal setae placed inside the marginal groove, almost on the anterior fourth; basal setae not on the disk and placed at posterior angles.

***Legs*** long and slender, with metatrochanters normal and metafemora unarmed. Two poorly dilated protarsomeres, without adhesive phanerae in males.

***Elytra*** subrectangular, very elongated (max. length / max. width ratio = 2.20), maximum width at the base of the posterior third, not truncated and slightly emarginated before apex. Disc convex, without longitudinal grooves; integument with sparse and upright pubescence, longitudinally aligned. Humeri well marked; post-humeral margin denticulate, with distinct crenulations up to the 8^th^ and 9^th^ pores of the umbilicate series; elytral apices separately rounded. Marginal groove wide and evident almost up to the 9^th^ pore of the umbilicate series.

***Chaetotaxy***: scutellar pore small and foveate. Umbilicate series with the first three pores of the humeral group very closed to each other and nearly equidistant; 4^th^ pore farther and placed at the end of the basal third of the elytron; 5^th^ pore placed just after the middle of the elytron; 5^th^ and 6^th^ ones spaced out ca. 1/4 of the distance between 6^th^ and 7^th^; 7^th^ and 9^th^ placed onto the disc; 7^th^ and 8^th^ spaced out ca. double of the distance from 8^th^ and 9^th^; 9^th^ placed at the level of the 8^th^ pore. Three discal setae, first placed at the level of the 2^nd^ pore of the umbilicate series, second one placed at the level of the base of the posterior fourth of elytron, third one placed just before the level of the 9^th^ pore.

***Aedeagus*** (Fig. 35) relatively small, median lobe long, slender, subrectilinear, with basal bulb tight and evident; ventral margin weakly curved from basal bulb to apex; apical blade evident, but short. Endophallus without any sclerified phanerae. Left paramere very elongated, reaching the distal fifth of the median lobe, and bearing only one seta; right paramere shorter than left one, bearing one apical seta.

##### Etymology.

The name derives from the name of one of the early European explorers to visit the region named as the Carr-Boyd Ranges (type locality). In 1883 William Henry James Carr-Boyd became second-in-command of an expedition led by W. J. O’Donnell on behalf of the Cambridge Downs Pastoral Association; their purpose was to explore the country around the Cambridge Gulf, and to establish a sheep station. The party of six men, including a cook and an Aboriginal boy, twenty-six horses and provisions for six months, left Katherine on 26 March 1883. O’Donnell named the impressive Carr Boyd Range after his second-in-command on 26 May.

##### Distribution.

*Kimberleytyphlus carrboydianus* sp. nov. occurs only at the type locality Carr-Boyd Ranges, 530 km SE Darwin, Kimberley, WA.

#### Austranillus

Taxon classificationAnimaliaColeopteraCarabidae

Giachino, 2005

E6438A92-A0B3-5CFE-B0A4-6237EE488E15

Figs 36–37

##### Type species.

*Austranillus macleayi* (Lea, 1906)

##### Diagnosis.

Genus of the “*Illaphanus* phyletic series” (sensu Giachino 2005), with species characterised by: absence of longitudinal elytral grooves, pronotum with basal border wider than anterior border and denticulate sides before basal angles, elytral disc with two setae, 8^th^ pore of the umbilicate series after the 9^th^ (sensu Giachino and Vailati 2011), labium toothless, median lobe of the aedeagus long and weakly curved, and parameres very long and slender.

##### Note.

In the original description of the genus Giachino (2005) does not mention the forward position of the 9^th^ pore of the umbilicate series. The omission was due to the lost seta corresponding to the 9^th^ pore, and to the extended length of secondary setae (1^st^, 3^rd^, 4^th^, 5^th^, 7^th^, and 8^th^) in *A. macleayi*. The study of the umbilicate series of *A. jinayrianus* sp. nov. indicates, according to Giachino and Vailati (2011) that the 8^th^ pore is actually placed backwards in respect to the 9^th^.

##### Species included.

Currently, two species belong to this genus:

*Austranillus macleayi* (Lea, 1906)

*Austranillus jinayrianus* sp. nov.

**Figures 36, 37.**
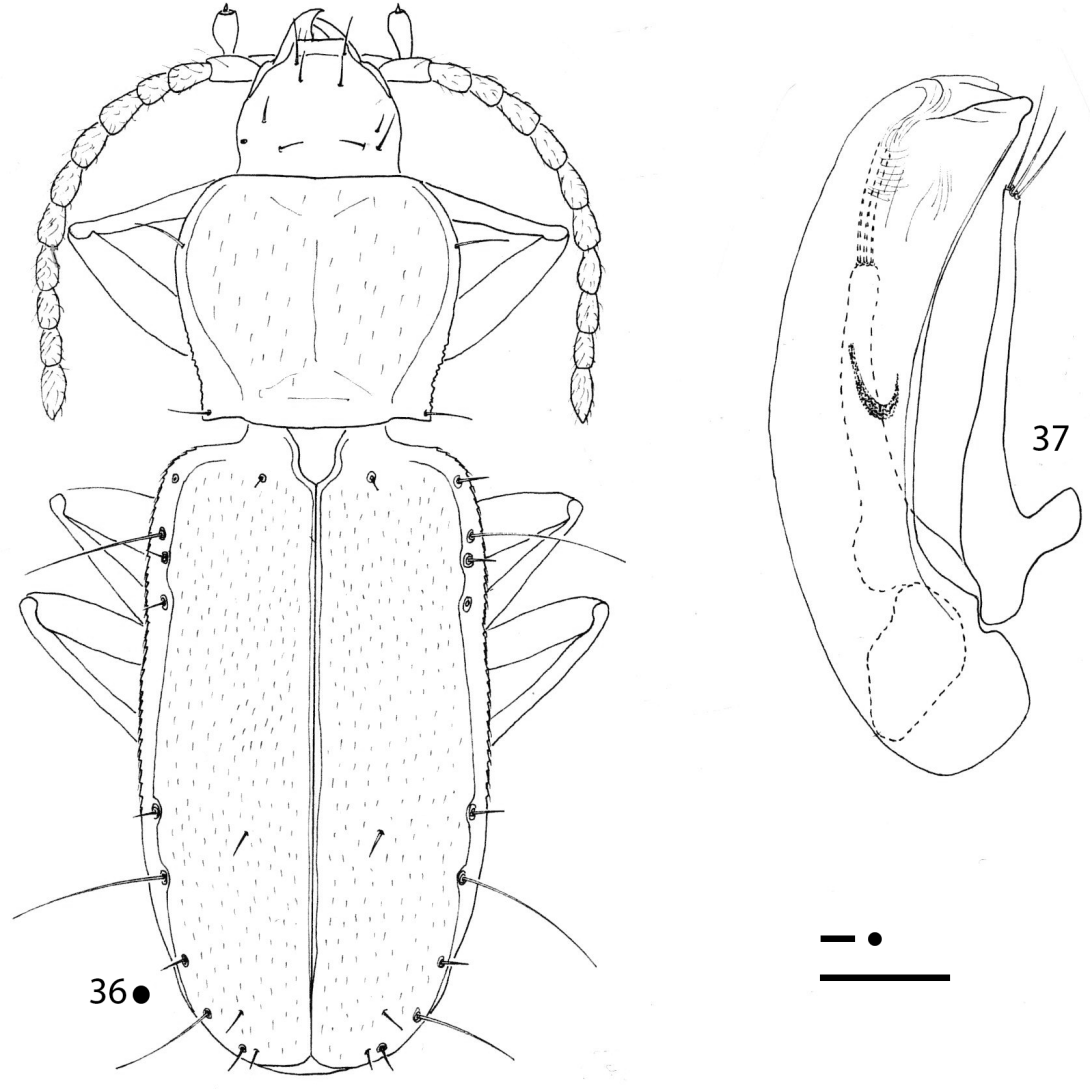
*Austranillus jinayrianus* sp. nov., HT ♂ **36** habitus **37** aedeagus in lateral view. Scale bars: 0.1 mm.

### Key to the species of *Austranillus*

**Table:** 

1	Anterior discal pore placed at the base of the anterior third of elytra	***A. macleayi* (Lea, 1906)**
–	Anterior discal pore placed at the base of the posterior third of elytra	***A. jinayrianus* sp. nov.**

#### Austranillus jinayrianus sp. nov.

Taxon classificationAnimaliaColeopteraCarabidae

6DE48945-5B5D-5E45-B776-701B8D02FDC8

http://zoobank.org/52F0EC77-B63A-49DA-868C-9637C3B5E0AF

Figs 36–37

##### Type locality.

WA, Pilbara, 110 km NW of Newman, Area C Mining Area, Jinayri deposit, 22°58'22.4"S, 119°15'37.9"E.

##### Type series.

HT ♂, WA, Pilbara, 110 km NW of Newman, Area C Mining Area, Jinayri Mine, 22°58'22.4"S, 119°15'37.9"E (WGS84), J. Cocking and M. Scanlon, 25 Nov. 2008. Trog. trap (JIN0744), Western Australian Museum Entomology Reg. no. 82674 (WAM).

##### Differential diagnosis.

Very large species (TL mm 2.98), easily distinguishable from *A. macleayi* by its larger size (mm 2.98 *vs.* mm 1.76 in *A. macleayi*), by the position of the 4^th^ pore of the umbilicate series placed further, and by the position of the anterior discal pore which is placed at the base of the posterior third of elytra.

##### Description of the HT ♂.

TL mm 2.98. ***Body*** elongated, depigmented, fulvo-testaceous; integument shiny, with evident microsculpture and sparse and short pubescence.

***Head*** very small, much narrower than pronotum. Labium toothless. Antennae long and robust, not moniliform (with relatively elongated antennomeres), considerably exceeding the base of the pronotum when stretched backwards. Fronto-clypeal furrow indistinct; anterior margin of the epistome subrectilinear.

***Pronotum*** slightly transverse (max. width / max. length ratio = 1.13) with maximum width at the anterior third, and a very wide basal border, wider than the anterior border. Pronotum sides anteriorly clearly arcuate, weakly sinuate and denticulate before the base, base laterally distinctly emarginated. Anterior angles rounded, not prominent. Posterior angles protruding, acute at the vertex. Disc slightly convex, with a short and relatively dense pubescence; median groove very shallow, slightly marked. Marginal groove relatively wide and flat, very enlarged near the base; anterior marginal setae inserted inside the marginal groove, on approximately the anterior third; basal setae inserted almost on the posterior angles.

***Legs*** long and slender, with metatrochanters normal and metafemora unarmed. Two asymmetrically dilated protarsomeres, with one row of adhesive phanerae in male.

***Elytra*** subrectangular, very elongated (max. length/max. width ratio = 1.77), with maximum width in the middle, very slightly emarginated before apex; sides slightly sinuate at the base of the anterior fourth. Disc poorly convex; integument shiny, with evident microsculpture and short, dense, upright pubescence. Humeri extremely marked, almost right angle; post-humeral margin denticulate, with a distinct crenulation up to the base of the apical third of the elytron; elytral apices separately rounded. Marginal groove wide and evident up to over the 9^th^ pore of the umbilicate series.

***Chaetotaxy***: scutellar pore large, foveate. Umbilicate series with pores of the humeral group not equidistant; 4^th^ pore slightly displaced towards the disc, not so far from the third pore and inserted just on the basal fourth of the elytron; 5^th^ pore placed just before the apical third of the elytron; 5^th^ and 6^th^ pores spaced out, ca. 2/3 of the 6^th^ and 7^th^; 7^th^, 8^th^ and 9^th^ pores almost equidistant and slightly displaced onto the disc, 8^th^ pore placed after the 9^th^ one. Two discal setae, first placed at the base of the apical third, second one placed at the level of the 9^th^ pore.

***Aedeagus*** (Fig. 37) large, median lobe long, slender, subrectilinear, with basal bulb poorly evident; ventral margin poorly and gently curved from the basal bulb to the apex; apical blade evident, but short and emarginated in the lower edge. Endophallus with a median, C-shaped, sclerified phanera. Parameres slender and very elongated, reaching the distal seventh of the median lobe, and bearing three setae only; right paramere slightly shorter than the left.

##### Etymology.

The name of the species comes from the type locality of the Jinayri deposit.

##### Distribution.

*Austranillus jinayrianus* sp. nov. is known only from the type locality, Jinayri deposit, in the Pilbara region, 110 km NW of Newman, WA.

#### Gilesdytes gen. nov.

Taxon classificationAnimaliaColeopteraCarabidae

7A915CA1-F70D-523D-93F3-65AE9169B42C

http://zoobank.org/74939AC7-BAE8-40F9-8D7E-AA32F7678E69

Figs 38–42

##### Type species.

*Gracilanillus vixsulcatus* Baehr & Main, 2016

##### Diagnosis.

Species of this genus are strongly characterised by: lacking longitudinal elytral grooves, pronotum with basal border as wide as the anterior border, sides posteriorly not sinuate, and denticulate before, or near, the basal angles, elytral disc bearing one (posterior) seta placed at the level, or after, the 7^th^ umbilicate pore, 8^th^ pore of the umbilicate series located after the 9^th^ (sensu Giachino and Vailati 2011), toothed labium, median lobe of the aedeagus long and weakly curved with parameres very long and slender.

Differs from *Gracilanillus* Baehr & Main, 2016 by bearing a single discal seta on the elytral disc (2 in *Gracilanillus*), and for the absence of longitudinal elytral grooves.

##### Description.

Included medium size species (TL mm 1.29–2.11), and anophthalmous. Integument depigmented but well sclerified, with strong microsculpture and covered by short and moderately dense pubescence.

***Head*** large, slightly narrower than pronotum; mandibles short and simple, without hyperplasias. Maxillary palps ovoidal, swollen. Labium transverse, articulated; mentum not fused with submentum. Labial tooth present. Antennae moniliform, without particular features.

***Pronotum*** squared, sides not sinuate on the basal third, denticulated on the basal third or just before the posterior angles. Basal angles right, sharp, not rounded; basal border as wide as the anterior border; two marginal setae, posterior seta placed near the basal angles.

***Elytra*** elongated and subrectangular, separately rounded, not truncate and not apically emarginated; convex, without longitudinal grooves. Elytral striae absent (except for sutural stria). Lateral margin starting from the humeral area, distinctly serrulate up to the level of 8^th^-9^th^ pores of the umbilicate series.

Scutellar pore present, large and umbilicate; umbilicate series of type B (sensu Jeannel 1963; Giachino and Vailati 2011) with the 8^th^ pore placed after the 9^th^; disc bearing one seta.

***Legs*** relatively long and slender. Pro- and metafemora unarmed; metatrochanters normal, two slightly dilated protarsomeres, without adhesive phanerae, in the male.

***Aedeagus*** relatively small, median lobe long, subrectilinear with basal bulb of normal size. Parameres long, bearing two apical setae. Endophallus with a sinuate, slightly sclerified, apical phanera.

##### Etymology.

*Gilesdytes*: name composed by two sections, the first one (Giles) dedicated to William Ernest Powell Giles (20 July 1835 – 13 November 1897), Australian explorer who in 1876 named the Ophthalmia Range (type locality of the genus); and the second one (*dytes*) for diver.

##### Species included.

Currently three species belong to this genus:

*Gilesdytes vixsulcatus* (Baehr & Main, 2016)

*Gilesdytes ethelianus* sp. nov.

*Gilesdytes pardooanus* sp. nov.

**Figures 38–42.**
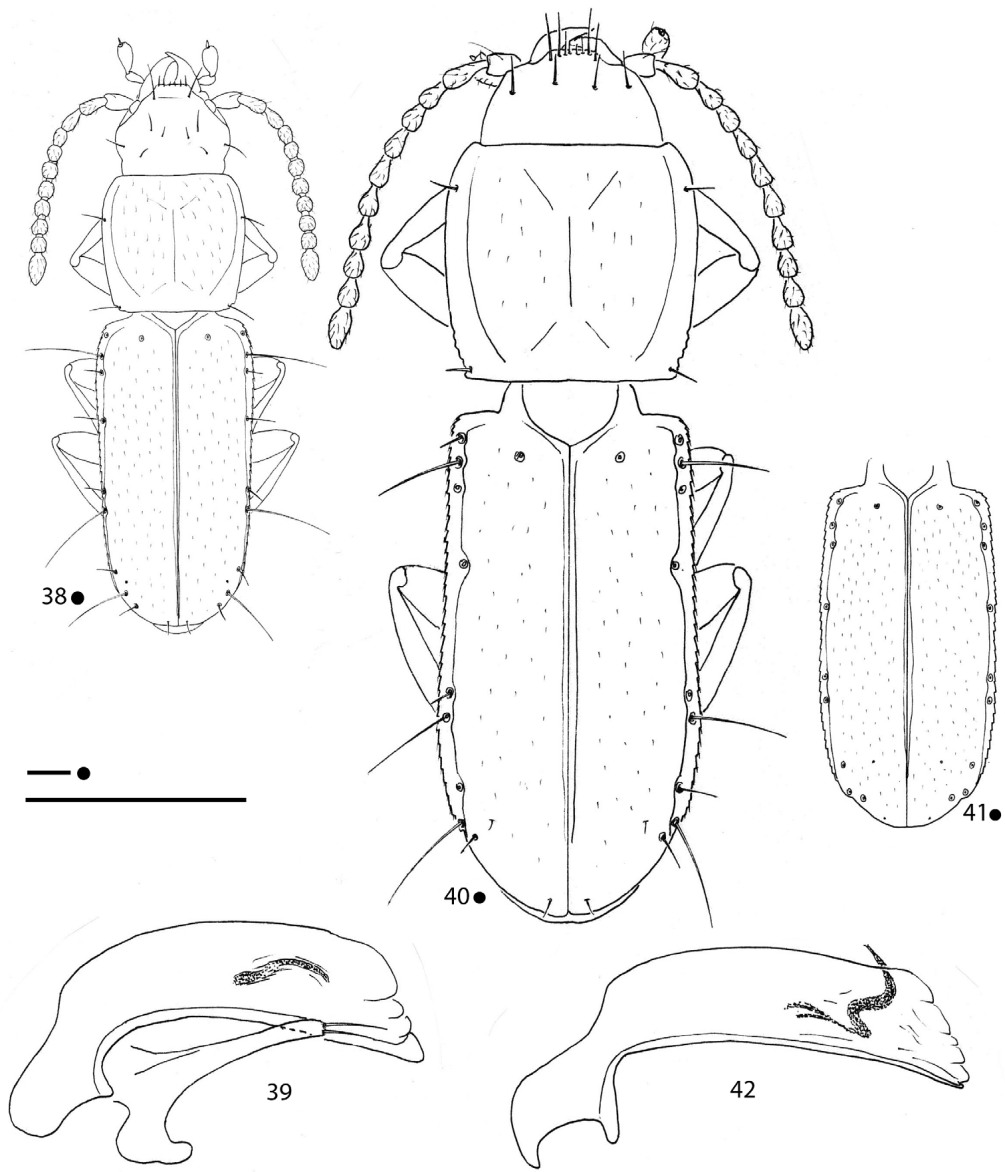
*Gilesdytes* gen. et spp. nov., habitus (**38, 40, 41**) aedeagus in lateral view (**39, 42**) **38, 39***G. vixsulcatus* (Baehr and Main), ♂ **40***G. pardooanus* sp. nov., HT ♀ **41, 42***G. ethelianus* sp. nov., HT ♂. Scale bars: 0.1 mm.

### Key to the species of *Gilesdytes*

**Table:** 

1	Discal pore placed on the middle width of elytron	***G. ethelianus* sp. nov.**
–	Discal pore not placed on the middle width of elytron, but moved towards the edge	**2**
2	Pronotum with lateral edge, near the basal angle, smooth. Apical group of umbilicate pores placed on the apical 5^th^ of elytron	***G. vixsulcatus* (Baehr & Main)**
–	Pronotum with lateral edge, near the basal angle, denticulate. Apical group of umbilicate pores placed on the apical 4^th^ of elytron	***G. pardooanus* sp. nov.**

#### Gilesdytes vixsulcatus

Taxon classificationAnimaliaColeopteraCarabidae

(Baehr & Main, 2016)

5880E919-986B-510B-922C-5271E3CBE388

Figs 38–39

##### Type locality.

WA, Pilbara, Marillana Creek, c. 85 km NNW Newman, 22°41'53.30"S, 119°20'28.80"E.

##### Material examined.

1 ♂, WA, Pilbara, 90 km NW of Newman, near Yandi mining area, Ministers North deposit, 22°49'26.54"S, 119°05'23.89"E (WGS84), 20 Feb. 2009, P. Bell, G. Perina, Trap, (BHP010-EXR1154-MNL:7056) Western Australian Museum Entomology Reg. no. 82619 (WAM).

##### Note.

Baehr and Main (2016) description of this species is based on only one female specimen. They assigned the genus *Gracilanillus* based on the presence of inconspicuous longitudinal grooves at the base of elytra. The study of a male morphologically comparable to the habitus drawing provided by Baehr and Main (2016) and collected from a locality close to the *Gracilanillus vixsulcatus* type locality, allowed the description of the male of this species, and also the clarification of the taxon’s systematics. Based on some characters considered fundamental in the Anillini taxonomy (Giachino 2005; Giachino and Vailati 2011), the species is here assigned to *Gilesdytes* gen. nov.

##### Differential diagnosis.

Small species (TL mm 1.29), easily distinguishable from *G. ethelianus* sp. nov. by the discal pore located towards the elytral edge, and from *G. pardooanus* sp. nov. by the lateral edge of the pronotum smooth, non-denticulate, near the basal angle.

##### Description of the ♂.

TL 1.29 mm. ***Body*** elongated, depigmented, yellow-testaceous; integument shiny, with evident microsculpture, and covered with sparse and relatively long pubescence.

***Head*** relatively large, narrower than the pronotum. Labium bearing a median tooth. Antennae short, robust, moniliform, just exceeding the base of the pronotum when stretched backwards. Fronto-clypeal furrow slightly distinct; subrectilinear anterior margin of epistome.

***Pronotum*** subrectangular, slightly transverse (max. width / max. length ratio = 1.04), maximum width at the anterior third, very wide basal border, slightly wider than anterior border. Pronotum sides anteriorly poorly arcuate, posteriorly subrectilinear, gently convergent, and laterally smooth, non-denticulate, but distinctly emarginated before the base. Anterior angles rounded, not prominent; posterior angles not protruding externally, rounded at the vertex. Disc slightly convex, with sparse and relatively long pubescence; median groove very shallow, slightly marked. Marginal groove relatively wide and flat, much enlarged near the base; anterior marginal setae inserted inside the marginal groove, approximately on the anterior third; basal setae inserted approximately on the posterior angles.

***Legs*** long and slender, with metatrochanters normal and metafemora unarmed. Males with two dilated protarsomeres without adhesive phanerae.

***Elytra*** subrectangular, very elongated (max. length/max. width ratio = 1.94), maximum width at the anterior 7^th^, not emarginated before apex. Disc poorly convex; integument shiny, with evident microsculpture and relatively long, sparse, upright pubescence. Humeri marked, almost right; post-humeral margin denticulate, with distinct crenulations up to the base of the apical 5^th^ of the elytron; elytral apices not separately rounded. Marginal groove wide and evident up to the 8^th^ pore of the umbilicate series.

***Chaetotaxy***: scutellar pore large, foveate. Umbilicate series with 1^st^, 2^nd^, and 3^rd^ pores of the humeral group not equidistant, 2^nd^ and 3^rd^ pores closest; 4^th^ pore clearly farther from the 3^rd^ one and placed at the end of the basal third of the elytron; 5^th^ pore placed just after the middle length of the elytron; 5^th^ and 6^th^ pores spaced out ca. 1/3 of the distance from 6^th^ and 7^th^; 7^th^, 8^th^, and 9^th^ pores almost equidistant and slightly displaced onto the disc, 8^th^ pore placed after the 9^th^ one. A single discal seta placed laterally towards the edge of the disc and located between the 7^th^ and 9^th^ pores.

***Aedeagus*** (Fig. 39) relatively small, median lobe long, slender, subrectilinear, with evident basal bulb; ventral margin poorly and gently curved from the basal bulb to the apex; apical blade evident, but short. Endophallus with a median, sinuate, sclerified phanera. Left paramere slender and very elongated, reaching the distal fifth of the median lobe, and bearing two setae; right paramere shorter than the left one.

**Female.** See Baehr and Main (2016).

##### Distribution.

*Gilesdytes vixsulcatus* is known from two localities approximately 85–90 km NW of Newman, Pilbara, WA. The distance between the two collection points is ca. 30 km.

#### Gilesdytes pardooanus sp. nov.

Taxon classificationAnimaliaColeopteraCarabidae

58350F0D-FB39-5420-A21F-A7B88BC2A649

http://zoobank.org/7603781D-4B6F-4CA0-AC1B-B29077C99F4D

Fig. 40

##### Type locality.

WA, Pilbara, 100 km E of Port Hedland, Pardoo Mine, 20°17'19.72"S, 119°10'38.88"E.

##### Type series.

HT ♀, WA, Pilbara, 100 km E of Port Hedland, Pardoo Mine, (WGS84) 20°17'19.72"S, 119°10'38.88"E, N. Stevens, 02 Oct. 2007, Trog. Trap (PDRC779-LN743), Western Australian Museum Entomology Reg. no. 82634 (WAM).

##### Differential diagnosis.

Medium sized species (TL mm 2.11), easily distinguishable from *G. ethelianus* sp. nov. by the discal pore placed towards the elytral edge, and from *G. vixsulcatus* by the denticulate lateral edge of the pronotum before the basal angle.

##### Description of the HT ♀.

TL 2.11 mm. ***Body*** elongated, depigmented, testaceous; integument shiny, with evident microsculpture, covered with very sparse and short pubescence.

***Head*** relatively large, narrower than pronotum. Labium bearing a median tooth. Antennae short and delicate, moniliform, just exceeding the base of the pronotum when stretched backwards. Fronto-clypeal furrow slightly distinct; anterior margin of the epistome subrectilinear.

***Pronotum*** subrectangular, slightly transverse (max. width / max. length ratio = 1.05), maximum width at the anterior third, with very wide basal border, slightly wider than anterior border, pronotum sides anteriorly poorly arcuate, and laterally-posteriorly denticulate before the basal angles. Anterior angles obtuse, slightly prominent; posterior angles sub squared not protruding, gently rounded at the vertex. Disc slightly convex, with very sparse and short pubescence; median groove very shallow, faintly marked. Marginal groove relatively wide and flat, very enlarged near the base; anterior marginal setae inserted inside marginal groove, approximately on the anterior fifth; basal setae inserted approximately on the posterior angles.

***Legs*** long and slender, with metatrochanters normal and metafemora unarmed.

***Elytra*** subrectangular, very elongated (max. length/max. width ratio = 1.92), maximum width at the anterior 3^rd^, slightly emarginated before apex. Disc poorly convex; integument shiny, with evident microsculpture and very short, sparse and upright pubescence. Humeri hardly marked, almost right; post-humeral margin denticulate, with distinct crenulations up to the 9^th^ pore of the umbilicate series; elytral apices not separately rounded. Marginal groove wide and evident up to the 8^th^ pore of the umbilicate series.

***Chaetotaxy***: scutellar pore large, foveate. Umbilicate series with 1^st^, 2^nd^, and 3^rd^ pores of the humeral group almost equidistant; 4^th^ pore slightly displaced onto the disc and clearly farther from the 3^rd^ pore, placed at the end of the basal third of the elytron; 5^th^ pore placed just after the middle length of the elytron; 5^th^ and 6^th^ ones spaced out ca. the 1/3 of the distance from 6^th^ and 7^th^; 7^th^, 8^th^, and 9^th^ not equidistant, 8^th^ and 9^th^ pores closest, 8^th^ pore located after the 9^th^ one; 8^th^ slightly displaced onto the disc. One single discal seta laterally placed towards the edge, at the level of the 9^th^ pore.

**Male.** Unknown.

##### Etymology.

The name of the species comes from the type locality Pardoo, in the Pilbara region (WA).

##### Distribution.

*Gilesdytes pardooanus* sp. nov. is known only from the type locality (Pardoo Mine), 100 km E of Port Hedland, Pilbara, WA.

#### Gilesdytes ethelianus sp. nov.

Taxon classificationAnimaliaColeopteraCarabidae

29EADDB5-1486-56E2-B03F-27A50A3C8BEC

http://zoobank.org/DEE7D80F-D1DD-4951-876F-2D67E1357976

Figs 41 , 42

##### Type locality.

WA, Pilbara, 11 km ENE Newman, Ethel Gorge, 23°18'22.176S, 119°51'41.652E.

##### Type series.

HT ♂ (remains), WA, Pilbara, 11 km ENE Newman, Ethel Gorge, 23°18'22.176S, 119°51'41.652E (WGS84), P. Bell, S. Catomore, 05 Nov. 2010, Stygo. Net haul; (BHP021_W262-10:0697e) Western Australian Museum Entomology Reg. no. 82608 (WAM).

##### Note.

The only specimen collected had head, prothorax and legs missing (excluding one trochanter), however the remaining characters available were not comparable with other species described, and the presence of aedeagus allowed us to describe the new species.

The taxon *ethelianus* sp. nov., based on elytra and aedeagus morphology, is provisionally assigned to the genus *Gilesdytes*.

##### Differential diagnosis.

Small sized species (estimated TL mm 1.30), easily distinguishable from *G. pardooanus* sp. nov. and from *G. vixsulcatus* by the discal pore placed in the middle of elytron, not located towards its edge.

##### Description of the HT ♂.

Estimated TL mm 1.30.

***Elytra*** (Fig. 41) subrectangular (total length = mm 0.77), very elongated (max. length/max. width ratio = 1.94), maximum width near the middle, slightly emarginated before apex. Disc poorly convex; shiny, integument testaceous, with evident microsculpture and very short, sparse and upright pubescence. Humeri hardly marked, almost right; post-humeral margin denticulate, with distinct crenulation up to the 8^th^, 9^th^ pores of the umbilicate series; elytral apices not separately rounded. Marginal groove wide and evident up to the 7^th^ pore of the umbilicate series.

***Chaetotaxy***: scutellar pore large, foveate. Umbilicate series with the 1^st^, 2^nd^ and 3^rd^ pores of the humeral group almost equidistant; 4^th^ pore clearly farther from the 3^rd^, placed after the end of the basal third of the elytron; 5^th^ pore placed just after the middle length of the elytron; 5^th^ and 6^th^ pores spaced out ca. the 1/3 of the distance from 6^th^ and 7^th^ ones; 7^th^, 8^th^ and 9^th^ pores not equidistant, with 8^th^ and 9^th^ pores closest. Due to setae missing it is impossible to determine if the 8^th^ pore is placed after the 9^th^ one; 7^th^ pore displaced onto the disc. One single discal seta not laterally located towards the edge and placed at the level of the 7^th^ pore.

***Metatrochanters*** unarmed in the male.

***Aedeagus*** (Fig. 42) relatively small, median lobe long, slender, subrectilinear, with basal bulb well-shaped; ventral margin poorly and gently curved from basal bulb to apex; apical blade short and poorly evident. Endophallus with a subapical, bisinuate, sclerified phanera. Parameres missing.

##### Etymology.

The name comes from the Ethel Gorge type locality.

##### Distribution.

*Gilesdytes ethelianus* sp. nov. is known only from the type locality (Ethel Gorge), 11 km ENE of Newman, Pilbara, WA.

#### Pilbaradytes gen. nov.

Taxon classificationAnimaliaColeopteraCarabidae

C37247B2-D72D-59BF-A50B-0BD4B4872D7F

http://zoobank.org/2E0A19CC-11A0-4CBF-B48C-3A9DBB8235B3

Figs 43–45

##### Type species.

*Pilbaradytes abydosianus* sp. nov.

##### Diagnosis.

Included species strongly characterised by: lacking longitudinal elytral grooves, pronotum basal border as wide as anterior border, with sides posteriorly not or only slightly sinuate, basal seta of pronotum absent, one elytral discal seta placed near elytral edge, 8^th^ pore of the umbilicate series placed after the 9^th^ one (sensu Giachino and Vailati 2011), metafemora not dentate, labium tooth absent, median lobe of the aedeagus long and curved with parameres long and slender. Differs from *Magnanillus* Baher, 2017 by the absence of elytral longitudinal grooves, and by a single elytral discal seta (3 setae in *Magnanillus*).

##### Description.

A genus of Anillini with species of a medium size (TL mm 2.12–2.23), anophthalmous. Integument depigmented but well sclerified, with strong microsculpture and covered with short and sparse pubescence.

***Head*** as wide as, or narrower, than the pronotum base; mandibles short and simple, without hyperplasias. Maxillary palps ovoidal, swollen. Labium transverse, articulated; mentum not fused with submentum. Labial tooth absent. Antennae not strictly moniliform (with relatively elongated antennomeres).

***Pronotum*** squared, with sides smooth or serrulate, not or only slightly sinuate towards the basal third. Basal angles right or obtuse, sharp, not rounded; basal border as wide as or wider than anterior margin; presence of only one marginal seta, posterior seta absent.

***Elytra*** subrectangular, elongated, not separately rounded, not truncate and slightly emarginated apically; convex, without longitudinal grooves. Elytral striae missing (except for the sutural stria). Lateral margin, starting from the humeral area, hardly serrulate up to the level of 6^th^ pore of the umbilicate series.

Scutellar pore present, large and umbilicate; umbilicate series of type B (sensu Jeannel 1963; Giachino and Vailati 2011) with the 8^th^ pore placed after the 9^th^ one; disc bearing one seta strictly located near the elytral edge.

***Legs*** relatively long and slender. In female, unarmed pro- and metafemora; metatrochanters normal; male legs unknown.

***Aedeagus*** relatively large; median lobe long, curved, with basal bulb of normal size. Parameres long, slender, bearing two apical setae. Endophallus without sclerified phanerae.

##### Etymology.

Composite name coming from the Pilbara region with the suffix -*dytes* (diver). Gender masculine.

##### Species included.

Currently two species belong to this genus:

*Pilbaradytes abydosianus* sp. nov.

*Pilbaradytes webberianus* sp. nov.

**Figures 43–45.**
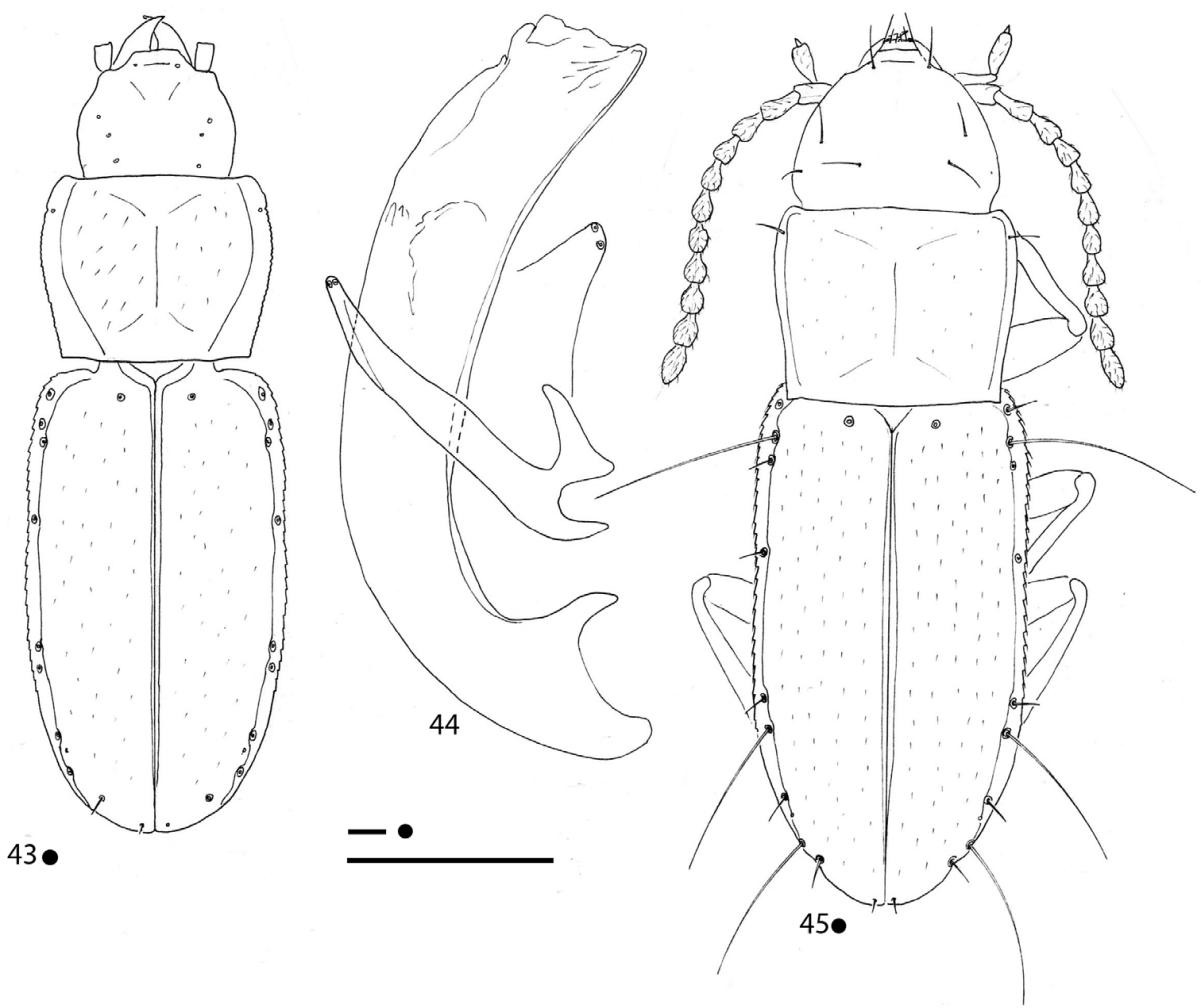
*Pilbaradytes* gen. et spp. nov., habitus (**43, 45**) aedeagus in lateral view (**44**) **43, 44***P. abydosianus* sp. nov., HT ♂ **45***P. webberianus* sp. nov., HT ♀. Scale bars: 0.1 mm.

### Key to the species of *Pilbaradytes*

**Table:** 

1	Head large, as wide as pronotum base	***P. webberianus* sp. nov.**
–	Head small, narrower than pronotum base	***P. abydosianus* sp. nov.**

#### Pilbaradytes abydosianus sp. nov.

Taxon classificationAnimaliaColeopteraCarabidae

F9C87688-C6FB-570D-9B47-9CFC4A2D27A1

http://zoobank.org/5D8F783C-2B27-44D3-8743-882147B1EA60

Figs 43 , 44

##### Type locality.

WA, Pilbara, 100 km SE of Port Hedland, Abydos deposit, 21°08'31.1"S, 119°06'53.99"E.

##### Type series.

HT ♂, WA, Pilbara, 100 km SE of Port Hedland, Abydos Mine, 21°08'31.1"S, 119°06'53.99"E (WGS84) 10 Nov. 2008, P. Bell, Trog trap; (ABY01_ABRC029-10LN6261), Western Australian Museum Entomology Reg. no. 82624 (WAM).

##### Differential diagnosis.

Medium sized species (TL mm 2.12), easily distinguishable from *P. webberianus* sp. nov. by smaller head and by serrulated lateral edges of pronotum.

##### Description of the HT ♂.

TL mm 2.12. ***Body*** elongated, depigmented, yellow-testaceous; shiny integument, with evident microsculpture, covered with very sparse and short pubescence.

***Head*** small, narrower than the base of pronotum. Labium without tooth. Antennae missing. Fronto-clypeal furrow slightly distinct; subrectilinear anterior margin of epistome.

***Pronotum*** subrectangular, slightly transverse (max. width / max. length ratio = 1.22), maximum width at the base of the anterior third, very wide basal border, as wide as the anterior border, pronotum sides poorly arcuate anteriorly, subrectilinear and gently convergent posteriorly, laterally completely serrulate from the anterior seta to the basal angles, not emarginated before the base. Anterior angles rounded, only slightly prominent; posterior angles sharp and obtuse, not protruding. Disc slightly convex, with very sparse and short pubescence; median groove very shallow, slightly marked. Marginal groove wide and flat, very enlarged near the base; anterior marginal setae inserted inside the marginal groove, approximately on the anterior fifth; basal seta lacking.

***Legs*** missing.

***Elytra*** subrectangular, very elongated (max. length/max. width ratio = 1.79), maximum width at the middle, slightly emarginated in the pre-apical zone. Disc convex; shiny integument, with evident microsculpture and short, very sparse, upright pubescence. Humeri very marked, but rounded; post-humeral margin denticulate, with distinct crenulations up to 6^th^ pore of the umbilicate series; elytral apices not separately rounded. Marginal groove wide and evident up to the 8^th^ pore of the umbilicate series.

***Chaetotaxy***: scutellar pore large, foveate. Umbilicate series with 1^st^, 2^nd^, and 3^rd^ pores of the humeral group not equidistant, 2^nd^ and 3^rd^ pores closest; 4^th^ pore clearly farther from the 3^rd^ one and placed at the end of the basal third of the elytron; 5^th^ pore placed well after the middle length of the elytron; 5^th^ and 6^th^ pores spaced out ca. the 1/3 of the distance from 6^th^ and 7^th^ ones; 7^th^, 8^th^, and 9^th^ pores almost equidistant, 8^th^ pore slightly displaced onto the disc and placed after the 9^th^ one. One single discal seta laterally placed near the edge, midway between the 7^th^ and the 9^th^ pores.

***Aedeagus*** (Fig. 44) relatively large, median lobe long, tubular, regularly curved, with basal bulb evident; ventral margin gently curved from the basal bulb to the apex; apical blade evident, but short. Endophallus without any sclerified phanerae. Left parameres slender and very elongated, reaching the distal fourth of the median lobe, and bearing two setae; right paramere stout and shorter than the left one, and bearing two setae.

**Female.** Unknown.

##### Etymology.

The name comes from the type locality Abydos deposit.

##### Distribution.

*Pilbaradytes abydosianus* sp. nov. is known only from the type locality 100 km SE of Port Hedland, Pilbara, WA.

#### Pilbaradytes webberianus sp. nov.

Taxon classificationAnimaliaColeopteraCarabidae

87567848-E1EA-5A7E-BBC6-2358334AEF81

http://zoobank.org/CBE0FAF1-D8D7-4008-83FD-A3117741C8DC

Fig. 45

##### Type locality.

WA, Pilbara, 150 km SE of Port Hedland, Mount Webber Mine, 21°32'16.81"S, 119°17'17.88"E.

##### Type series.

HT ♀, WA, Pilbara, 150 km SE of Port Hedland, Mount Webber Mine, 21°32'16.81"S, 119°17'17.88"E (WGS84), E.S. Volschenk, S. Catomore, 05"Sept. 2010; Trog. trap (COF01-MW025-10:9656) Western Australian Museum Entomology Reg. no. 82616 (WAM). PTT: 1 ♀, WA, Pilbara, 150 km SE of Port Hedland, Mount Webber Mine, 21°32'08.09"S, 119°17'18.4"E (WGS84), E.S. Volschenk, S. Catomore, 04"Sept. 2010, Trog. trap (COF01-MW090-10:9708) Western Australian Museum Entomology Reg. no. 82617 (CGi).

##### Differential diagnosis.

Medium size species (TL mm 2.23), easily distinguishable from *P. abydosianus* sp. nov. by the larger head and the smooth lateral edges of the pronotum.

##### Description of the HT ♀.

TL mm 2.23. ***Body*** elongated, depigmented, yellow-testaceous; shiny integument, with evident microsculpture, covered with very sparse and short pubescence.

***Head*** small, approximately as wide as the base of the pronotum. Labium without tooth. Antennae with relatively elongated antennomeres, short, just exceeding the base of the pronotum when stretched backwards. Fronto-clypeal furrow slightly distinct; subrectilinear anterior margin of epistome.

***Pronotum*** subrectangular, slightly transverse (max. width / max. length ratio = 1.23), maximum width at the base of the anterior fifth, with very wide basal border, as wide as the anterior border, pronotum sides poorly arcuate anteriorly, subrectilinear and very slightly sinuated posteriorly, laterally completely smooth from anterior seta to basal angles, not emarginated before the base. Anterior angles rounded, only slightly prominent; posterior angles right, sharp, not protruding. Disc slightly convex, with very sparse and short pubescence; median groove very shallow, slightly marked. Marginal groove narrow and flat, not enlarged near the base; anterior marginal setae inserted inside marginal groove, approximately on the anterior seventh; basal seta absent.

***Legs*** relatively long and slender. In females, unarmed pro- and metafemora; normal metatrochanters.

***Elytra*** subrectangular, very elongated (max. length/max. width ratio = 1.85), maximum width at the middle, lateral sides slightly emarginated at the end of the basal third and in the pre-apical zone. Disc convex; shiny integument, with evident microsculpture and short, sparse and upright pubescence. Humeri very marked, but rounded; post-humeral margin denticulate, with distinct crenulations up to 6^th^ pore of the umbilicate series; elytral apices not separately rounded. Marginal groove wide and evident up to the 9^th^ pore of the umbilicate series.

***Chaetotaxy***: scutellar pore large, foveate. Umbilicate series with 1^st^, 2^nd^, and 3^rd^ pores of the humeral group not equidistant, 2^nd^ and 3^rd^ pores closest; 4^th^ pore clearly farther from the 3^rd^ one and placed at the end of the basal third of the elytron; 5^th^ pore placed well after the middle length of the elytron; 5^th^ and 6^th^ pores spaced out ca. half of the distance from 6^th^ and 7^th^; 7^th^, 8^th^, and 9^th^ pores almost equidistant, 8^th^ and 9^th^ pores closer to each other than 7^th^ and 9^th^; 8^th^ pore placed after the 9^th^ one. One single discal seta laterally placed near the edge, midway between the 7^th^ and the 9^th^ pores.

**Male.** Unknown.

##### Etymology.

The name comes from the type locality Mount Webber, in the Pilbara region.

##### Distribution.

*Pilbaradytes webberianus* sp. nov. is known only from Mount Webber, 150 km SE of Port Hedland, Pilbara, WA.

#### Bylibaraphanus gen. nov.

Taxon classificationAnimaliaColeopteraCarabidae

A7EA4BBF-AA5A-5FCC-835B-872CB6F7A3D2

http://zoobank.org/3F222A9D-BAD5-442A-9210-9830EC9EBF15

Figs 46–48

##### Type species.

*Gracilanillus currani* Baehr & Main, 2016

##### Diagnosis.

Included species strongly characterised by: longitudinal elytral grooves absent, pronotum with basal border as wide as or narrower than anterior border, and sides not or only slightly posteriorly sinuate, basal seta of pronotum present, one elytral discal seta present, 8^th^ pore of the umbilicate series placed after the 9^th^ pore (sensu Giachino and Vailati 2011), metafemora not dentate, labial tooth absent, median lobe of aedeagus long and curved, parameres long and slender. *Bylibaraphanus* differs from *Magnanillus* Baher, 2017 by elytra with a single discal seta (3 in *Magnanillus*) and smaller body size. It differs from *Hesperanillus* Baher & Main, 2016 by head narrower than pronotum, and pronotum less cordiform. It differs from *Gracilanillus* Baher & Main, 2016 by the absence of longitudinal elytral grooves.

##### Description.

Genus with small size species (TL mm 1.43–1.50), anophthalmous. Depigmented integument, medium sclerified, with strong microsculpture and dense pubescence.

***Head*** wider than pronotum base; mandibles short and simple, without hyperplasias. Maxillary palps ovoidal, swollen. Labium transverse, articulated; mentum not fused with submentum. Labial tooth absent. Antennae moniliform.

***Pronotum*** squared, pronotum sides smooth, not or only slightly sinuate at the basal third. Basal angles acute or obtuse, sharp or rounded; basal border as wide as, or narrower, than anterior border; basal seta present.

***Elytra*** convex, subrectangular, elongated, separately rounded, not truncate, apically slightly emarginated, and without longitudinal grooves. Elytral striae missing (except for the sutural stria). Lateral margin, starting from the humeral area, hardly serrulate up to the level of the 7^th^ or 9^th^ pore of the umbilicate series.

Scutellar pore present, large and umbilicate; umbilicate series of type B (sensu Jeannel 1963; Giachino and Vailati 2011) with the 8^th^ pore placed after the 9^th^ one; disc bearing one seta (posterior) in the central area of the disc.

***Legs*** relatively long and slender. Pro- and metafemora unarmed; metatrochanters normal; two protarsomeres slightly dilated and without adhesive phanerae in males.

***Aedeagus*** relatively large; median lobe relatively long, curved, not restricted before the basal bulb, basal bulb of normal size. Parameres long, slender, bearing two or three apical setae. Endophallus with sclerified phanera.

##### Etymology.

Combined name formed by *Bylibara* (aboriginal noun for the Pilbara region) and the suffix -*phanus* taken from *Illaphanus* genus. The gender of the name is masculine.

##### Species included.

The following two species belong to this genus:

*Bylibaraphanus currani* (Baehr & Main, 2016)

*Bylibaraphanus cundalinianus* sp. nov.

**Figures 46–48.**
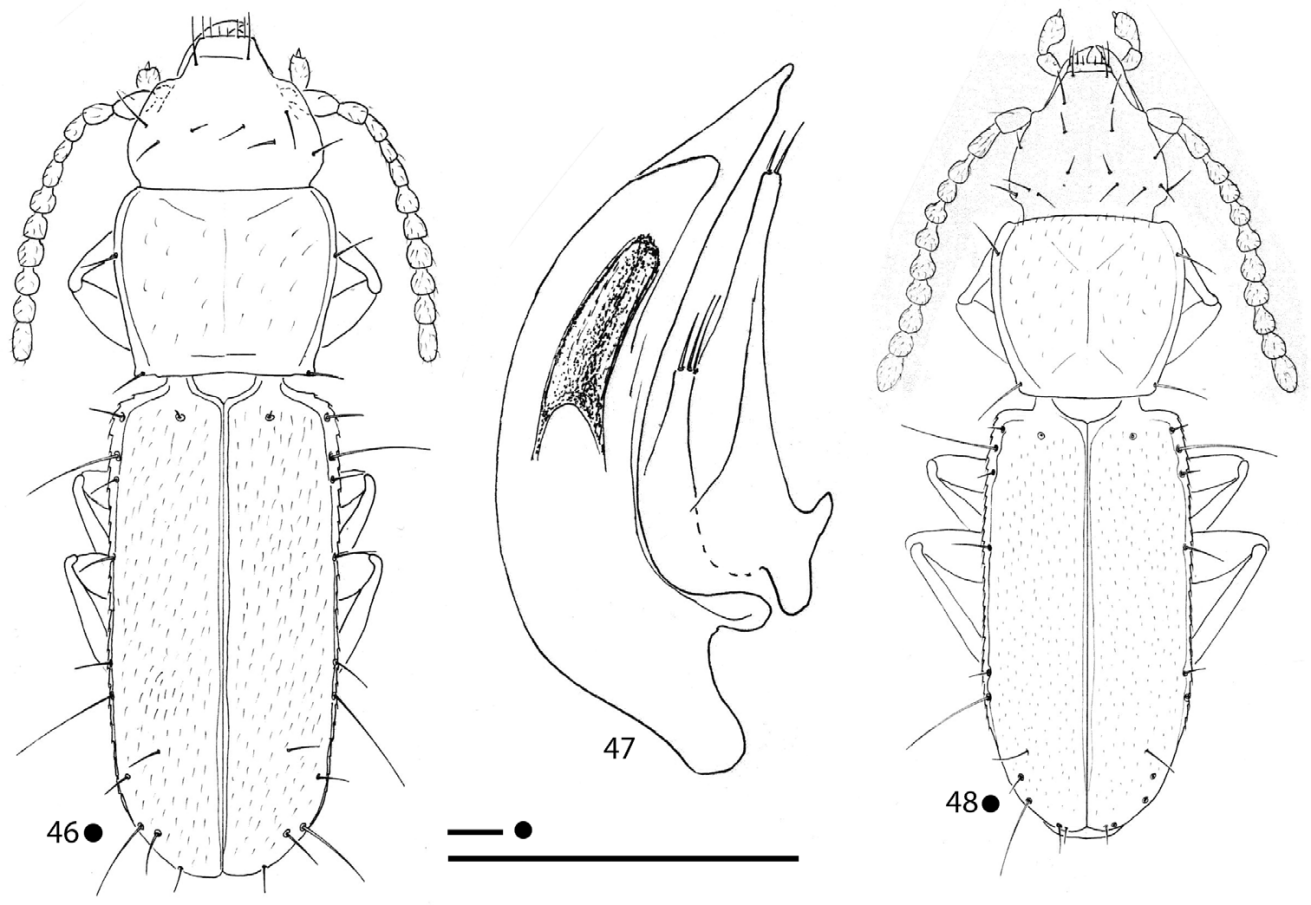
*Bylibaraphanus* gen. et spp. nov., habitus (**46, 48**) aedeagus in lateral view (**47**) **46, 47***B. currani* (Baehr and Main), ♂ **48***B. cundalinianus* sp. nov., HT ♀. Scale bars: 0.1 mm.

### Key to the species of *Bylibaraphanus*

**Table:** 

1	Large (mm 1.47–1.50). Basal angles of pronotum acute and protruding	***B. currani* (Baehr & Main)**
–	Small (mm 1.43) Basal angles of pronotum obtuse and not protruding	***B. cundalinianus* sp. nov.**

#### Bylibaraphanus currani

Taxon classificationAnimaliaColeopteraCarabidae

(Baehr & Main, 2016)

1E96F607-6F52-56B0-9F5F-92FD1484D4E2

Figs 46 , 47

##### Material examined.

2 ♂♂ 3 ♀♀, WA, Pilbara, 65 km NW of Tom Price, Cloudbreak Mine, 22°20'26.40"S, 119°25'50.70"E (WGS84), J. Cocking, M. Scanlon, 15 Mar. 2011, Trog. Net scrape (GNGC05589), Western Australian Museum Entomology Reg. no. 8269–82673 (WAM, CGi).

##### Note.

Baehr and Main’s description (2016) is correct and detailed, although we noted a few inaccuracies and their description omits some important characters fundamental to the systematics of Anillini which, including male genitalia, are described as follows.

##### Differential diagnosis.

Species of small size (TL mm 1.47–1.50), easily distinguishable from *B. cundalinianus* sp. nov. by the basal angles of pronotum acute and protruding.

##### Redescription.

TL mm 1.49–1.50 ♂♂ 1.47–1.49 ♀♀. ***Body*** elongated, depigmented, yellow-testaceous; integument shiny, with evident microsculpture, covered with relatively long pubescence.

***Head*** large, slightly narrower than the base of pronotum. Labium without tooth. Antennae short, just exceeding the base of the pronotum when stretched backwards. Fronto-clypeal furrow slightly distinct; subrectilinear anterior margin of epistome.

***Pronotum*** subrectangular, slightly transverse (max. width / max. length ratio = 1.22), maximum width at the base of the anterior fourth, with very wide basal border, as wide as the anterior border, pronotum sides, anteriorly poorly arcuate, and posteriorly subrectilinear shortly and gently sinuated, laterally completely smooth from anterior seta to basal angles, not emarginated before the base. Anterior angles rounded, not prominent; posterior angles acute, sharp, protruding. Disc slightly convex, with very sparse and relatively long pubescence; median groove very shallow, slightly marked. Marginal groove narrow and flat, not particularly enlarged near the base; anterior marginal setae inserted inside marginal groove, at ca. the anterior third; basal setae inserted approximately on posterior angles.

***Legs*** long and slender, with normal metatrochanters and unarmed metafemora; two protarsomeres slightly dilated and without adhesive phanerae in males.

***Elytra*** subrectangular, very elongated (max. length/max. width ratio = 2.09), with parallel sides, slightly emarginated before apex. Disc convex; integument shiny, with evident microsculpture and short, very dense and upright pubescence. Humeri very marked, but rounded; post-humeral margin denticulate, with distinct crenulations up to 7^th^ pore of the umbilicate series; elytral apices separately rounded. Marginal groove narrow and evident up to the 7^th^ pore of the umbilicate series.

***Chaetotaxy***: scutellar pore large, foveate. Umbilicate series with 1^st^, 2^nd^, and 3^rd^ pores of the humeral group not equidistant, 2^nd^ and 3^rd^ pores closest; 4^th^ pore clearly farther from the 3^rd^ one and placed at the end of the basal third of the elytron; 5^th^ pore placed well after the middle length of the elytron; 5^th^ and 6^th^ pores spaced out ca. the half distance between 6^th^ and 7^th^ pores; 7^th^, 8^th^ and 9^th^ pores slightly displaced onto the disc and not equidistant, 8^th^ placed after the 9^th^ one. One single discal seta in the central area of the disc and placed approximately half way between the 6^th^ and 7^th^ pores.

***Aedeagus*** (Fig. 47) relatively large, median lobe, in lateral view, relatively long and triangularly restricted at apex, regularly curved and not restricted before the basal bulb, basal bulb of normal size. Ventral margin gently curved from basal bulb to apex, emarginated just before the apex; apical blade evident, very long. Endophallus with a large, concave, well sclerified phanera in the middle area. Left parameres slender and very elongated, reaching the distal fourth of the median lobe, and bearing two setae; right paramere shorter than left one and bearing three setae.

##### Distribution.

*B. currani* is known only from Cloudbreak Mine, 65 km NW of Tom Price, Pilbara, WA.

#### Bylibaraphanus cundalinianus sp. nov.

Taxon classificationAnimaliaColeopteraCarabidae

762D38FC-597D-56FA-B9BA-6B714E234FD1

http://zoobank.org/4F3DB9E3-D6AC-4732-B224-775BD1AEE712

Fig. 48

##### Type locality.

WA, Pilbara, 200 km E of Port Hedland, Yarrie Mining Area, Cundaline Ridge, 20°32'36"S, 120°09'35"E.

##### Type series.

HT ♀, WA, Pilbara, 200 km E of Port Hedland, Yarrie Mining Area, Cundaline Ridge, 20°32'36"S, 120°09'35"E, 29 May 2009, Subterranean Ecology, bore-hole CU0046R, scraping, Western Australian Museum Entomology Reg. no. 72022 (WAM).

##### Differential diagnosis.

Small sized species (TL mm 1.43), easily distinguishable from *B. currani* by obtuse and not protruding basal angles of the pronotum.

##### Description of the HT ♀.

TL mm 1.43. ***Body*** elongated, depigmented, yellow-testaceous; integument shiny, with evident microsculpture, covered with short pubescence.

***Head*** large, slightly narrower than the base of the pronotum. Labium without tooth. Antennae short, just exceeding the base of the pronotum when stretched backwards. Fronto-clypeal furrow slightly distinct; subrectilinear anterior margin of epistome.

***Pronotum*** subrectangular (max. width / max. length ratio = 1.06) maximum width at the base of the anterior fourth, with basal border narrower than the anterior border, pronotum sides, anteriorly poorly arcuate, posteriorly subrectilinear and slightly sinuated, laterally completely smooth from anterior seta to basal angles, not emarginated before the base. Anterior angles rounded, slightly prominent; posterior angles obtuse, rounded, not protruding. Disc slightly convex, with very sparse and relatively short pubescence; median groove very shallow, slightly marked. Marginal groove relatively narrow and flat, not particularly enlarged near the base; anterior marginal setae inserted inside the marginal groove, approximately on the anterior fifth; basal setae inserted approximately on posterior angles.

***Legs*** long and slender, with metatrochanters normal and metafemora unarmed.

***Elytra*** subrectangular, very elongated (max. length/max. width ratio = 1.98), with parallel sides, slightly emarginated before apex. Disc convex; integument shiny, with evident microsculpture and very short, very dense and upright pubescence. Humeri very marked, squared; post-humeral margin denticulate, with distinct crenulations up to the discal pore; elytral apices separately rounded. Marginal groove narrow and evident up to the discal pore.

***Chaetotaxy***: scutellar pore large, foveate. Umbilicate series with 1^st^, 2^nd^,and 3^rd^ pores of the humeral group almost equidistant; 4^th^ pore clearly farther from the 3^rd^ one and placed at the end of the basal third of the elytron; 5^th^ pore placed well after the middle length of the elytron; 5^th^ and 6^th^ pores spaced out ca. 1/3 of the distance between 6^th^ and 7^th^ pores; 7^th^, 8^th^, and 9^th^ pores not equidistant, 8^th^ placed after the 9^th^ one; 7^th^ slightly displaced onto the disc. One single discal seta in the central area of the disc and placed at ca. 2/3 of the distance between the 6^th^ and 7^th^ pores.

**Male.** Unknown.

##### Etymology.

The name comes from the type locality: Cundaline Ridge.

##### Distribution.

*Bylibaraphanus cundalinianus* sp. nov. is known only from the type locality Cundaline Ridge, in the Yarrie Mining Area, 200 km E of Port Hedland, Pilbara, WA.

#### Angustanillus

Taxon classificationAnimaliaColeopteraCarabidae

Baehr & Main, 2016

168EFD30-E449-59B9-8C04-1473AD35E32B

Figs 49–51

##### Type species.

*Angustanillus striatipennis* Baehr & Main, 2016

##### Diagnosis.

Included species strongly characterised by: body very elongated, posterior supraorbital seta and longitudinal elytral grooves absent, pronotum with basal border narrower than anterior border, and sides posteriorly distinctly crenelated, basal seta of pronotum present, one or two elytral discal seta present, 8^th^ pore of the umbilicate series located after the 9^th^ pore (sensu Giachino and Vailati 2011), metafemora dentate in males (state unknown in females), labial tooth absent, median lobe of the aedeagus long, slender and curved, parameres long and slender.

##### Note.

Describing *Angustanillus*Baehr and Main (2016) mention the presence of two setae on the elytral disc, one located in the fifth basal section, and one in the fourth apical section. The species we describe here has only one seta, situated in the posterior one. Considering the taxonomic importance of the number of setae in establishing the systematics of a taxon in Anillini (Giachino 2005; Giachno and Vailati 2011), together with the difficulties in identifying these setae without a proper preparation and observation with a microscope at high magnification, we consider the presence of one seta as the most probable character in this genus. However, in the genus diagnosis we account for the possible presence of one or two discal setae.

Additionally, in the genus diagnosis we indicate the presence of a large metafemoral tooth, at least in the male. The female of *A. armatus* sp. nov. is unknown to date, and the female holotype is known only for *A. striatipennis*Baehr and Main (2016), therefore still it is not possible to confirm the presence of this character state in both sexes of the two species. We hence re-describe the genus *Angustanillus* integrating the new characters presented by *A. armatus* sp. nov.

##### Redescription.

Included large size species (TL > mm 2.40), anophthalmous. Depigmented and sclerified integument, with strong microsculpture (sometimes formed by distinct, deep, large punctures) and covered with dense pubescence.

***Head*** wider than the pronotum base; mandibles short and simple, without hyperplasias. Maxillary palps ovoidal, swollen. Labium transverse, articulated; mentum not fused with submentum. Labial tooth absent. Antennae not strictly moniliform (with relatively elongated antennomeres).

***Pronotum*** elongated, with sides distinctly crenelated in the basal fourth, not sinuate in the basal third. Pronotum basal border slightly narrower than the anterior border; basal angles sharp and squared; basal seta present, placed before basal angles.

***Elytra*** subrectangular and elongated, separately rounded, not or distinctly emarginated apically; convex, without longitudinal grooves. Elytral striae present (4 striae) or missing (except for the sutural stria). Lateral margin, starting from the humeral area, hardly serrulate up to the level of 8^th^ pore of the umbilicate series.

Scutellar pore present, large and umbilicate; umbilicate series of type B (sensu Jeannel 1963; Giachino and Vailati 2011), 8^th^ pore placed after the 9^th^ pore; disc bearing one (posterior) or two setae in the central area of the disc.

***Legs*** relatively long and slender. Male metafemora distally armed with a long, internal spur; metatrochanters normal; two protarsomeres dilated and without adhesive phanerae in males.

***Aedeagus*** relatively large; median lobe long, curved, not restricted before the basal bulb that is of normal size. Parameres relatively long and robust, bearing two setae: one apical and one subapical. Endophallus without sclerified phanerae.

##### Species included.

The following two species belong to this genus:

*Angustanillus striatipennis* Baehr & Main, 2016

*Angustanillus armatus* sp. nov.

**Figures 49–51.**
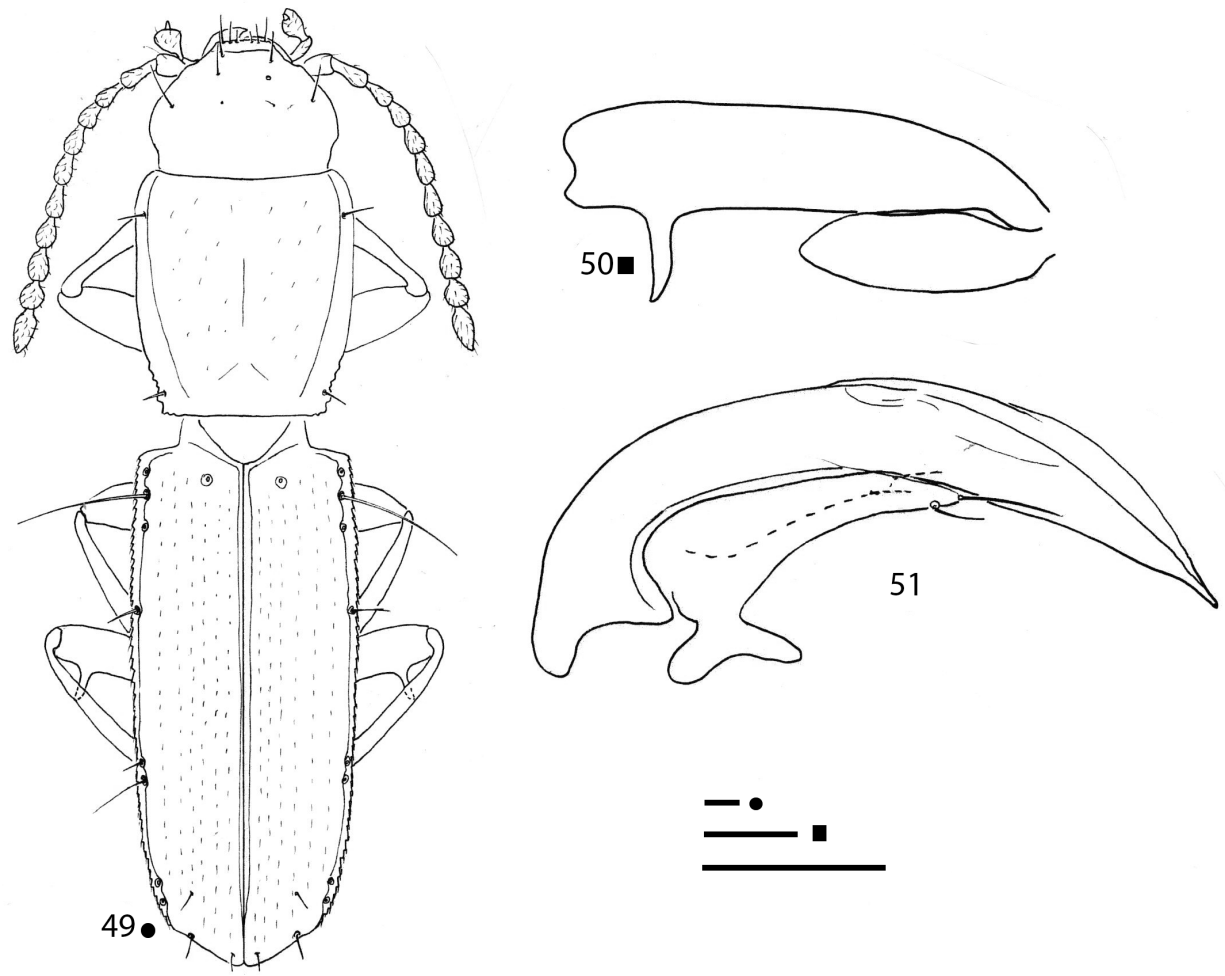
*Angustanillus armatus* sp. nov., HT ♂ **49** habitus **50** left metafemur and metatrochanter in dorsal view **51** aedeagus in lateral view. Scale bars: 0.1 mm.

### Key to the species of *Angustanillus*

**Table:** 

1	Elytra with four distinct striae; two discal setae. Surface of pronotum and elytra with distinct, deep and large punctures. Pronotum less elongated (ratio length/width 0.86)	***A. striatipennis* Baehr & Main**
–	Elytra without distinct striae, but with a series of short setae longitudinally aligned; one discal seta. Surface of pronotum and elytra without distinct, deep and large punctures. Pronotum more elongated (ratio length/width 0.90)	***A. armatus* sp. nov.**

#### Angustanillus armatus sp. nov.

Taxon classificationAnimaliaColeopteraCarabidae

AD307FF0-6115-5B70-B16A-BF354B9C696C

http://zoobank.org/096C5FA9-4853-4E91-8AB8-D9A704D4A7AE

Figs 49–51

##### Type locality.

WA, Pilbara, 38 km W of Pannawonica, Mesa B, 21°39'36"S, 115°57'20"E.

##### Type series.

HT ♂, WA, Pilbara, 38 km W of Pannawonica, Mesa B (bore hole MEBRC0021), 21°39'36"S, 115°57'20"E (GPS WGS84), March-May 2005; M. Greenham, D. Kamien, L. Mould, Western Australian Entomology Reg. no. 64217 (WAM).

##### Differential diagnosis.

Large sized species (TL mm 2.66), easily distinguishable from *A. striatipennis* by: elytra without distinct striae, but with a series of short and longitudinally aligned setae; only one discal seta; pronotum and elytra surface without distinct punctures; and more elongated pronotum.

##### Description of the HT ♂.

TL mm 2.66. ***Body*** elongated, depigmented, yellow-testaceous; integument shiny, with evident microsculpture, covered with very short pubescence.

***Head*** large, slightly wider than the base of the pronotum. Labium without tooth. Antennae short, just exceeding the base of the pronotum when stretched backwards. Fronto-clypeal furrow of frontal clypeo slightly distinct; anterior margin of the epistome subrectilinear.

***Pronotum*** elongated (max. width / max. length ratio = 0.90), maximum width at the middle, basal border slightly narrower than the anterior border, pronotum sides poorly arcuate, distinctly crenelated at the posterior fourth, emarginated before the base. Anterior angles rounded, not prominent; posterior angles sharp, squared, not protruding. Disc slightly convex, with very sparse and short pubescence; median groove very shallow, slightly marked. Marginal groove wide and flat, enlarged near the base; anterior marginal setae inserted inside the marginal groove, approximately on the anterior fifth; basal setae inserted before the posterior angles.

***Legs*** long and slender, with metatrochanters normal and metafemora (Fig. 50) armed with a long, internal spur; two protarsomeres dilated and without adhesive phanerae in male.

***Elytra*** subrectangular, very elongated (max. length/max. width ratio = 2.28), with parallel sides, slightly emarginated before apex. Disc convex; integument shiny, with evident microsculpture and short, longitudinally aligned and upright pubescence. Humeri very marked, squared; post-humeral margin denticulate, with a distinct crenulation up to the 8^th^ pore of the umbilicate series; elytral apices separately rounded. Marginal groove narrow and evident up to the 8^th^ pore of the umbilicate series.

***Chaetotaxy***: scutellar pore large, foveate. Umbilicate series with 1^st^, 2^nd^, and 3^rd^ pores of the humeral group almost equidistant; 4^th^ pore clearly farther from the 3^rd^ one and placed at the end of the basal third of the elytron; 5^th^ pore placed at the end of median third of the elytron; 5^th^ and 6^th^ pores spaced out ca. 1/4 of the distance from 6^th^ and 7^th^ pores; 7^th^, 8^th^, and 9^th^ pores not displaced onto the disc and not equidistant, 8^th^ pore placed after the 9^th^ one. One single discal seta in the central area of the disc and placed approximately at the level of the 9^th^ pore.

***Aedeagus*** (Fig. 51) relatively large, median lobe, in lateral view, long and restricted at apex, regularly curved and not restricted before the basal bulb, basal bulb of normal size. Ventral margin curved from basal bulb to apex, gently emarginated just before the apex; apical blade evident, but short. Endophallus without sclerified phanerae. Parameres relatively long and robust, bearing two setae: one apical and one subapical; right paramere shorter than the left one.

##### Etymology.

The name comes from the Latin word *armato* (which means armed) to highlight the presence of a large spur on metafemora.

##### Distribution.

*Angustanillus armatus* sp. nov. is known only from the type locality Mesa B, 38 km W of Pannawonica, Pilbara, WA.

## Discussion

**Figure 52.**
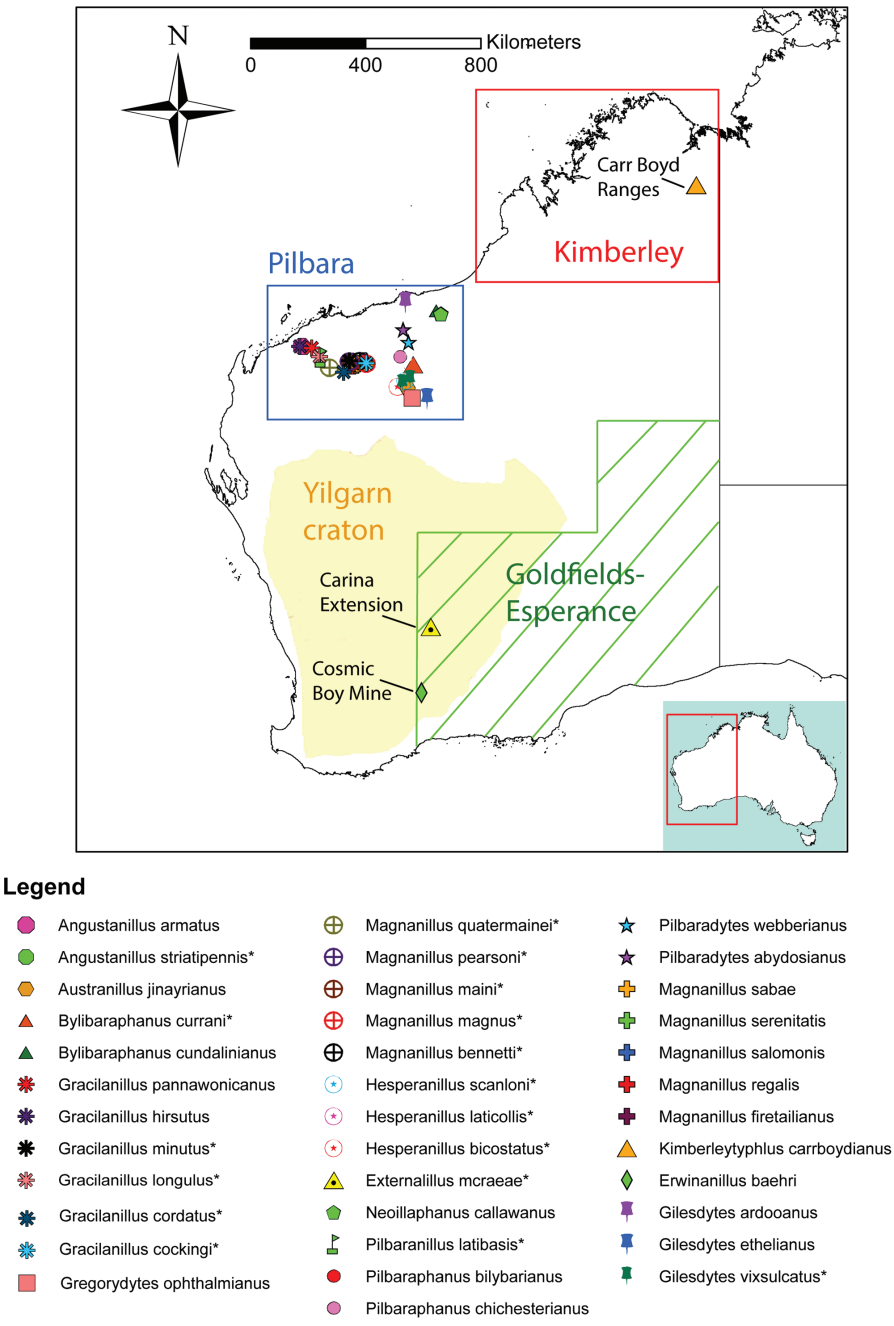
Distribution of all currently described Anillini species in Western Australia. See Fig. 53 for an enlargement of Pilbara region. Species described by Baehr and Main (2016) are marked with an asterisk (*).

**Figure 53.**
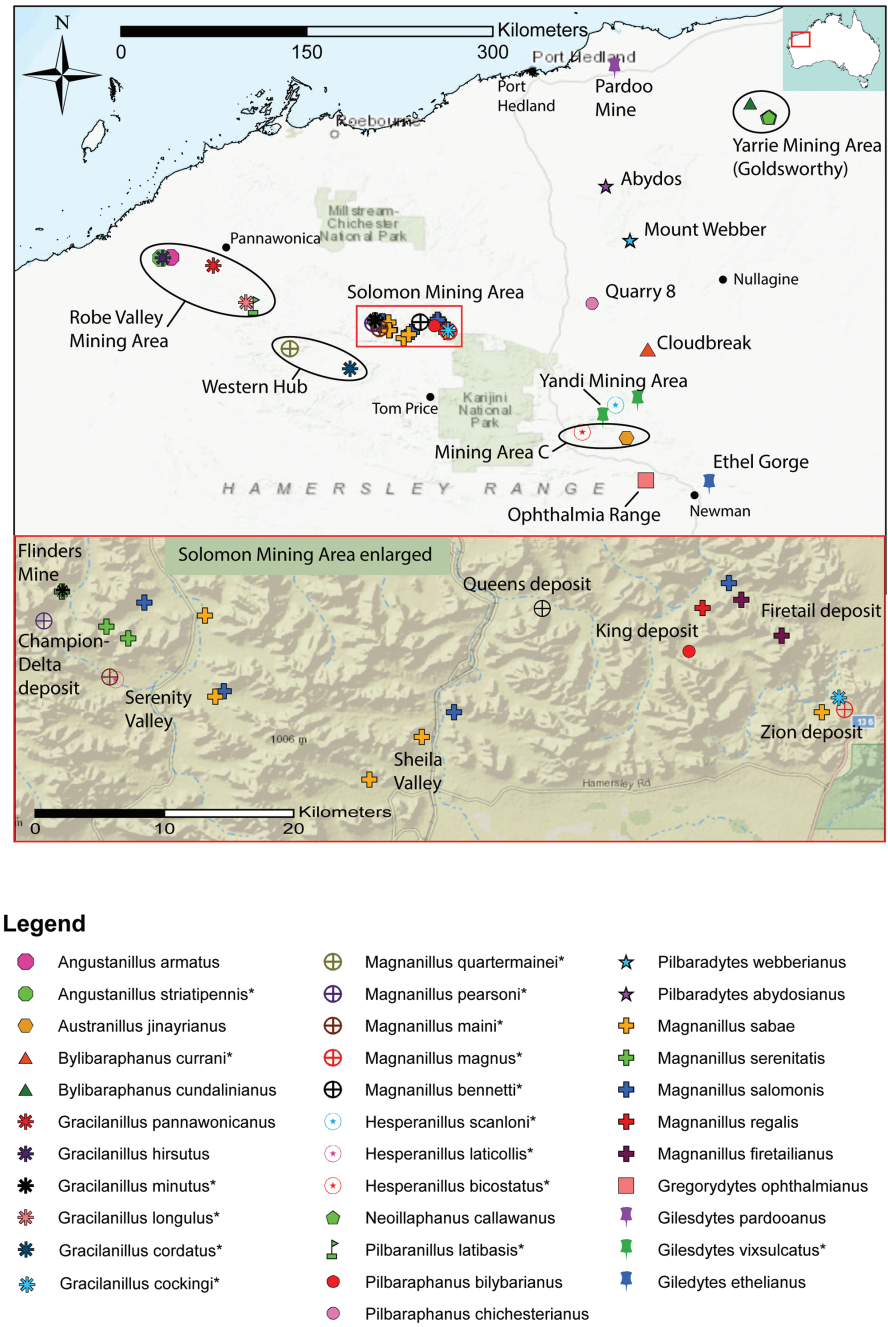
Distribution of all currently described Anillini species in the Pilbara region, Western Australia. Species described by Baehr and Main (2016) are marked with an asterisk (*).

The Anillini tribe is very diverse in the semi-arid climate regions of Western Australia, with 15 genera and 37 species described to date (Table 1). As noted by Baehr and Main (2016) some genera have a wide distribution within the Pilbara region (Fig. 52), however all species appear to have very restricted ranges and were only ever collected from a single mining area (Table 1, Fig. 53). Three monotypic genera (*Gregorydytes*, *Neoillaphanus*, *Pilbaranillus*) are so far known only from a single mining spot. For the seven genera detected at two or more mining areas, the greatest distance between mining areas where any single genus was detected ranged from 48 km (*Pilbaradytes*) to 343 km (*Gilesdytes*), and the median greatest distance was 166 km. For nearly all species, their sampled range lies within a single mineral deposit exploration area, which at most, is generally only a few tens of square kilometres in area. The species which displayed the greatest sampled ranges were *Magnanillus salomonis*, *M. sabae* and *M. serentitatus*. These three species were detected in multiple deposits spanning 45 to 50 km across the comparatively extensive Solomon Mining Area which encompasses several ranges interspersed by valleys (Fig. 53).

**Table 1.** Checklist of Western Australian Anillini, listed alphabetically by genus and species, along with their collection region, mining area and locality / deposit.

Genus	Species	Region	Mining area	Locality / deposit
* Erwinanillus *	*E. baehri*	Goldfields	Forrestania	Cosmic Boy
* Externalillus *	*E. mcraeae*	Goldfields	Carina	Carina Extension
* Kimberleytyphlus *	*K. carrboydianus*	Kimberley	Carr-Boyd Ranges	Carr-Boyd Ranges
* Angustanillus *	*A. armatus*	Pilbara	Robe Valley	Mesa B
	*A. striatipennis*			Mesa A
* Austranillus *	*A. jinayrianus*		Area C	Jinayri
* Bylibaraphanus *	*B. cundalinianus*		Yarrie	Cundaline
	*B. currani*		Cloudbreak	Cloudbreak
* Gilesdytes *	*G. ethelianus*		Newman	Ethel Gorge
	*G. pardooanus*		Pardoo	Pardoo
	*G. vixsulcatus*		Yandi	Marillana Creek, Ministers North
* Gracilanillus *	*G. cockingi*		Solomon	Zion
	*G. cordatus*		Western Hub	Western Hub
	*G. minutus*		Solomon	Flinders
	*G. hirsutus*		Robe Valley	Mesa A
	*G. longulus*			West Pit
	*G. pannawonicanus*			Mesa K
* Gregorydytes *	*G. ophthalmianus*		Newman	Ophthalmia Range
* Hesperanillus *	*H. bicostatus*		Area C	Area C
	*H. laticollis*		Solomon	Flinders
	*H. scanloni*		Yandi	Phil’s Creek
* Magnanillus *	*M. bennetti*		Solomon	Queens
	*M. firetailianus*			Firetail
	*M. magnus*			Zion
	*M. maini*			Flinders
	*M. pearsoni*			Flinders
	*M. quartermainei*		Western Hub	Western Hub
	*M. regalis*		Solomon	Kings
	*M. sabae*			Zion, Serenity, Sheila Valley
	*M. salomonis*			Kings, Firetail, Serenity, Sheila
	*M. serenitatis*			Champion, Firetail, Delta, Serenity
* Neoillaphanus *	*N. callawanus*		Yarrie	Callawa
* Pilbaradytes *	*P. abydosianus*		Abydos	Abydos
	*P. webberianus*		Mount Webber	Mount Webber
* Pilbaranillus *	*P. latibasis*		Robe Valley	Dragon
* Pilbaraphanus *	*P. bilybarianus*		Solomon	Kings
	*P. chichesterianus*		Chichester Ranges	Quarry 8

The apparent highly localized distribution patterns for the Anillini recorded in our study may be partly a sampling artefact coinciding with the highly localized and restricted distribution of drill holes within the sampled mining areas. This is an inherent limitation involved with sampling these habitats which can only be accessed through drill holes. Despite this practical limitation, extensive and repeated sampling in other large Pilbara mining areas with many drill holes dispersed over multiple deposits has demonstrated with high confidence that many groups of subterranean arthropods do have extremely localised ranges (see Halse 2018; Halse and Pearson 2014). Other groups of subterranean Coleoptera show similar patterns of high species richness and short-range endemism in the Pilbara, including the Physocrotaphini and Zuphiini tribes (Baehr 2014a, 2014b), as well as undescribed troglomorphic morpho-species in the Curculionoidea, Zopheridae (subfamily Colydiinae), and Staphylinidae (subfamily Pselaphinae) (Subterranean Ecology Pty Ltd, unpublished data).

The number of Anillini species detected at individual mining areas / localities ranged from one to thirteen, however the median number of species was only two (n = 11). The Solomon Mining Area, which recorded 13 species in four genera, and the Robe Valley, which recorded six species in three genera, stand out as hotspot areas for hypogean Anillini (Table 1). *Magnanillus* is the most speciose anilline genus in the region, which has radiated within the Solomon Mining Area where nine of the ten described species occur. *Gracilanillus* is the next most speciose genus with six species described to date, from the Robe Valley, Solomon, and Western Hub mining areas. Considering the high local richness, and high turnover between localities, it is likely that many more anilline species remain to be discovered in the Pilbara and other semi-arid regions with suitable habitat underground conditions.

The discovery of this rich fauna of Anillini in Western Australia’s semi-arid regions is important because it significantly expands the ecology of this group, which is otherwise almost exclusively endogean, as noted by Baehr and Main (2016). The Western Australian fauna of Anillini, which is known only from subterranean habitats, contrasts with the eastern Australian fauna which is known only from endogean habitats. Globally, Anillini are mostly represented by endogean species (Jeannel 1937; 1963; Giachino and Vailati 2011, 2012; Giachino 2008, 2015), including the Australian fauna (Giachino 2005; Baehr 2018). Only a few taxa have been collected in deeper hypogean habitats, and these taxa display typical subterranean characters (e.g., Español and Comas 1985; Ortuño and Sendra 2007). Similarly, the species described here, and in Baehr and Main (2016), show morphological specialisations to the hypogean habitat (Giachino and Vailati 2018), such as increased body size, modified metatrochanters and metafemora (hypertrophic and dentate), modified elytral microsculpture, and presence of elytral striae (usually obsolete in Anillini). Other commonly expressed characters of specialised subterranean carabids (for example Trechini and Zuphiini) are absent in these hypogean Anillini, such as elongation of legs and antennae (Giachino and Vailati 2010; Baehr 2014b).

One hypothesis that may explain this extraordinary diversity of relict hypogean Anillini in the Pilbara is that an ancient endogean Anillini fauna gradually adapted to deeper subterranean habitats to escape the progressively drought on the ground surface developing at the end of the Tertiary which has driven the present aridification of the Australian continent. This classical ‘climatic relict’ theory has been invoked to explain the high richness and short-range endemism patterns displayed by several other groups of relict terrestrial and aquatic subterranean fauna in Western Australia (Harvey 2002; Humphreys 1993, 2008, 2017). The scenario involves populations of hygrophilic epigean lineages colonising underground habitats where moisture and food conditions remained favourable, followed by extinction of their surface counterparts as surface conditions became inhospitable, thus facilitating allopatric speciation. Phylogenetic analyses conducted on troglobitic pseudoscorpions (Harrison et al. 2014), schizomids (Abrams et al. 2019; Harms et al. 2018), isopods (Javidkar et al. 2018) and springtails (Guzik et al. 2020) suggested multiple colonisations by putative epigean ancestors and species radiations coinciding with aridification between 15 and 1.75 Ma. Despite the lack of molecular data to estimate the time of divergence amongst the Anillini taxa described so far, the ‘climatic relict’ hypothesis may help to explain their ecological and evolutionary shift to subterranean habitats in semi-arid environments. This time frame is well within the presence of Anillini in Australia (considered a Gondwanan lineage) starting from 85 Ma (Giachino 2005; Baehr and Main 2016). However, the preponderance of Anillini to become speciose and have short distribution ranges may be more a consequence of the group’s low vagility rather than climatic relictualisation processes, because endogean Anillini in the mesic climate regions of eastern Australia are similarly diverse and localised. Consideration of ‘adaptive shift’ and parapatric speciation processes (sensu Howarth 2019) may also help our understanding of the evolution of subterranean Anillini, since this largely endogean group is intrinsically pre-adapted to subterranean life, and the ‘adaptive shift’ model and classical theory of troglobiont evolution via climatic vicariance are not necessarily mutually exclusive. Molecular studies of Australian Anillini are needed to reconstruct the phylogeny of the group and to estimate the time of divergences for hypogean taxa to investigate both ‘climatic relict’ and ‘adaptive shift’ models for their evolution.

A major challenge confronting many contemporary systematists is how to integrate standard taxonomic research with conservation outcomes. Harvey et al. (2011) argue that ‘whole of biota’ surveys (that include all invertebrates) are rarely fundable and are logistically impossible, and that concentrated research on some of the most vulnerable elements in the landscape – short-range endemics, including troglofauna and stygofauna – can help to enhance conservation and research outcomes. Because the Anillini are inherently short-range endemics, they are priority candidates for conservation in environmental impact assessments (EIA’s). New Anillini species continue to be collected during the course of pre-mining EIA surveys in Western Australia’s semi-arid regions. Further taxonomic and phylogenetic studies of this group are needed to better understand the patterns of diversity and distribution, and their conservation requirements.

## Supplementary Material

XML Treatment for Erwinanillus

XML Treatment for Erwinanillus baehri

XML Treatment for Gracilanillus

XML Treatment for Gracilanillus hirsutus

XML Treatment for Gracilanillus pannawonicanus

XML Treatment for Gregorydytes

XML Treatment for Gregorydytes ophthalmianus

XML Treatment for Pilbaraphanus

XML Treatment for Pilbaraphanus chichesterianus

XML Treatment for Pilbaraphanus bilybarianus

XML Treatment for Magnanillus

XML Treatment for Magnanillus firetailianus

XML Treatment for Magnanillus sabae

XML Treatment for Magnanillus salomonis

XML Treatment for Magnanillus regalis

XML Treatment for Magnanillus serenitatis

XML Treatment for Neoillaphanus

XML Treatment for Neoillaphanus callawanus

XML Treatment for Kimberleytyphlus

XML Treatment for Kimberleytyphlus carrboydianus

XML Treatment for Austranillus

XML Treatment for Austranillus jinayrianus

XML Treatment for Gilesdytes

XML Treatment for Gilesdytes vixsulcatus

XML Treatment for Gilesdytes pardooanus

XML Treatment for Gilesdytes ethelianus

XML Treatment for Pilbaradytes

XML Treatment for Pilbaradytes abydosianus

XML Treatment for Pilbaradytes webberianus

XML Treatment for Bylibaraphanus

XML Treatment for Bylibaraphanus currani

XML Treatment for Bylibaraphanus cundalinianus

XML Treatment for Angustanillus

XML Treatment for Angustanillus armatus
